# Exploring the effectiveness of molecular subtypes, biomarkers, and genetic variations as first-line treatment predictors in Asian breast cancer patients: a systematic review and meta-analysis

**DOI:** 10.1186/s13643-024-02520-5

**Published:** 2024-04-04

**Authors:** Nurul Wafiqah Saipol Bahrin, Siti Nur Idayu Matusin, Aklimah Mustapa, Lu Zen Huat, Sriyani Perera, Mas Rina Wati Haji Abdul Hamid

**Affiliations:** 1 Pengiran Anak Puteri Rashidah Sa’adatul Bolkiah (PAPRSB) Institute of Health Sciences, Universiti Brunei Darussalam, Jalan Tungku Link, Gadong, BE1410 Negara Brunei Darussalam; 2 Halalan Thayyiban Research Centre, Universiti Islam Sultan Sharif Ali, Jalan Tutong, Sinaut, TB1741 Negara Brunei Darussalam; 3 Faculty of Medicine, University of Peradeniya, Peradeniya, Sri Lanka

**Keywords:** Breast cancer, Molecular subtypes, Biomarkers, Genetic variation, Neoadjuvant treatment, Pathological complete response, Asian patients, Systematic review

## Abstract

**Background:**

Breast cancer incidence has been on the rise significantly in the Asian population, occurring at an earlier age and a later stage. The potential predictive value of molecular subtypes, biomarkers, and genetic variations has not been deeply explored in the Asian population. This study evaluated the effect of molecular subtype classification and the presence or absence of biomarkers and genetic variations on pathological complete response (pCR) after neoadjuvant treatment in Asian breast cancer patients.

**Methods:**

A systematic search was conducted in MEDLINE (PubMed), Science Direct, Scopus, and Cochrane Library databases. Studies were selected if they included Asian breast cancer patients treated with neoadjuvant chemotherapy and contained data for qualitative or quantitative analyses. The quality of the included studies was assessed using the Newcastle Ottawa Scale. Following the random effects model, pooled odds ratios or hazard ratios with 95% confidence intervals for pCR were analysed using Review Manager Software. Heterogeneity between studies was assessed using Cochran’s *Q*-test and *I*^2^ test statistics.

**Results:**

In total, 19,708 Asian breast cancer patients were pooled from 101 studies. In the neoadjuvant setting, taxane-anthracycline (TA) chemotherapy showed better pCR outcomes in triple-negative breast cancer (TNBC) (*p*<0.0001) and human epidermal growth factor receptor 2 enriched (HER2E) (*p*<0.0001) than luminal breast cancer patients. Similarly, taxane-platinum (TP) chemotherapy also showed better pCR outcomes in TNBC (*p*<0.0001) and HER2E (*p*<0.0001). Oestrogen receptor (ER)-negative, progesterone receptor (PR)-negative, HER2-positive and high Ki-67 were significantly associated with better pCR outcomes when treated with either TA or TP. Asian breast cancer patients harbouring wildtype *PIK3CA* were significantly associated with better pCR outcomes when treated with TA in the neoadjuvant setting (*p=*0.001).

**Conclusions:**

In the neoadjuvant setting, molecular subtypes (HER2E and TNBC), biomarkers (ER, PR, HER2, HR, Ki-67, nm23-H1, CK5/6, and Tau), and gene (*PIK3CA*) are associated with increased pCR rates in Asian breast cancer patients. Hence, they could be further explored for their possible role in first-line treatment response, which can be utilised to treat breast cancer more efficiently in the Asian population. However, it needs to be further validated with additional powered studies.

**Systematic review registration:**

PROSPERO CRD42021246295.

**Supplementary Information:**

The online version contains supplementary material available at 10.1186/s13643-024-02520-5.

## Background

Breast cancer is one of the most prevalent and heterogeneous cancers that predominantly affect women. According to GLOBOCAN 2020 [1], 2.3 million women were diagnosed with breast cancer, and 685,000 had died from the disease. This is a trend expected to rise due to early screening and detection [1]. Early diagnosis of the disease and effective treatment are paramount to improve patients’ condition, mortality, survival outcome, and prognosis [2].

Biomarkers are utilised to determine the type of systemic treatment to be administered to cancer patients [3]. Biomarkers can be genetic and non-genetic; they can be detected through gene sequencing and conventional immunohistochemistry (IHC). Clinically, gene sequencing is not always readily available compared to the assessment of IHC for the biomarkers. The most defined therapeutic molecular classification of breast cancer is based on the status of oestrogen receptors (ER), progesterone receptors (PR), human epidermal growth factor receptor 2 (HER2), and Ki-67 [4–6]. An extensive molecular classification of breast cancer characterising each subtype was first proposed by Perou et al. [6]. Through the expression of 496 intrinsic genes, breast cancer classification was subgrouped as luminal, HER2+, normal-like, and basal-like [6]. Subsequently, the luminal subtype was further subgrouped into luminal A, luminal B, and luminal C [7]. After re-evaluation, the luminal subtype was reduced to luminal A and luminal B [8]. Despite the advances in molecular subtyping, the St. Gallen Consensus allows IHC assessment of ER, PR, HER2, and several biomarkers as a surrogate classification for the molecular subtype of breast cancer. The panel agreed to molecularly characterise the subtypes as luminal A, luminal B, HER2-enriched (HER2E), and triple-negative breast cancer (TNBC) [5].

Biomarkers offer a wide range of potential uses in cancer, including risk assessment, screening, differential diagnosis, prognosis, treatment outcome prediction, and monitoring disease progression [9]. Biomarkers can be prognostic or predictive; the former allows insight into the overall cancer outcome of the patient regardless of the therapy given, while the latter provides information on whether a patient will benefit from a particular treatment [10]. Consequently, this allows the discernment of aggressive and non-aggressive breast cancer, especially in later stages. Due to this, biomarkers remain an area that is actively under investigation and validation [11]. The role of ER, PR, HER2, and Ki-67 in breast cancer remains important as biomarkers, nevertheless there are other expressions worth investigating. Amongst the investigated biomarkers include Tau, nm-23-H1, CK5/6, and epidermal growth factor receptor (EGFR) [12]. In this study, we defined these IHC-expressed markers as biomarkers.

Despite not being clinically available in most countries, nowadays, genetic biomarkers (henceforth referred to as genetic variations in this study) have gained significant interest in oncology practice, as they can be used for targeted treatment selections [13]. The variation in the genetic component is understood as the changes in the DNA sequence in an individual’s genome, which occurs at different frequencies within an individual across the population [13]. Genetic variations can exist in various forms, such as single-nucleotide polymorphism (SNP), short insertions or deletions, large mutations, null alleles, and transposable elements [13]. Some of the commonly mutated genes associated with breast cancer include *TP53, PIK3CA*, *BRCA1,* and *BRCA2* [14]. Consequently, these genetic variations have been reported to affect drug metabolism and disease susceptibility [15].

The emergence of personalised and precision medicine (PPM) is thought to be a better-fit therapeutic approach for oncology. Separately, precision medicine is defined as a therapeutic approach based on selecting definite biomarkers that predict a targeted therapy’s efficacy in a specific group of patients. Although the term personalised medicine is often interchangeably used as its synonym, it refers to the justification of the therapeutical choices for each patient [16]. Hence, it provides an opportunity to offer a better-fit treatment tailored to each patient, fitting the discovery that not all cancer is the same and varies heavily in each individual depending on their genetic changes [17, 18]. In this study, we used both precision and personalised terms and defined PPM as an emerging practice of medicine that uses an individual’s genetic profile, derived from their disease diagnosis, which includes molecular subtypes, biomarkers, and genetic variations, to guide decisions made concerning their therapeutic approach or choice.

The efficacy of cancer treatment can be evaluated through pathological complete response (pCR), disease-free survival (DFS), and overall survival (OS) [19]. pCR, defined as the absence of any residual disease in the breast and lymph nodes, can be used to predict DFS and OS since it is typically used as an endpoint for novel neoadjuvant chemotherapy (NAC) to predict the therapeutic outcome in the long run [19]. In this study, we emphasised the attainment of pCR following NAC as the focused efficacy outcome.

Classifying breast cancer based on molecular subtypes is a growing clinical practice that warrants benefits [2, 20, 21] and it is primarily conducted in the West [22, 23]. There is limited information on this practice in the Asian population [24]. Breast cancer incidence in the Asian population occurs at an earlier age with later stages compared to the Western population [25]. Furthermore, there are differences in the genetic polymorphism, epigenetics, and environmental interplay, which may cause treatment outcomes to differ [25, 26]. Therefore, it is imperative to evaluate the association between breast cancer diagnoses and their response to treatment. This systematic review and meta-analysis aimed to assess the involvement of PPM in breast cancer treatment in the Asian population by evaluating the association between breast cancer treatment response, specifically pCR and NAC treatment provided to breast cancer patients based on their breast cancer molecular subtypes, biomarkers, and genetic variation characterisation.

## Methods

### Study design

This study protocol was registered at PROSPERO (CRD42021246295). The systematic review was conducted following the Preferred Reporting Items for Systematic Reviews and Meta-Analyses (PRISMA)-2020 checklist guideline (Additional file 1) [27].

### Inclusion and exclusion criteria

**Fig. 1:**
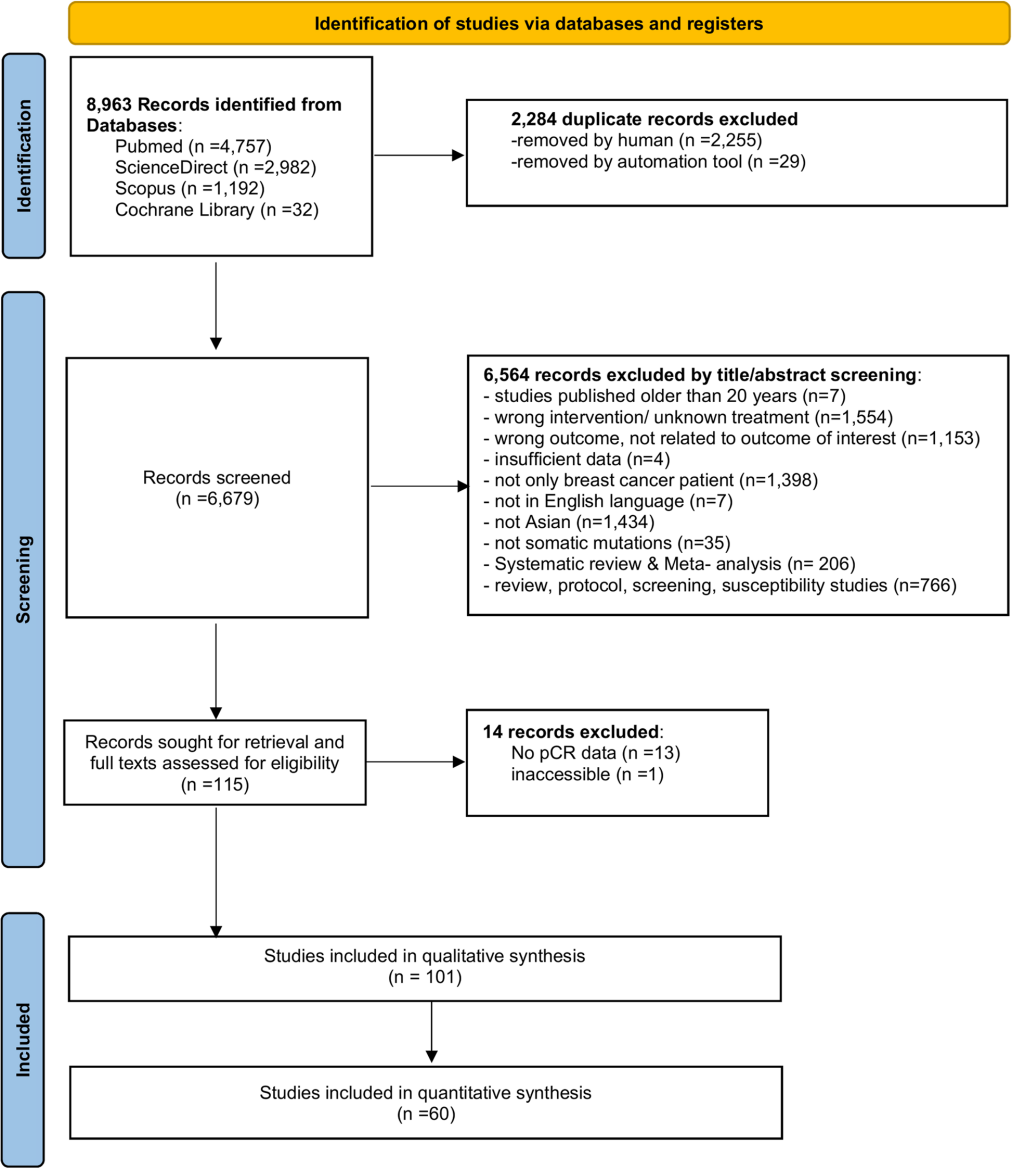
The PRISMA flowchart of literature search and study eligibility strategy

The type of studies included in this review consists of randomised trials, observational studies, case-control studies, and cohort studies written in the English language. All Asian breast cancer aged ≥ 18 years, who underwent systemic neoadjuvant chemotherapy treatment reporting the involvement of somatic genetic polymorphisms or biomarkers or molecular subtype classification on breast cancer treatment response were included. Studies were excluded if one or more of the following reasons were met: (1) non-Asian breast cancer cohort studies or non-breast cancer patients; (2) breast cancer patients undergoing other treatment that is not related to drug, i.e., radiotherapy, herbal medicine, and surgery; (3) studies with incomplete data for qualitative and/or quantitative synthesis.  Fig. 1 shows how the studies were searched and identified in the various databases and registers.

### Literature searches

The search was electronically performed in the MEDLINE (PubMed), Science Direct, Scopus, and Cochrane Library databases to retrieve articles that studied the role of molecular subtypes, biomarkers, and genetic variations in the outcome of breast cancer patients undergoing chemotherapy. Search terms were constructed based on patients, interventions and comparison, and outcomes (Additional file 2). The search term strategies were adapted for different databases utilising a combination of Medical Subject Heading (MeSH) and keywords that are relevant which can be found in the titles and abstract. Articles available from 1st January 2000 to 31st March 2021 were searched on MEDLINE, Science Direct, Scopus and the Cochrane Library. The search strategy was conducted from 24th March 2021 to 31st March 2021, with the finalised MeSH terms and search strategies were run again and harvested on the 31st of March 2021 to ensure the same results were generated. The search strategy can be found in detail for MEDLINE (Supplementary Table 2.2 in Additional file 2), Science Direct (Supplementary Table 2.3 in Additional file 2), Scopus (Supplementary Table 2.4 in Additional file 2), and the Cochrane Library (Supplementary Table 2.5 in Additional file 2).

### Data extraction

The retrieved search results were uploaded to Rayyan [28] for automated detection of duplicate records after manual removal with Mendeley, followed by initial eligibility screening of the abstracts and titles, applying the inclusion and exclusion criteria. The full-text articles for the remaining abstracts were retrieved and read for eligibility screening, applying the same inclusion and exclusion criteria. Two independent reviewers (NWSB and SNIM) conducted the eligibility screening. Any conflict was resolved by a third independent reviewer (AM). The included studies were randomly distributed amongst three independent reviewers (NWSB, SNIM, and AM) to extract relevant data using a standardised data extraction format using Microsoft Excel (Additional file 3).

For each study, the extracted parameters include the article information (article title, first author, year published, journal published, country, and year of recruitment), study design, study population and sample size, characteristics of patients in three variables (molecular subtypes, biomarkers, and genetic variations), and the pCR data in selected variables. Notably, in the absence of molecular subtype classification in the included studies, whenever possible, they were approximated through the available biomarkers detected through IHC data [5]. In this study, the molecular subtype classification is defined as luminal A (ER+, PR+, HER2−, and low Ki-67), luminal B (ER+, PR+, HER2+/HER2−, and high Ki-67), HER2E (ER−, PR−, and HER2+), and TNBC (ER−, PR−, and HER2−). Due to limited resources, the same reviewers (NWSB, SNIM, and AM) checked the data extraction process. Each reviewer is assigned a different article from the one they extracted in the previous stage. Notably, the reference lists of all selected publications and review articles were checked to identify further eligible studies missed in the MEDLINE (PubMed), Science Direct, Scopus, and Cochrane Library search.

All extracted data from selected parameters which include the study ID, country, quality assessment score, molecular subtypes, number of patients, number of pCR events in breast cancer patients treated with each NAC treatment, and crude and adjusted reported pCR association were combined and evaluated by the reviewer (NWSB) using standardised data synthesis excel sheets (Additional file 4). Two independent reviewers (SNIM and AM) cross-checked the synthesised data.

### Risk of bias in individual studies

The quality of all included studies was appraised independently by two reviewers (NWSB and SNIM) using a quality score system based on the Newcastle-Ottawa Scale (NOS) adapted for case-control studies or cohort studies (Additional file 5), with scores ranging from 0 (lowest) to 9 (highest). NOS utilised three domains: (1) selection, (2) comparability, and (3) exposure for case-control studies or outcomes for cohort studies [29]. The selection domain of case-control studies was appraised based on the description of study subjects and setting, while the exposure domain was appraised based on exposure measurement. Comparatively, the selection domain of cohort studies was appraised based on the description of study subjects and settings as well as the exposure measurement. The outcome domain was appraised by the outcome and follow-up assessment. The comparability domain of both case-control and cohort studies was appraised based on study design, analysis, and characterisation. Studies with an overall score of 0–3 were considered low quality, 4–5 were deemed medium quality, and 6 or above were regarded as high quality. Any discrepancies were resolved by consensus.

### Data analysis and synthesis

Data analysis was done using Review Manager Software (RevMan version 5.4.1) [30]. The odds ratio (OR), hazards ratio (HR), and their corresponding 95% confidence interval (95% CI) were assessed to evaluate the association between treatment response (pCR) and NAC treatment provided to breast cancer patients based on their molecular subtypes, biomarkers, and genetic variations. The strength of associations was estimated by calculating pooled ORs/HRs and 95% CIs, by which significance was stated using the *p*-value. A *p*-value <0.05 was considered statistically significant.

Two methods provided in RevMan were used. The first method utilises dichotomous outcomes parameters to measure the OR depicting the association between pCR and selected variables using the Mantel-Haenszel method [31] under the random effect model. The second method utilises the inverse-variance approach to evaluate the studies’ pooled association data, included under the random effects model using the DerSimonian and Laird method [32]. The preference for the favoured variable significantly associated with treatment response was based on the most frequent report in the pooled included studies (Supplementary Table 4.6 in Additional file 4). In any case of discordance of the 95% CI value entered intoRevMan obtained from the study, the software-generated value was used. Both crude and adjusted results were included in the analysis when available. For both methods, whenever possible or required, subgroup analysis was conducted. By convention, a pooled OR/HR <1 represents a worse treatment response, while a pooled OR/HR >1 represents a better treatment response for breast cancer patients.

### Meta-bias assessment

The between-study heterogeneity was assessed by Cochran’s chi-square-based *Q*-test and *I*^2^ index. It is considered statistically significant when *p*-value <0.05 and/or *I*^2^ index >50% [33]. The publication bias assessment was done when at least six studies were pooled for meta-analysis. The evaluation was made through visual inspection of funnel plot asymmetry and fail-Safe N test using the Rosenthal approach. Rank correlation and regression tests, using the standard error of the observed outcomes as predictors, are also used to check for funnel plot asymmetry.

## Results

### Article selection

A total of 8963 records were identified using different databases including MEDLINE, PubMed, ScienceDirect, Scopus and Cochrane Library. In total, 2284 records were excluded because of duplication, the removal was conducted through both manual removal and using an automation tool known as Rayyan. Then 6564 records were excluded after the initial title and abstract screening due to unmet inclusion criteria. Of the remaining 115 records, the full-text articles were carefully read, and 14 records were excluded due to insufficient pCR data and inaccessibility. Finally, 101 studies fulfilled the eligibility criteria and were included in the systematic review and meta-analysis (Fig. 1).

### Overview of included studies

Altogether, 19,708 Asian breast cancer patients were gathered from the 101 studies, with an average of 195 patients per study (Supplementary Table 4.1 in Additional file 4). The study population comprised 91 studies from Eastern Asia (China [8, 12, 34–115], Hong Kong [116, 117], Korea [118], and Japan [119–122]), 7 studies from Western Asia (Egypt [123], Iran [124], Turkey [125, 126], and Saudi Arabia [127–129]), 2 studies from Southeast Asia (Indonesia [130], Singapore and Malaysia [131]), and 1 study from South Asia (India [132]). The recruitment period for the patients enrolled in the studies ranged from 1991 to 2020. Most of the study cohorts followed hospital-based study design (76.2%) and clinical trials (14.9%), while 8.9% were not reported. 61.3% of the hospital-based study designs were conducted in unicenter, while 14.9% were in multicenter. Of the 101 studies, only 65 studies provided molecular subtype data (*n*=11361) - TNBC (29.2%), HER2E (20.7%), luminal B (13.2%), and luminal A (7.2%), while 1.6% were missing. Several studies did not categorise their luminal subtype into luminal A or B. Hence, they were reported as luminal-like (24.9%) in this study. Only 69 studies provided data on biomarkers, comprising the routinely analysed biomarkers—ER (25.1%), PR (25.1%), HR (5.7%), HER2 (27.4%), and Ki-67 (15.5%)—and several non-conventional biomarkers investigated specifically for the study including EGFR (11.0%), CK5/6 (8.0%), Tau (11.7%), Androgen Receptor (AR) (8.6%), PDL1 (3.6%), P-glycoprotein (P-gp) (3.3%), DNA topoisomerase II-alpha (TopoIIa) (3.2%), p53 (9.8%), and others (0.8–4.0%) (Supplementary Table 3.1 in Additional file 3). Meanwhile, 7 studies provided information on genetic variations and differential expression, where the common genetic variations reported were from *TP53* (15.7%), *PIK3CA* (24.6%), *MYC* (5.8%), *ERRB2* (5.8%), *CCDN1* (5.8%), *BRCA1* (15.7%), *BRCA2* (15.7%), and others (10.9%).

All the patients in the studies received NAC, and 25.0% of patients received follow-up adjuvant therapy. Collectively, NAC taxane-anthracycline (TA), taxane-platinum (TP), and taxane-anthracycline-platinum (TAP) combination were mentioned in 41.5%, 18.6%, and 1.5% of the studies, respectively. Meanwhile, 18.3% were treated with NAC anthracycline-based chemotherapy, 14.0% were treated with NAC taxane-based chemotherapy, and 8 studies did not provide specific treatment information. Some patients were treated concomitantly with targeted therapy and endotherapy (23.0%). The definition of pCR used in the included studies was mostly not reported according to any guideline (71.3%), with only 28.7% reported pCR following the Miller-Payne grading (17.8%), Kuerer et al. (1.9%), RECIST (2.9%), and other grading systems (6.1%) including the Ribero classification, Japanese Breast Cancer Society v2007, USFDA guideline, WHO criteria, and pathological TNM system.

Furthermore, 33 of the included studies conducted the multivariate analysis. Most of the included variables used to adjust the multivariate analysis were the commonly reported biomarkers (ER, PR, HER2, and Ki-67), age, tumour size and grade, age at diagnosis, lymph node stage, histological grade, body mass index (BMI), chemotherapy regimens, chemotherapy cycles, and other biomarkers and genetic variations unique to the study.

### Quality of the included studies

The quality assessment of the studies was presented in Supplementary Tables 6.1 and 6.2 (Additional file 6). Fourteen (14) case-control studies and 87 cohort studies were included in our systematic review. NOS scores for the 87 cohort studies ranged from 5 to 9 stars, and NOS scores for the 14 case-control studies ranged from 4 to 9 stars. No study was excluded since all studies scored ≥4 stars.

A summary of the risk of bias assessed on each question using the NOS for cohort and case-control studies is shown in Supplementary Figures 6.1 and 6.2 (Additional file 6). We considered both breast cancer treatment and characterisation as the most important factors for adjustment in the comparability domain because our study eligibility criteria required adjustment for the involvement of somatic genetic polymorphisms or biomarkers or molecular subtypes in breast cancer treatment response. Following this consideration, only 33% (*n*=29/87) cohort studies earned a star for comparability regarding pCR and breast cancer treatment, while 94% (*n*=82/87) cohort studies earned a star regarding pCR and breast cancer characterisation. As for case-control studies, 79% (*n*=11/14) studies earned a star for comparability regarding pCR and breast cancer treatment, while 93% (*n*=13/14) studies earned a star regarding pCR and breast cancer characterisation. Notably, when both adjustment factors were combined, only 31% (*n*=27/87) cohort studies and 71% (*n*=10/14) case-control studies earned both stars in the comparability domain. Amongst the cohort studies, evaluation of the selection of the non-exposed cohort was the question with the lowest count of stars, with only 32% (*n*=28/87) of the studies having a low risk of bias. Meanwhile, amongst the case-control studies, apart from the first adjustment in the comparability domain, the lowest count of stars was for the question evaluating the selection of controls, with 79% (*n*=11/14) of studies showing a low risk of bias.

### Association of breast cancer characterisation and treatment response

**Fig. 2:**
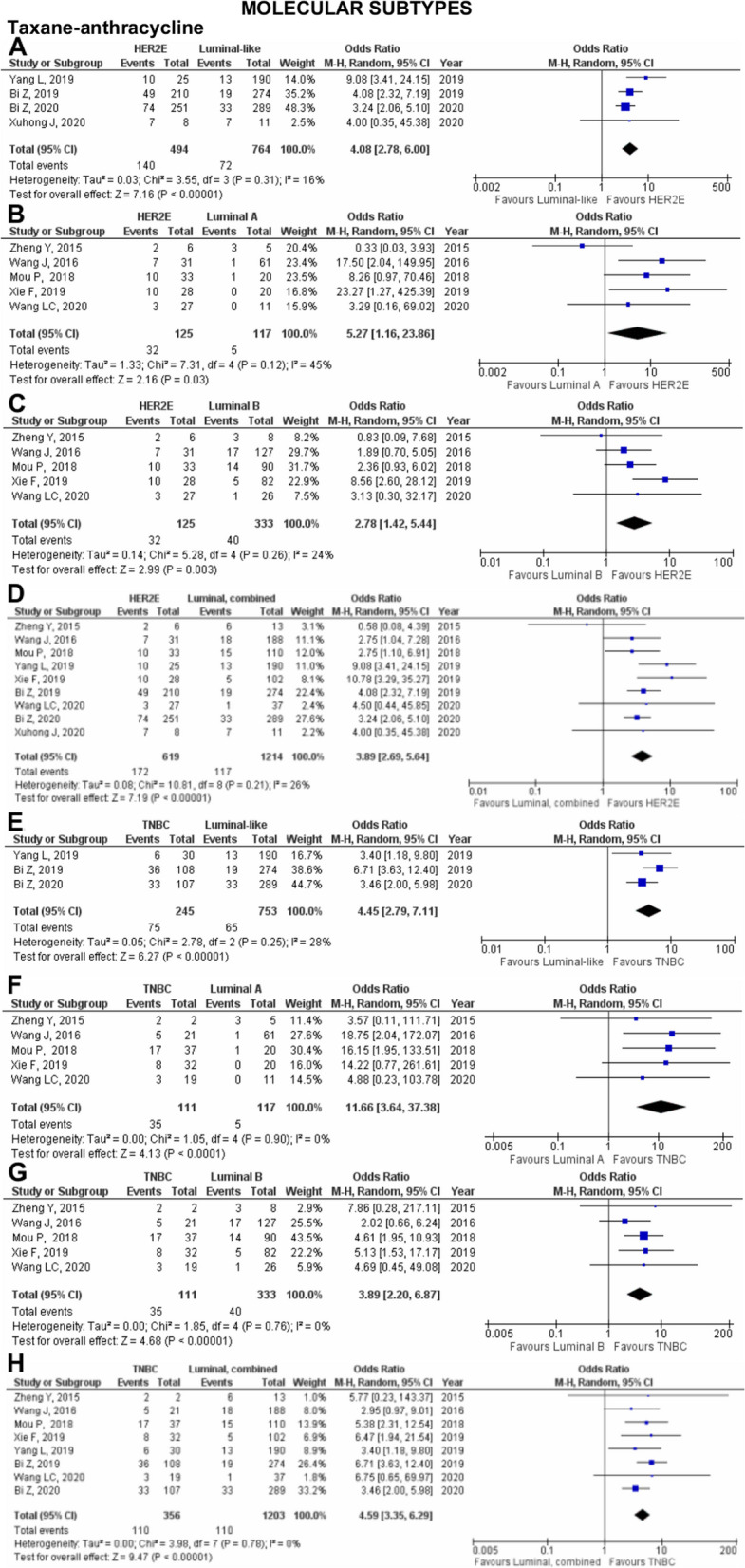
Pooled pCR outcome of TA-treated Asian breast cancer patients according to molecular subtypes. Forest plots describing the random effect ORs and 95% CIs from studies assessing the association of pCR outcome in NAC TA-treated breast cancer patients between (**A**) HER2E and luminal-like; (**B**) HER2E and luminal A; (**C**) HER2E and luminal B; (**D**) HER2E and luminal, combined; and (**E**) TNBC and luminal-like; (**F**) TNBC and luminal A; (**G**) TNBC and luminal B; and (**H**) TNBC and luminal, combined. *I*^2^ and *p*-value for *X*^2^ of heterogeneity are reported for each group analysis

**Fig. 3:**
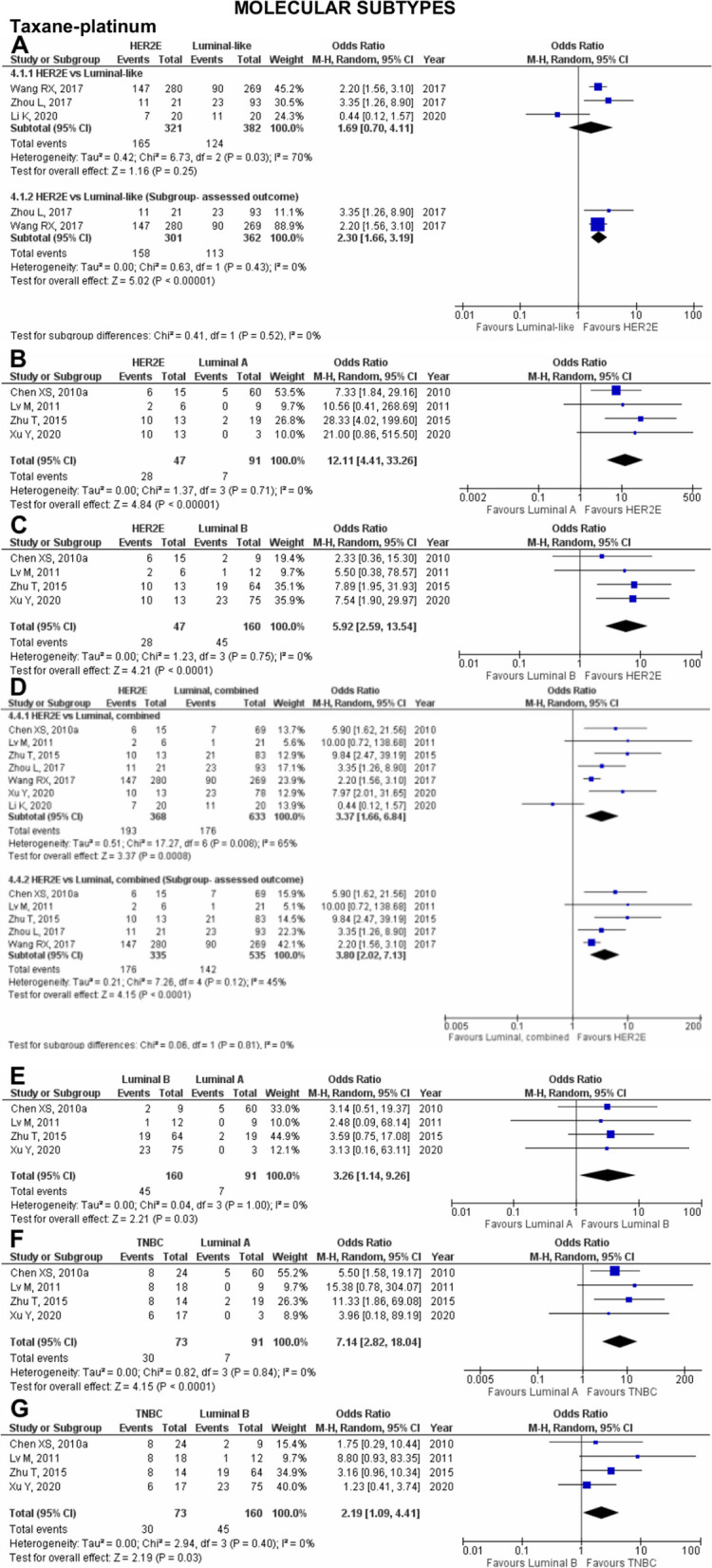
Pooled pCR outcome of TP-treated Asian breast cancer patients according to molecular subtypes. Forest plots describing the random effect ORs and 95% CIs from studies assessing the association of pCR outcome in NAC TP-treated Asian breast cancer patients between (**A**) HER2E and luminal-like; (**B**) HER2E and luminal A; (**C**) HER2E and luminal B; (**D**) HER2E and luminal, combined; (**E**) Luminal B and luminal A; (**F**) TNBC and luminal A; (**G**) TNBC and luminal B. *I*^2^ and *p*-value for *X*^2^ of heterogeneity are reported for each group analysis

**Fig. 4:**
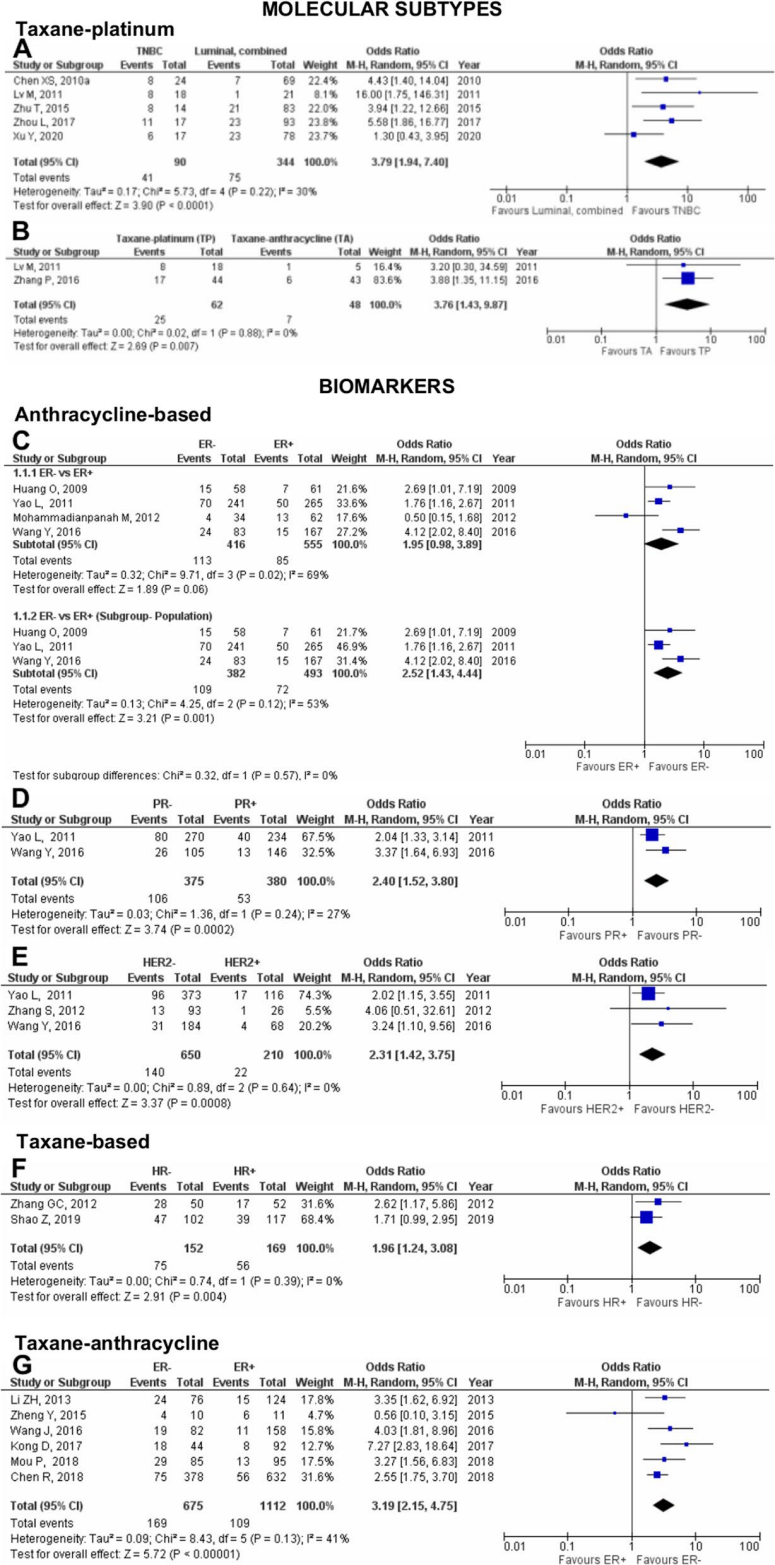
Pooled pCR outcome of NAC-treated Asian breast cancer patients according to molecular subtypes and biomarkers. Forest plots describing the random effect ORs and 95% CIs from studies assessing the association of pCR outcome in (**A**) NAC TP-treated Asian breast cancer patients between TNBC and luminal, combined; (**B**) between Asian TNBC patients treated with NAC TP and TA; Asian breast cancer patients treated with anthracycline-based chemotherapy with (**C**) ER; (**D**) PR; and (**E**) HER2 biomarkers; (**F**) Asian breast cancer patients treated with taxane-based chemotherapy and HR biomarker; (**G**) Asian breast cancer patients treated with TA and ER biomarker. *I*^2^ and *p*-value for *X*^2^ of heterogeneity are reported for each group analysis

**Fig. 5:**
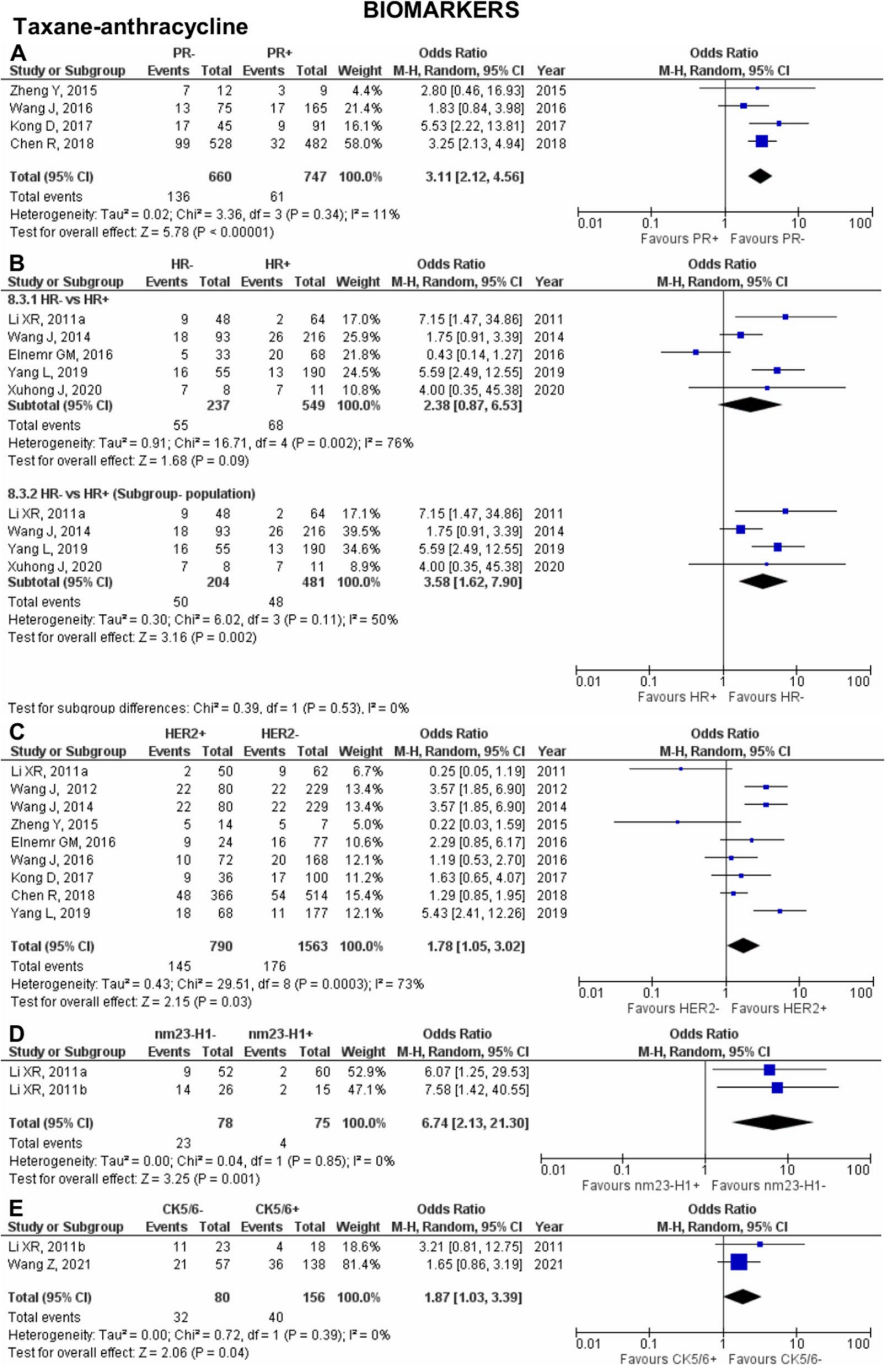
Pooled pCR outcome of TA-treated Asian breast cancer patients according to biomarkers. Forest plots describing the random effect ORs and 95% CIs from studies assessing the association of pCR outcome in NAC TA-treated breast cancer patients in biomarkers (**A**) PR; (**B**) HR; (**C**) HER2; (**D**) nm23-H1; and (**E**) CK5/6. *I*^2^ and *p*-value for *X*^2^ of heterogeneity are reported for each group analysis

**Fig. 6:**
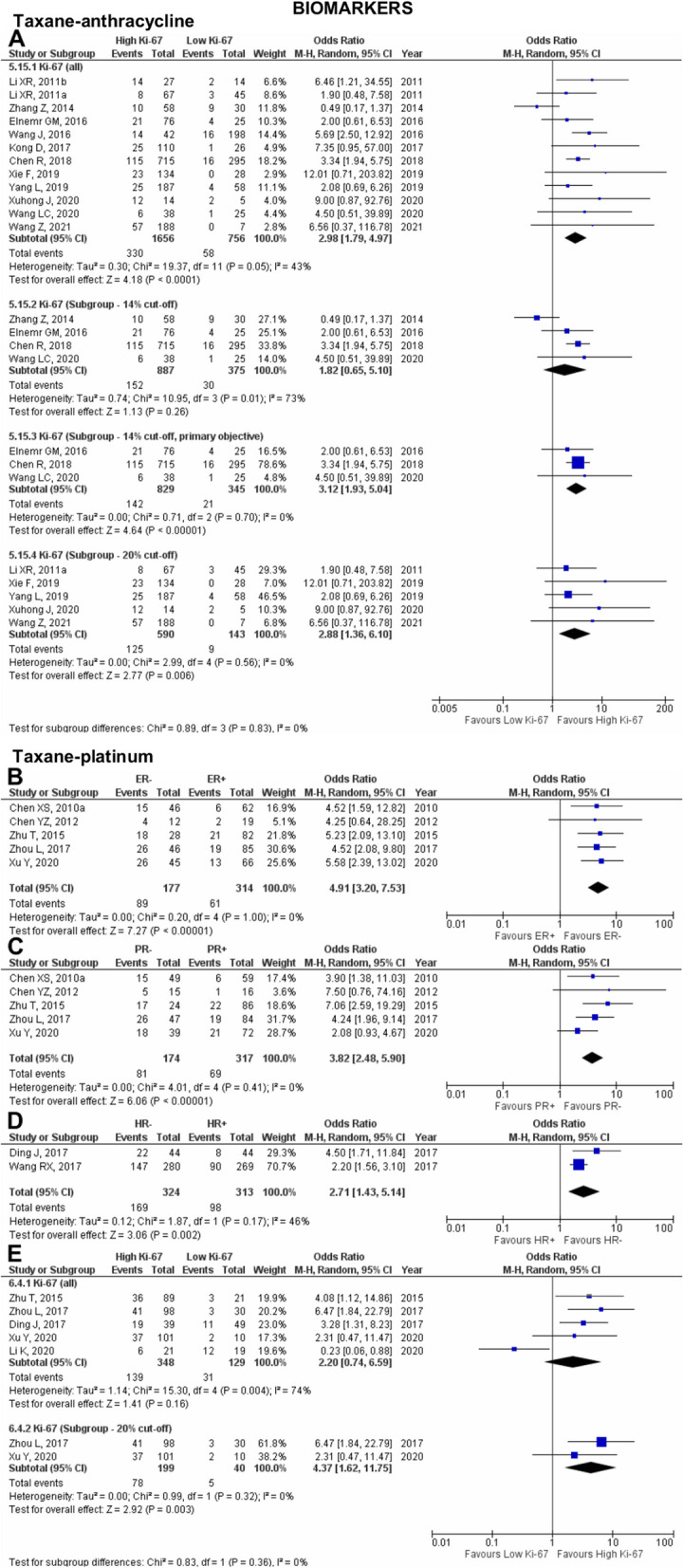
Pooled pCR outcome of NAC-treated Asian breast cancer patients according to biomarkers. Forest plots describing the random effect ORs and 95% CIs from studies assessing the association of pCR outcome in (**A**) NAC TA-treated breast cancer patients in biomarkers Ki-67; NAC TP-treated breast cancer patients in biomarkers (**B**) ER; (**C**) PR; (**D**) HR; and (**E**) Ki-67. *I*^2^ and *p*-value for *X*^2^ of heterogeneity are reported for each group analysis

**Fig. 7:**
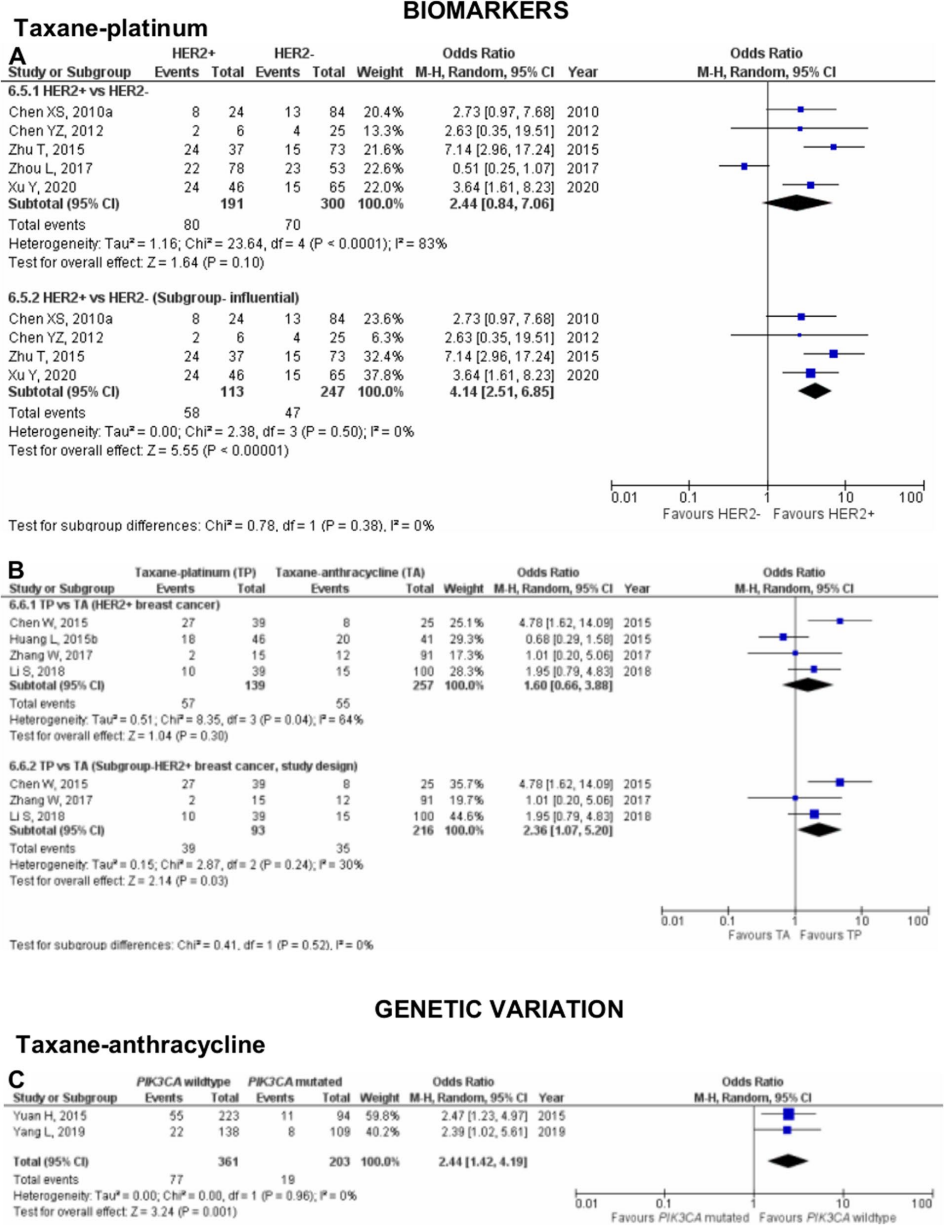
Pooled pCR outcome of NAC-treated Asian patients according to biomarkers and genetic variation. Forest plots describing the random effect ORs and 95% CIs from studies assessing the (**A**) Association of pCR outcome in NAC TP-treated Asian breast cancer patients in HER2; (**B**) Association between pCR in Asian patients with HER2+ biomarker treated with NAC TP and TA; and (**C**) Association of pCR outcome in NAC TA-treated Asian breast cancer patients in *PIK3CA* gene. *I*^2^ and *p*-value for *X*^2^ of heterogeneity are reported for each group analysis

The molecular subtypes classification of breast cancer, presence or absence of specific biomarkers, and genetic variations in the breast cancer diagnosis can be utilised to predict the pCR outcome in patients treated with specific chemotherapeutic agents. Five molecular subtypes, fourteen biomarkers, and eleven genetic variations were qualitatively evaluated for their predictive value in Asian breast cancer patients (Supplementary Table 4.2.2 in Additional file 4). Meanwhile, of the 101 studies, 60 studies provided data that could be used for meta-analysis (Figs. 2, 3, 4, 5, 6 and 7 in the manuscript and Supplementary Figures 7.1– 7.8 in Additional file 7). All the qualitative and meta-analyses results are presented by the molecular classification, biomarkers, and genetic characterisation of Asian breast cancer patients. Additionally, meta-analysis results using the Mantel-Haenszel method listed under each breast cancer characteristic are grouped according to the chemotherapeutic agents. Meta-analysis results using the inverse-variance method are presented separately since it is pooling the reported association data, for which they are presented by the breast cancer characteristics as well.

### Molecular subtype classification

Qualitatively, most studies with molecular subtype classification provide data on the pCR rates of patients treated with TA and TP regimens. When treated with TP, luminal A had the lowest pCR rate of 7.7% (*n*=4), while the highest pCR rate was observed in HER2E at 52.4% (*n*=7). In comparison, the pCR rates in the other subtypes were 32.5% (*n*=3), 28.1% (*n*=4), and 41.7% (*n*=7) for luminal-like, luminal B, and TNBC, respectively. However, when treated with NAC TA, the highest pCR rate was observed in TNBC at 30.4% (*n*=20), and similarly, the lowest pCR rate was observed in luminal A at 4.3% (*n*=5). Comparatively, the pCR rates in luminal-like, luminal B, and HER2E were 9% (*n*=6), 12% (*n*=5), and 27.8% (*n*=9), respectively. Our findings suggest that patients with TNBC and HER2E subtypes treated with NAC TP and TA were more likely to obtain pCR, while luminal A was less likely to obtain pCR with both regimens.

Under meta-analysis, the role of molecular subtypes was examined in NAC TA-treated (Figure 2 and Supplementary Figure 7.1 in Additional file 7) and TP-treated (Figures 3 and 4 and Supplementary Figure 7.1 in Additional file 7) breast cancer patients.

### Taxane-anthracycline (TA) chemotherapy

Our study first compares the effect of HER2E and luminal subtypes on pCR outcomes in TA-treated patients (Fig. 2A–D). In the analysis of four pooled studies [34, 35, 98, 100] comparing HER2E and luminal-like subtypes, 494 were identified with HER2E subtype and 764 patients with luminal-like subtype. Despite the large number of patients with luminal-like subtypes, our findings significantly associate patients with HER2E subtypes with better pCR outcomes (OR: 4.08; 95% CI; 2.78–6.00; *p*<0.0001; Fig. 2A). When HER2E patients were compared with patients with luminal A subtype, patients with HER2E subtypes were also found to be significantly more likely to achieve pCR (OR 5.27; 95% CI 1.16–23.86; *p*=0.03; Fig. 2B). Similarly, when HER2E was analysed against luminal B, HER2E was significantly associated with pCR (OR 2.78; 95% CI 1.42–5.44; *p*=0.003; Fig. 2C). To confirm and elucidate the effect of luminal subtypes on pCR outcome in NAC TA-treated breast cancer patients, we combined all luminal data (luminal-like, luminal A, and luminal B) as luminal, combined and compared it against HER2E patients. Nine studies [34, 35, 71, 80, 84, 95, 98, 100, 113] were pooled, yet our findings still showed that HER2E subtype was significantly associated with pCR outcome in NAC TA-treated patients (OR 3.89; 95% CI 2.69-5.64; *p*<0.0001; Fig. 2D).

Similarly, analyses conducted comparing the effect of TNBC and luminal subtypes on pCR outcome in TA-treated patients (Fig. 2E–H) significantly associate TNBC with better pCR outcome compared to luminal-like (OR 4.45; 95% CI 2.79–7.11; *p*<0.0001; Fig. 2E), luminal A (OR 11.66; 95% CI 3.64-37.38; *p*<0.0001; Fig. 2F), luminal B (OR 3.89; 95% CI 2.20–6.87; *p*<0.0001; Fig. 2G), and luminal, combined (OR 4.59; 95% CI 3.35–6.29; *p*<0.0001; Fig. 2H).

To further explore the effect of molecular subtypes on the pCR outcome of NAC TA-treated breast cancer patients, the TNBC subtype was compared to the HER2E subtype revealing that neither was associated with pCR (OR 1.17; 95% CI 0.80-1.70; *p*=0.43; Supplementary Figure 7.1A in Additional file 7). Similarly, an analysis between luminal A and luminal B showed that neither was associated with better pCR outcome when treated with NAC TA (OR 2.47; 95% CI 0.79–7.73; *p*=0.12; Supplementary Figure 7.1B in Additional file 7).

### Taxane-platinum (TP) chemotherapy

In the analysis pooling three studies [45,64,102] comparing HER2E and luminal-like subtypes, 321 patients were identified with HER2E subtype, and 382 patients were luminal-like. When treated with NAC TP, neither HER2E nor luminal-like patients were associated with pCR (OR 1.69; 95% CI 0.70–4.11; *p*=0.25; Fig. 3A). However, in the analysis pooling four studies [41, 69, 97, 115] comparing HER2E and luminal A subtypes, HER2E was significantly associated with pCR outcome (OR 12.11; 95% CI 4.41–33.26; *p*<0.0001; Fig. 3B). Similarly, when comparing against luminal B and luminal, combined, HER2E was significantly associated with pCR outcome (OR 5.92; 95% CI 2.59–13.54; *p*<0.0001; Fig. 3C and OR 3.37; 95% CI 1.66–6.84; *p*=0.0008; Fig. 3D, respectively). Substantial heterogeneity was reported for two of the pooled analyses: (1) in studies comparing HER2E and luminal-like (Fig. 3A); and (2) in studies comparing HER2E and luminal, combined (Fig. 3D). Although all three and seven studies pooled in the two analyses were performed in the Chinese population, the primary outcome assessed in Li et al. [57] and Xu et al. [97] focused on the contribution of genetic mutations or long non-coding RNAs (lncRNAs) as a predictor of pCR status in the recruited population. Consequently, pooled analysis excluding Li et al. eliminates the heterogeneity in the first analysis revealing significant association (OR 2.30; 95% CI 1.66–3.19; *p*<0.00001), while pooled analysis excluding Li et al. and Xu et al. in the second analysis decreased the heterogeneity, and maintaining the significant association (OR 3.80; 95% CI 2.02–7.13; *p*<0.0001).

Notably, when HER2E and TNBC patients were compared, neither was associated with pCR (OR 1.46; 95% CI 0.63–3.37; *p*=0.38; Supplementary Figure 7.1C in Additional file 7). We then compared luminal B and luminal A patients and found that luminal B patients were significantly associated with better pCR outcomes (OR 3.26; 95% CI 1.14–9.26; *p*=0.03; Fig. 3E). Similar to NAC TA-treated patients, analyses conducted comparing the effect of TNBC and luminal subtypes on pCR outcome in TP-treated patients also significantly associate TNBC with better pCR outcome compared to luminal A (OR 7.14; 95% CI 2.82–18.04; *p*<0.0001; Fig. 3F), luminal B (OR 2.19; 95% CI 1.09–4.41; *p*=0.03; Fig. 3G), and luminal, combined (OR 3.79; 95% CI 1.94–7.40; *p*<0.0001; Fig. 4A).

Our study compared the effect of chemotherapeutic agents on the pCR outcome in TNBC patients (Fig. 4B). Pooled analysis involving two studies [69, 108] showed that TNBC patients were significantly more likely to achieve pCR when treated with NAC TP (*n*=25/62) compared to NAC TA (*n*=7/48) (OR 3.76; 95% CI 1.43-9.87; *p*=0.007).

### Biomarkers

Qualitatively, most studies with biomarkers data comprise of routinely analysed biomarkers—ER, PR, HER2, and Ki-67. In ER− and ER+ patients, anthracycline-based chemotherapy showed pCRrate of 27.2% (*n*=4) vs 15.3% (*n*=4). Meanwhile, TP, TA, and TAP chemotherapy showed pCR rate of 48.1% (*n*=6) vs 19.4% (*n*=5), 25.0% (*n*=6) vs 9.0% (*n*=7), and 29.7% (n=1) vs 12.9% (*n*=1), respectively. As for PR− and PR+ patients, anthracycline-based, TP, TA, and TAP chemotherapy showed pCR rate of 28.3% (*n*=2) vs 13.9% (*n*=2), 46.6% (*n*=5) vs 21.8% (*n*=5), 20.6% (*n*=4) vs 8.2% (*n*=4), and 25% (*n*=1) vs 13.3% (*n*=1), respectively. Collectively*,* ER− and PR− breast cancer patients were likely to benefit more from TP regimen than TA, TAP, and anthracycline-based chemotherapy. Notably, some studies combined their report of ER and PR as hormone receptors (HR). Analysis of HR+ and HR− patients showed pCR rate of 11.9% (*n*=1) vs 10% (*n*=1), 33.1% (*n*=2) vs 49.3% (*n*=2), 31.3% (*n*=2) vs 50.7% (*n*=3), and 12.4% (*n*=5) vs 23.2% (*n*=5) when treated with anthracycline-based, taxane-based, TP, and TA chemotherapy, respectively. Our findings suggested that HR+ breast cancer patients achieved a better pCR rate when treated with a single-based chemotherapeutic agent, while HR− patients benefit more combination chemotherapy regimens.

In HER2+ and HER2− patients, anthracycline-based chemotherapy showed pCR rate of 10.5% (*n*=3) vs 19.3% (*n*=4). Meanwhile, TP, TA, and TAP chemotherapy showed pCR rate of 44.6% (*n*=15) vs 24.3% (*n*=6), 20.1% (*n*=14) vs 11.3% (*n*=12) and 33% (*n*=1) vs 13.5% (*n*=1), respectively. One study [44] utilising CDK4/6 inhibitor on ER+/HER2− breast cancer patients and another study [118] utilising kinase inhibitor on ER+/HER2+ patients showed pCR rate of 5% (*n*=1) and 0% (*n*=1), respectively. Collectively, with the exception of anthracycline-based and targeted therapy, HER2+ breast cancer patients were likely to benefit more from TP, TA, and TAP regimens. Meanwhile, in patients with high and low Ki-67, anthracycline-based and taxane-based chemotherapy showed pCR rate of 12.2% (*n*=1) vs 11.4% (*n*=1) and 57.1% (*n*=1) vs 32.1% (*n*=1). When treated with TP and TA chemotherapy, the patients showed pCR rate of 39.9% (*n*=5) vs 24% (*n*=5) and 19.9% (*n*=12) vs 7.7% (*n*=12), respectively. Overall, breast cancer patients with high Ki-67 were likely to benefit more from taxane-based chemotherapy than TP, TA, and anthracycline-based regimen.

On the other hand, fourteen non-conventional biomarkers investigated in a few of the included studies were evaluated qualitatively. Three biomarkers—Bcl-2, Smac, and Survivin—were included for the evaluation of anthracycline-based chemotherapy. Anthracycline-based chemotherapy showed pCR benefit of 26.1% (*n*=1) vs 4.3% (*n*=1) in Bcl-2- and Bcl2+ breast cancer patients, 35.0% (*n*=1) vs 8.6% (*n*=1) in high and low Smac, and 28.3% (*n*=1) vs 11.5% (*n*=1) in low and high Survivin. Only one biomarker—ZEB1—was evaluated for TP chemotherapy which revealed pCR rates of 36.1% (*n*=1) in patients with low ZEB1 and 12.8% (*n*=1) in patients with high ZEB1.

Ten biomarkers—Tau, P-gp, Topo-II, T-cadherin, CK5/6, EGFR, p53, LAG-3, cyclin D1, and nm23-H1—were included for the evaluation of TA chemotherapy. Our findings showed pCR benefit of 31.3% (*n*=1) vs 4.5% (*n*=1) in Tau− and Tau+, 43.2% (*n*=1) vs 7.7% (*n*=1) in P-gp− and P-gp+, and 17% (*n*=1) vs 3.4% (*n*=1) in Topo-II- and Topo-II+ breast cancer patients. The pCR rates observed in T-cadherin- and T-cadherin+, CK5/6− and CK5/6+, EGFR− and EGFR+, and p53− and p53+ breast cancer patients were 45.2% (*n*=1) vs 7.4% (*n*=1), 40% (*n*=2) vs 25.6% (*n*=2), 45.5% (*n*=1) vs 28.1% (*n*=1), and 33.3% (*n*=1) vs 27.3% (*n*=1), respectively. As for breast cancer patients with low and high expression of LAG-3, the pCR rates was observed at 64.7% (*n*=2) vs 35.3% (*n*=2). Lastly, breast cancer patients with cyclin D1+ and nm23-H1+ reported pCR benefits of 45.8% (*n*=1) and 29.5% (*n*=2) than 29.4% (*n*=1) in cyclin D1− and 5.3% (*n*=2) in nm23-H1− breast cancer patients.

Under meta-analysis, the role of biomarkers was investigated in NAC anthracycline-based and taxane-based treated (Fig. 4), TA-treated (Figs. 4, 5 and 6 and Supplementary Figure 7.1 in Additional file 7), and TP-treated (Figs. 6 and 7) breast cancer patients.

### Anthracycline-based and taxane-based chemotherapy

Four studies [53, 88, 102, 124] were pooled for the effect of ER on pCR outcome in anthracycline-treated patients, where ER was not associated with pCR (OR 1.95; 95% CI 0.98–3.89; *p*=0.06; Fig. 4C). Substantial heterogeneity was reported for the pooled analysis of ER− vs ER+, which could be explained by the study by Mohammadianpanah et al. [124] which was conducted in the Iranian population. In contrast, the other studies [53, 88, 102] were performed in the Chinese population. Subgroup analysis of ER− vs ER+ pooling studies in the Chinese population only showed that ER− patients were significantly associated with pCR (OR 2.52; 95% CI 1.43–4.44; *p*=0.001; Fig. 4C), supporting our hypothesis that the observed heterogeneity could be due to the difference in the Asian population. Notably, the observed moderate heterogeneity in the subgroup analysis can allude to by the differences in the population sizes of the three studies. Only two studies [88, 102] were pooled to analyse PR effect on pCR outcome in anthracycline-treated patients. Our findings showed that PR− patients were significantly associated with pCR (OR 2.40; 95% CI 1.52–3.80; *p*=0.0002; Fig. 4D). Meanwhile, an analysis of three pooled studies [88, 102, 109] on the effect of HER2 on pCR outcome in anthracycline-treated breast cancer patients revealed that patients with HER2− biomarker were significantly more likely to achieve pCR (OR 2.31; 95% CI 1.42–3.75; *p*=0.0008; Fig. 4E). Only one biomarker, HR, was evaluated for its effect on pCR outcome in Asian breast cancer patients treated with taxane-based chemotherapy (Fig. 4F). Two studies [74, 107] were pooled where patients with HR− biomarkers were significantly more likely to achieve pCR than HR− patients (OR 1.96; 95% CI 1.24–3.08; *p*=0.004).

### Taxane-anthracycline (TA) chemotherapy

Eight biomarkers comprising ER, PR, HR, HER2, nm23-H1, CK5/6, EGFR, and Ki-67 were investigated for their effect on pCR outcome in Asian breast cancer patients treated with NAC TA. Six studies [45, 49, 52, 77, 81, 112] and four studies [36, 54, 80, 113] were pooled to evaluate the association of ER and PR, respectively. Both ER− (OR 3.19; 95% CI 2.15–4.75; *p*<0.0001; Fig. 4G) and PR− (OR 3.11; 95% CI 2.12-4.56; *p*<0.0001; Fig. 5A) were significantly associated with pCR outcome in TA-treated patients. Moderate heterogeneity was reported for the pooled analysis of ER− vs ER+. However, considering all the studies pooled for the analysis were performed in the Chinese population, the heterogeneity result was rejected.

In an analysis pooling five studies [60, 81, 98, 100, 127], HR was not associated with pCR outcome (OR 2.38; 95% CI 0.87–6.53; *p*=0.09; Fig. 5B). The observed substantial heterogeneity could be due to the study by Elnemr et al. [127] conducted in Saudi Arabia, while the other four studies were performed in China. Consequently, subgroup analysis of HR− vs HR+ removing the study by Elnemr et al. showed a decrease in the heterogeneity and significantly associated HR− with better pCR outcome when treated with TA (OR 3.58; 95% CI 1.62–7.90; *p*=0.002). An analysis of nine pooled studies [36, 54, 60, 80–82, 100, 113, 127] showed that HER2+ is significantly associated with pCR (OR 1.78; 95% CI 1.05–3.02; *p*=0.03; Fig. 5C) with substantial heterogeneity observed between the studies. Although the analysis also includes the study by Elnemr et al., which was conducted in Saudi Arabia, the heterogeneity could be influenced by the results pooled from seven studies that heavily pushed the effect of our analysis in one direction.

Our study synthesised meta-analysis data for other biomarkers apart from the commonly reported ones—ER, PR, HR, and HER2. In particular, two studies were evaluated for pCR outcome for nm23-H1 [12, 60] and CK5/6 [12, 90]. It was observed that nm23-H1− (OR 6.74; 95% CI 2.13–21.30; *p*=0.001; Fig. 5D) and CK5/6– (OR 1.87; 95% CI 1.03–3.39; *p*=0.04; Fig. 5E) are significantly associated with pCR. Two studies [12, 90] were pooled for analysis in the evaluation of pCR outcome with EGFR. Considerable heterogeneity was observed between the studies, perhaps alluded to the clinical differences between the studies of Li et al. [12] (*n*=22/41) and Wang et al. [90] (*n*=170/195) according to the distribution of patients with EGFR+. Despite that, EGFR is not associated with pCR outcome in TA-treated patients (OR 2.02; 95% CI 0.28–28.00; *p*=0.38; Supplementary Figure 7.1D in Additional file 7).

The proliferation index biomarker, Ki-67, was evaluated through an analysis of 12 pooled studies [12, 36, 54, 60, 80, 84, 90, 95, 98, 100, 111, 127], revealing significant association between pCR outcome and high Ki-67 (OR 2.98; 95% CI 1.79–4.97; *p*<0.0001; Fig. 6A). The observed moderate heterogeneity between the studies could be due to differences in the Ki-67 cut-off value. Subgroup analysis pooling four studies [36, 84, 111, 127] with 14% Ki-67 cut-off did not significantly associate Ki-67 with pCR outcome (OR 1.82; 95% CI 0.65–5.10; *p*=0.26). Significant heterogeneity was observed which could be explained by the primary research question addressed in the studies where Zhang et al. [111] focused on the prognostic value of magnetic resonance imaging (MRI), P-gp, and Ki-67, while the other three studies focused on the correlation of Ki-67 expression and pCR. Furthermore, the results pooled from the three studies heavily pushed the effect of our analysis in one direction. Thus, a pooled analysis excluding the study by Zhang et al. reveals null heterogeneity between the studies and a significant association between pCR and high Ki-67 with 14% cut-off value (OR 3.12; 95% CI 1.93–5.04; *p*<0.00001). Meanwhile, subgroup analysis pooling five studies [60, 90, 95, 98, 100] with 20% Ki-67 cut-off significantly associate pCR outcome with high Ki-67 (OR 2.88; 95% CI 1.36–6.10; *p*=0.006).

### Taxane-platinum (TP) chemotherapy

Our study investigated five biomarkers comprising ER, PR, HR, HER2, and Ki-67 on their effect on pCR outcome in Asian breast cancer patients treated with NAC TP. Five studies [41, 43, 97, 114, 115] and two studies [100,102] were pooled to evaluate the association of ER and PR, and HR, respectively. pCR outcome was significantly associated with ER− (OR 4.91; 95% CI 3.20–7.53; *p*<0.00001; Fig. 6B), PR− (OR 3.82; 95% CI 2.48–5.90; *p*<0.00001; Fig. 6C), and HR− (OR 2.71; 95% CI 1.43–5.15; *p*=0.002; Fig. 6D).

Ki-67 was evaluated by analysing five pooled studies [47, 57, 97, 114, 115], showing that pCR outcome was not significantly associated with either high Ki-67 or low Ki-67 (OR 2.20; 95% CI 0.74–6.59; *p*=0.16; Fig. 6E). The observed considerable heterogeneity was perhaps due to differences in the Ki-67 cut-off value, and four of the five studies heavily pushed the effect of our analysis to one direction. Subgroup analysis pooling two studies [97, 114] with 20% Ki-67 cut-off indicates that pCR outcome is significantly associated with high Ki-67 (OR 4.37; 95% CI 1.62–11.75; *p*=0.003). This implicates the importance of having a standardised cut-off value for Ki-67, as at different cut-offs of 15 and 30%, neither Ki-67 biomarker was favoured as opposed to the 20% cut-off favours High Ki-67 to achieve pCR.

In the analysis of the effect of HER2 in breast cancer patients pooling five studies [41, 43, 97, 114, 115], pCR outcome was not significantly associated with neither HER2+ nor HER2− (OR 2.44; 95% CI 0.84–7.06; *p*=0.10; Fig. 7A). The observed substantial heterogeneity could be influenced by the results pooled from four of the five studies that heavily pushed the effect of our analysis in one direction. Consequently, a pooled analysis excluding the study by Zhou et al. [114] reveals null heterogeneity between the studies and a significant association between pCR and HER2+ (OR 4.14; 95% CI 2.51–6.85; *p*<0.00001).

Our study also evaluated the effect of chemotherapeutic agents on the pCR outcome in Asian breast cancer patients with HER2+ biomarker (Fig. 7B). Four studies [39, 51, 59, 110] were pooled and estimated. Our findings revealed neither NAC TP nor NAC TA was associated with pCR outcome in patients with HER2+ biomarker (OR 1.60; 95% CI 0.66–7.06; *p*=0.30). Substantial heterogeneity was reported which could be explained by the difference in the study design of the pooled studies. Of the four studies, Huang et al. [51] was the only study that conducted a randomised controlled trial (RCT) where the recruited HER2+ breast cancer patients were assigned to either TA or TP chemotherapy by the investigator. In contrast, HER2+ breast cancer patients recruited in the other three studies [39, 59, 110] were given either TA or TP regimen based on their preferences. Notably, subgroup analysis excluding Huang et al. decreased the heterogeneity *I*^2^ and significantly associated HER2+ breast cancer patients with better pCR outcome when treated with TP (OR 2.36; 95% CI 1.07–5.20; *p*=0.03).

### Genetic variations and differential expression

Eleven genes—*PIK3CA, TP53, EPIC1, TOP2A, ERBB2, MYC, CCND1, PCDH17, EPIC1, BRCA1,* and *BRCA2*—were included for the qualitative evaluation of NAC regimens. No specific single variant vs wildtype was compared for most of the genes since most of the evaluated studies did not report them. Therefore, our analysis only compared wildtype (wt) and mutated (mt), where the mutated gene might contain single or multiple variants.

Breast cancer patient harbouring wt and mutated mt*PIK3CA* showed pCR rate of 19.4% (*n*=1) vs 14.1% (*n*=1) and 18.8% (*n*=1) vs 16.1% (*n*=1) when treated with anthracycline-based and taxane-based chemotherapy, respectively. Meanwhile, patients treated with TA chemotherapy showed a pCR rate of 21.3% (*n*=2) vs 9.4% (*n*=2). Thus, breast cancer patients with wt*PIK3CA* were likely to benefit more from TA regimen than anthracycline-based and taxane-based chemotherapy. Interestingly, breast cancer patient harbouring wt and mt*TP53* showed pCR rate of 7.1% (*n*=1) vs 28.6% (*n*=1), 11.3% (*n*=1) vs 15.2% (*n*=1), and 6.1% (*n*=1) vs 16.1% (*n*=1) when treated with anthracycline-based, taxane-based, and TA chemotherapy, respectively. Our findings suggested that breast cancer patients with mt*TP53* were likely to benefit more from anthracycline-based chemotherapy than taxane-based and TA regimen.

Our findings also showed that breast cancer patients with *TOP2A*, *ERBB2*, and *MYC* amplification (amp) achieved higher pCR rates than wt*TOP2A*, *ERBB2*, and *MYC* (56.3% (*n*=1) vs 13.8% (*n*=1), 28.4% (*n*=1) vs 6.1% (*n*=1), and 13.7% (*n*=1) vs 11.2% (*n*=1), respectively) when treated with TA regimens. On another note, breast cancer patients with wt*CCND1* and unmethylated (unm) *PCDH17* achieved higher pCR rate than those with *CCND1* amp andmethylated (m) *PCDH17* (13.8% (*n*=1) vs 2.7% (*n*=1) and 67.3% (*n*=1) vs 31.6% (*n*=1), respectively)*.*

One included study by Mou et al. [71] focused on the effect of *UGT2B7* rs7435335 on NAC TA efficacy. It was observed that patients with the genotype GA achieved higher pCR rate (42.3% (*n*=1)) than patients with the genotype GG (18.9% (*n*=1)). Another study by Xu et al. [96] analysed the effect of *BRCA1* and *BRCA2* mRNA expression in breast cancer patients treated with anthracycline-based and taxane-based chemotherapy. Our findings showed pCR benefit of 24.6% (*n*=1) vs 16.9% (*n*=1), 16.9% (*n*=1) vs 17.5% (*n*=1), and 14% (*n*=1) vs 20.8% (*n*=1) in anthracycline-based treated patients with low, intermediate, and high *BRCA1* vs *BRCA2* mRNA expression, respectively. Meanwhile, in taxane-based treated patients, our findings showed pCR benefit of 19.6% (*n*=1) vs 24.4% (*n*=1), 26.8% (*n*=1) vs 23.4% (*n*=1), and 21.4% (*n*=1) vs 18.9% (*n*=1) with low, intermediate, and high *BRCA1* vs *BRCA2* mRNA expression, respectively. Notably, breast cancer patients with low *EPIC1* showed higher pCR rate (40.7% (*n*=1)) when treated with TP regimen than patients with high *EPIC1* (33.3% (*n*=1)).

Under meta-analysis, only one gene was analysed for its effect on pCR outcome in Asian breast cancer patients treated with NAC TA (Fig. 7).

### Taxane-anthracycline (TA) chemotherapy

From the analysis of two studies [100, 106], 564 patients were pooled for *PIK3CA* analysis. It was observed that patients harbouring wt*PIK3CA* were significantly associated with better pCRoutcomes compared to patients with mt*PIK3CA* gene (OR: 2.44; 95% CI 1.42–4.19; *p*=0.001; Fig. 7).

The overall summary results of pooled pCR outcome of NAC-treated Asian breast cancer patients of this study can be found in Table 1 whereby the favoured outcome for molecular subtypes with various NAC treatments were HER2E, TNBC, and Luminal B. The favoured outcome for biomarkers across different NAC treatments was ER−, PR−, HR− and high ki67. Lastly, the favoured outcome for genetic variation was the *PIK3CA* wildtype. The overall summary of the steps conducted in completing this systematic review is presented in Table 2.

**Table 1:** Pooled pCR outcome of NAC-treated Asian breast cancer patients according to molecular subtypes, biomarkers and genetic variations

Treatments	Variables (molecular subtypes, biomarker, genetic variation)				
	**Molecular subtypes**	OR	95%CI	Overall *P*-value	Favoured outcome
Taxane-Anthracycline	Luminal-like vs HER2E	4.08	2.78,6.00	*P*< 0.00001	HER2E
	Luminal A vs HER2E	5.27	1.16, 23.86	*P*=0.03	HER2E
	Luminal B vs HER2E	2.78	1.42,5.44	*P*=0.0003	HER2E
	Luminal, combined vs HER2E	3.89	2.69,5.64	*P*< 0.00001	HER2E
	TNBC vs Luminal-like	4.45	2.79,7.11	*P*< 0.0001	TNBC
	TNBC vs Luminal A	11.66	3.64, 37.38	*P*< 0.00001	TNBC
	TNBC vs Luminal B	3.89	2.20, 6.87	*P*< 0.00001	TNBC
	TNBC vs Luminal, combined	4.59	3.35, 6.29	*P*< 0.00001	TNBC
Taxane-platinum	Luminal-like vs HER2E (the subgroup-assessed outcome)	2.30	1.66,319	*P*< 0.00001	HER2E
	Luminal A vs HER2E	12.11	4.41,33.26	*P*< 0.00001	HER2E
	Luminal B vs HER2E	5.92	2.59,13.54	*P*< 0.0001	HER2E
	Luminal, combined vs HER2E	3.37	1.66, 6.84	*P*= 0.0008	HER2E
	Luminal, combined vs HER2E (subgroup- assessed outcome)	3.80	2.02,7.13	*P*< 0.0001	HER2E
	Luminal B vs Luminal A	3.26	1.14, 9.26	*P*=0.03	Luminal B
	TNBC vs Luminal A	7.14	2.82,18.04	*P*< 0.0001	TNBC
	TNBC vs Luminal B	2.19	1.09,4.41	*P*=0.03	TNBC
	TNBC vs Luminal-like	3.79	1.94, 7.40	*P*< 0.0001	TNBC
Anthracycline-based	**Biomarkers**				
	ER− vs ER+	1.95	0.98,3.89	*P*=0.06	ER−
	ER− vs ER+ (subgroup-population)	2.52	1.43, 4.44	*P*=0.001	ER−
	PR− vs PR+	2.40	1.52, 3.80	*P*=0.0002	PR−
	HER2− vs HER2+	2.31	1.42,3.75	*P*=0.0008	HER2−
Taxane-based	HR− vs HR+	1.96	1.24, 3.08	*P*=0.004	HR−
Taxane-anthracycline	ER− vs ER+	3.19	2.15, 4.75	*P*< 0.00001	ER−
	PR− vs PR+	3.11	2.12, 4.56	*P*< 0.00001	PR−
	HR− vs HR+	2.38	0.87,6.53	*P*=0.09	HR−
	HR− vs HR+ (Subgroup-population)	3.58	1.62,7.90	*P*=0.002	HR−
	HER2− vs HER2+	1.78	1.05,3.02	*P*=0.0008	HER2+
	nm23-H1− vs nm23-H1+	6.74	2.13,21.30	*P*=0.001	nm23-H1−
	CK5/6− vs CK5/6+	1.87	1.03,3.39	*P*=0.04	CK5/6−
	High Ki67 vs low Ki67	2.98	1.79,4.97	*P*< 0.0001	high Ki67
	High Ki67 vs low Ki67 (subgroup- 14% cut-off)	1.82	0.65,5.10	*P*=0.20	high Ki67
	High Ki67 vs low Ki67 (subgroup- 14% cut-off, primary objective)	3.12	1.93,5.04	*P*< 0.00001	high Ki67
	High Ki67 vs low Ki67 (Subgroup- 20% cut-off)	2.88	1.36,6.10	*P*=0.006	high Ki67
Taxane-platinum	ER− vs ER+	4.91	3.20,7.53	*P*< 0.00001	ER−
	PR− vs PR+	3.82	2.45,5.90	*P*< 0.00001	PR−
	HR− vs HR+	2.71	1.43,5.41	*P*< 0.00001	HR−
	High Ki67 vs low Ki67	2.20	0.74,6.59	*P*=0.16	high Ki67
	High Ki67 vs low Ki67 (Subgroup- 20% cut-off)	4.37	1.62,11.75	*P*=0.003	high Ki67
	HER2− vs HER2+	2.44	0.84,7.06	*P*< 0.0001	HER2+
	HER2− vs HER2+ (Subgroup-influential)	4.14	2.51,6.85	*P*< 0.00001	HER2+
TP vs TA	In HER2+ breast cancer	1.60	0.66,3.88	*P*=0.30	TP
	Subgroup- HER2+ breast cancer study	2.36	1.07,5.26	*P*=0.03	TP
Taxane-anthracycline	**Genetic variation**				
	*PIK3CA* wildtype vs *PIK3CA* mutated	2.44	1.42,4.19	*P*=0.001	*PIK3CA* wildtype

**Table 2:** Steps conducted in the systematic review

Steps	Details
Research question	In Asian breast cancer patients, how does personalised and precision medicine (in terms of breast cancer molecular subtypes diagnosis, presence or absence of biomarkers and genetic variants affect breast cancer treatment response and outcome?
Inclusion criteria	Randomised trials, observational studies, case-control studies, and cohort studies of Asian breast cancer aged ≥18 years, who underwent systemic neoadjuvant chemotherapy treatment reporting the involvement of somatic genetic polymorphisms or biomarkers or molecular subtype classification on breast cancer treatment response. The studies are written in the English language.
Participants	Asian breast cancer patients
Outcome	*Outcome 1:* Treatment response *Outcome 2:* Survival
Search strategy	*Databases:* MEDLINE (PubMed), Science Direct, Scopus, and Cochrane Library. *Date range:* 01.01.2000 to 31.03.2021. *Search terms:* The search term strategies can be found in Additional file 2. The terms were adapted for different databases utilising a combination of Medical Subject Heading (MeSH) and keywords that are relevant which can be found in the titles and abstract.
Critical appraisal	The authors extracted data from the published reports independently. Disagreements were resolved by a third person. The Newcastle-Ottawa Scale (NOS) was used.
Data collection and synthesis	For each study, the extracted parameters include the article information (article title, first author, year published, journal published, country, and year of recruitment), study design, study population and sample size, characteristics of patients in three variables (molecular subtypes, biomarkers, and genetic variations), and the pCR data in selected variables. Notably, in the absence of molecular subtype classification in the included studies, whenever possible, they were approximated through the available biomarkers detected through IHC data. Data analysis was done using Review Manager Software (RevMan version 5.4.1) [30]. The odds ratio (OR), hazards ratio (HR), and their corresponding 95% confidence interval (95% CI) were assessed to evaluate the association between treatment response (pCR) and NAC treatment provided to breast cancer patients based on their molecular subtypes, biomarkers, and genetic variations. The strength of associations was estimated by calculating pooled ORs/HRs and 95% CIs, by which significance was stated using the *p*-value. A *p*-value <0.05 was considered statistically significant.
Process	Search (*n*=5746) Excluded with reasons [refer to Figure 1 for detailed reasons] (*n*=5610) Excluded after reviewing full-text (*n*=35) Included studies (*n*=101)
Results	Where statistically appropriate, studies were pooled. *Molecular subtypes:* Meta-analysis demonstrated that when treated with taxane-anthracycline, Asian breast cancer patients diagnosed with HER2E or TNBC achieved better pCR compared to those who are diagnosed with Luminal breast cancer. Meanwhile, when treated with taxane-platinum, HER2E and TNBC Asian breast cancer patients achieved better pCR compared to those who are diagnosed with Luminal breast cancer. When compared with Luminal A breast cancer patients, Luminal B Asian breast cancer patients achieved better pCR when treated with taxane-platinum. *Biomarkers:* Meta-analysis demonstrated that when treated with anthracycline-based treatment, ER−, PR− and HER2− Asian breast cancer patients achieved better pCR compared to those who are diagnosed with ER+, PR+ and HER2+ breast cancer. HR− Asian breast cancer patients are also demonstrated to respond better to taxane-based treatment. For Asian breast cancer patients treated with taxane-anthracycline, it was found that those who are diagnosed with ER−, PR−, HR−, HER2+, nm23-H1−, CK5/6− and high Ki67 biomarkers responded better to the treatment. As for Asian breast cancer patients treated with taxane-platinum, it was found that those who are diagnosed with ER−, PR−, HR−, HER2+ and high Ki67 biomarkers responded better to the treatment. *Genetic variation:* Meta-analysis also demonstrated that when treated with taxane-anthracycline-based treatment, Asian breast cancer patients who had wildtype *PIK3CA* gene achieved better pCR compared to those who were with mutated *PIK3CA* gene.

### Pooled reported association

Meta-analyses of pooled reported association of pCR were evaluated according to molecular classification, genetic variations, and biomarkers characterisation of the Asian breast cancer patients.

### Molecular classification

An adjusted pooled analysis of TNBC against non-TNBC patients showed that TNBC patients were significantly associated with better response when treated with neoadjuvant chemotherapy (OR 3.02; 95% CI 1.54–5.95; *p*=0.001; Supplementary Figure 7.2A in Additional file 7). The observed moderate heterogeneity could be due to the study by Lv et al. [69] that did not specifically study TNBC vs non-TNBC patients. Moreover, the recruited patients were either treated with anthracycline-based or TP or TA regimens. Meanwhile, Wu et al. [91] specifically recruited TNBC and non-TNBC patients, and all were treated with TA. Hence, Wu et al.’s result carried more weight than that of Lv et al. in this analysis. However, Lv et al. compensated for these differences by adjusting their multivariate analysis with molecular subtypes. Overall, this result should be taken with caution.

### Genetic variations

Amongst pooled reported associations of pCR for genetic variations, our study evaluated the effect of *PIK3CA* and *TP53* genes in NAC-treated Asian BC patients. Notably, no specific or single variant vs wildtype was addressed for both genes as well. In the adjusted analysis of three [51, 100, 106] and two studies [51, 100] for *PIK3CA* and *TP53* genes, respectively, breast cancer patients harbouring mutation in the *PIK3CA* gene was associated with worse response (OR 0.64; 95% CI 0.42-0.98; *p*=0.04; Supplementary Figure 7.2B in Additional file 7) while *TP53* gene was not associated with pCR outcome (OR 1.34; 95% CI 0.59–3.05; *p*=0.49; Supplementary Figure 7.2B in Additional file 7).

### Biomarkers

Amongst pooled reported associations of pCR for biomarkers, our study evaluated the effect of Tau, nm23-H1, ER, PR, HR, HER2, and Ki-67 biomarkers in NAC-treated Asian BC patients (Supplementary Figures 7.2–7.8 in Additional file 7). In an adjusted analysis of Tau pooling two studies [61, 83], the result suggests that Tau+ was associated with worse response in the neoadjuvant setting (OR 0.22, 95% CI 0.09–0.54, *p*=0.0008; Supplementary Figure 7.2C in Additional file 7). Meanwhile in adjusted analysis of nm23-H1 pooling two studies [12, 60], nm23-H1 was not associated with pCR outcome in TA-treated patients (OR 1.56, 95% CI 0.55–4.45, *p*=0.41; Supplementary Figure 7.2C in Additional file 7).

The association between pCR in NAC-treated breast cancer patients and ER was evaluated in Supplementary Figure 7.3 (Additional file 7). In the crude analysis of ER+ vs ER− pooling five studies [61, 94, 97, 112, 115], ER was not associated with pCR outcome (OR 0.39; 95% CI 0.13–1.15; *p*=0.09). The reported moderate heterogeneity between the studies was perhaps due to the difference in favoured outcomes in one study [112] compared to the rest. Moreover, the heterogeneity is attributable to the different NAC treatments received in each study, whereby the patients were either treated with TP or anthracycline-containing regimens. Therefore, subgroup analyses of ER− vs ER+ pooling studies with patients treated with anthracycline-containing chemotherapy [61, 112] and taxane-platinum chemotherapy [94, 97, 115] were conducted. Our analysis revealed that ER was not associated with pCR outcome when patients were treated with an anthracycline-containing agent (OR 1.19; 95% CI 0.07–19.28; *p*=0.90) with considerable heterogeneity observed between the studies. The heterogeneity is perhaps due to differences in the favoured outcome in each study caused by the addition of the taxane regimen with the anthracycline in Li et al. [61]. Contrarily, ER+ patients were significantly associated with worse response when treated with TP (OR 0.19; 95% CI 0.11–0.32; *p*<0.00001).

Meanwhile, adjusted analysis of ER+ vs ER− pooling fourteen studies [54, 56, 61, 68, 71, 72, 83, 94, 97, 102, 103, 106, 112, 131] also showed that ER was not associated with pCR outcome (OR 0.59; 95% CI 0.32–1.08; *p*=0.09; Supplementary Figure 7.3 in Additional file 7). The observed substantial heterogeneity between the studies was perhaps due to differences in the chemotherapeutic agents received in each study. Subgroup analysis pooling five studies [56, 72, 83, 106, 131] with patients treated in the neoadjuvant setting revealed that ER was not associated with pCR outcome (OR 0.47; 95% CI 0.19–1.14; *p*=0.09) with considerable heterogeneity reported between the studies probably due to clinical variances between the studies. Another two subgroup analyses pooling studies with patients treated with TP [94, 97] and TA [54, 61, 71] regimens indicate that ER+ was significantly associated with worse response (OR 0.21; 95% CI 0.06–0.70; *p*=0.01 and OR 0.34; 95% CI 0.19–0.61; *p*=0.0003, respectively). While subgroup analysis pooling studies with patients treated with anthracycline-based chemotherapy [102, 112] showed that ER was not associated with pCR response (OR 4.29; 95% CI 0.67–27.39; *p*=0.12). Substantial heterogeneity was reported probably due to the difference in effect size between the studies attributed to the study sample size, whereby Zhao et al. [112] recruited 98 locally advanced breast cancer patients while Yao et al. [102] recruited 538 breast cancer patients.

Meanwhile, analyses pooling studies based on the characteristics of the patients showed that ER was not associated with pCR in patients achieving a complete pathological response in the breast only (OR 0.93; 95% CI 0.18–4.85; *p*=0.94; Supplementary Figure 7.3 in Additional file 7). The observed heterogeneity between the studies is probably due to the smaller number of studies pooled (*n*=2) and each study favoured a different outcome. Notably, ER− patients were significantly associated with pCR in studies pooling anthracycline-treated patients (OR 2.78; 95% CI 1.61–4.78; *p*=0.0002; Supplementary Figure 7.3 in Additional file 7).

The role of the biomarker PR was assessed in Asian breast cancer patients subjected to neoadjuvant chemotherapy (Supplementary Figure 7.4 in Additional file 7). In an analysis with crude OR results pooling five studies [61, 94, 97, 112, 115], it was observed that patients with PR+ were significantly associated with worse response (OR 0.40; 95% CI 0.20–0.79; *p*=0.009). The observed substantial heterogeneity between the studies was perhaps due to the difference in the favoured outcome in one study [112] and differences in weightage and population size. Subgroup analysis pooling studies with TP-treated patients [94, 97, 115] revealed PR+ patients were significantly associated with worse response (OR 0.29; 95% CI 0.13–0.62; *p*=0.001). Although moderate heterogeneity was reported, all the three studies pooled for the subgroup analysis were conducted in the Chinese population with no clinical variances. Thus, the heterogeneity result was rejected. Meanwhile, in subgroup analysis pooling patients treated with anthracycline-containing NAC [61, 112], the biomarker PR was not associated with treatment response (OR 0.66; 95% CI 0.17–2.62; *p*=0.55).

PR was also not associated with treatment response in the adjusted analysis pooling eight studies [54, 56, 61, 94, 97, 102, 103, 106] (OR 1.01; 95% CI 0.64–1.60; *p*=0.97; Supplementary Figure 7.4 in Additional file 7). The observed moderate heterogeneity was probably due to differences in population size and differences in treatment given in each study. We then conducted four subgroup analyses focusing on the treatment regimen and found that PR was not associated with treatment response in breast cancer patients treated: (1) in the neoadjuvant setting (OR 1.01; 95% CI 0.47–2.18; *p*=0.99); (2) with TP (OR 0.94; 95% CI 0.34–2.58; *p*=0.91); and (3) TA regimens (OR 0.37; 95% CI 0.05–2.94; *p*=0.35), but PR− was significantly associated with better response in anthracycline-containing treated patients (OR 1.63; 95% CI 1.03–2.57; *p*=0.04). Meanwhile, in adjusted analysis pooling studies with anthracycline-treated patients [88, 96] revealed that PR was not significantly associated with treatment response (OR 1.43; 95% CI 0.74–2.77; *p*=0.29).

The association between treatment response and hormone receptors (HR) comprising ER and PR was assessed in Asian breast cancer patients (Supplementary Figure 7.5 in Additional file 7). In an analysis with crude OR pooling four studies [51, 62, 64, 116], it was observed that patients with HR+ were significantly associated with worse treatment response in the neoadjuvant setting (OR 0.47; 95% CI 0.24–0.92; *p*=0.03). In a subgroup analysis whereby all the recruited patients in the pooled studies [51, 64] were of HER2+, it was observed that HR+ were significantly associated with a worse response (OR 0.40; 95% CI 0.18–0.89; *p*=0.02). Excluding the aforementioned studies, subgroup analysis pooling two studies [62, 116] revealed that HR was not significantly associated with treatment response in the neoadjuvant setting (OR 1.16; 95% CI 0.22–6.22; *p*=0.87).

Similarly, analysis with adjusted OR pooling five studies [51, 60, 62, 64, 70] indicated that HR was not significantly associated with treatment response (OR 1.27; 95% CI 0.47–3.45; *p*=0.64; Supplementary Figure 7.5 in Additional file 7). The observed substantial heterogeneity was perhaps due to the clinical variance in the characteristics of the recruited breast cancer population in each study. Subgroup analysis pooling studies analysing HR+ vs HR− in the neoadjuvant setting [62, 70] showed that HR− were significantly associated with better treatment responses (OR 2.39; 95% CI 1.17–04.87; *p*=0.02). Meanwhile, subgroup analysis pooling studies with HER2+ patients [51, 64] revealed that HR+/HER2+ breast cancer were significantly associated with worse treatment responses in the neoadjuvant setting (OR 0.43; 95% CI 0.21–0.88; *p*=0.02).

The association between pCR outcome and HER2 was estimated in Supplementary Figure 7.6 (Additional file 7). In an analysis pooling crude OR of seven studies [61, 62, 77, 94, 97, 112, 115], it was observed that HER2+ breast cancer patients were significantly associated with better treatment response (OR 2.50; 95% CI 1.44–4.35; *p*=0.001). The moderate heterogeneity reported between the studies was perhaps due to the differences in treatment regimens given to the recruited breast cancer population in each study. Subsequently, two subgroup analyses pooling studies according to the chemotherapy regimens administered to the breast cancer patients revealed that HER2+ patients treated with TP regimen [94, 97, 115] were significantly associated with better response (OR 4.64; 95% CI 2.74–7.86; *p*<0.00001), while HER2 was not associated with treatment response in patients treated with anthracycline-containing regimen (84,141) (OR 2.08; 95% CI 0.90–4.78; *p*=0.09).

Similarly, in an analysis pooling adjusted OR of 12 studies [56, 60, 61, 70, 77, 83, 94, 97, 102, 103, 106, 131], it was observed that HER2+ breast cancer patients were significantly associated with better treatment response (OR 2.29; 95% CI 1.56–3.35; *p*<0.0001; Supplementary Figure 7.6 in Additional file 7). Substantial heterogeneity was reported, perhaps due to the clinical variances in each study, based on the treatment received by the patients and the characteristics of the recruited breast cancer population. Subgroup analysis pooling five studies in the neoadjuvant setting [56, 70, 83, 106, 131] showed patients with HER2+ were significantly associated with better response (OR 2.33; 95% CI 1.31–4.15; *p*=0.004). Some differences between the studies might explain the observed substantial heterogeneity: (1) two of the studies [56, 83] main objective was to investigate the association of Tau with response to neoadjuvant chemotherapy, while Yuan et al. [106] focused on the association of *PIK3CA* mutation status with response to neoadjuvant chemotherapy, and Lim et al. [131] and Lv et al. [70] assessed factors affecting neoadjuvant treatment response; (2) Lim et al. [131] was the only study including multi-ethnic cohort of breast cancer patients since it was conducted in Singapore and Malaysia, although one of the ethnicity included in Lim et al. was Chinese. Meanwhile, subgroup analyses pooling two studies of patients treated with TP [94, 97] and two studies of patients treated with anthracycline-containing chemotherapy [102, 103] indicate HER2+ patients were significantly associated with better treatment response (OR 7.07; 95% CI 2.88–17.40; *p*<0.0001 and OR 2.65; 95% CI 1.66–4.23; *p*<0.0001, respectively). However, subgroup analysis pooling two studies of patients treated with TA [60, 61] revealed that HER2 was not associated with treatment response (OR 1.08; 95% CI 0.47–2.51; *p*=0.85).

Notably, HER2 was also not associated with pCR in patients achieving a complete pathological response in the breast only (OR 1.96; 95% CI 0.78–4.89; *p*=0.15; Supplementary Figure 7.6 in Additional file 7) and in anthracycline-treated patients (OR 1.52; 95% CI 0.97–2.40; *p*=0.07; Supplementary Figure 7.6 in Additional file 7).

As for Ki-67, an analysis pooling crude OR of eight studies [46, 51, 61, 64, 94, 97, 115, 123] observed that patients with high Ki-67 were significantly associated with better treatment responses (OR 2.63; 95% CI 1.69–4.07; *p*<0.0001; Supplementary Figure 7.7 in Additional file 7). Subgroup crude analyses pooling studies with TNBC patients [46, 123] and patients treated with TP [94, 97, 115] and TA [46, 61] revealed that patients with high Ki-67 were significantly favoured to achieve better treatment responses (OR 4.42; 95% CI 1.41–13.85; *p*=0.01, OR 2.13; 95% CI 1.21–3.75; *p*=0.009, and OR 4.26; 95% CI 1.90–9.54; *p*=0.0004, respectively). Meanwhile, subgroup crude analysis pooling studies with HER2+ patients [51, 64] showed that Ki-67 was not associated with treatment response (OR 1.55; 95% CI 0.79–3.07; *p*=0.20).

Similarly, an analysis of adjusted OR pooling seven studies [12, 46, 51, 54, 94, 97, 110] showed that patients with high Ki-67 were significantly associated with better treatment response (OR 2.63; 95% CI 1.56–4.41; *p*=0.0003; Supplementary Figure 7.7 in Additional file 7). In subgroup adjusted analysis pooling studies with HER2+ patients [51, 110], breast cancer patients with high Ki-67 were significantly associated with better response in the neoadjuvant setting (OR 3.67; 95% CI 1.11–12.12; *p*=0.03). Congruent with the subgroup crude analysis, a subgroup adjusted analysis of pooled studies with TNBC patients [12, 46] also revealed patients with high Ki-67 were significantly associated with better treatment response (OR 2.16; 95% CI 1.00–4.64; *p*=0.05). Notably, the analysis was also influenced by the fact that the TNBC patients were treated with the TA regimen. Breast cancer patients with high Ki-67 were also significantly associated with better treatment response when treated with TA regimen (OR 2.24; 95% CI 1.34–3.74; *p*=0.002). However, in patients treated with NAC TP, Ki-67 was not associated with treatment response (OR 4.63; 95% CI 0.35–61.14; *p*=0.24).

Three biomarkers—ER, HR, and Ki-67—were evaluated by pooling studies reporting their association using hazards ratio (Supplementary Figure 7.8 in Additional file 7). Our pooled adjusted analysis of two studies [73, 80] showed that ER− patients were significantly associated with better treatment response (HR 2.75; 95% CI 1.25–6.05; *p*=0.01). Both crude and adjusted analysis of HR− vs HR+ and high vs low Ki-67 in HER2+ patients treated with taxane-containing chemotherapy showed that HR− patients and patients with high Ki-67 were significantly associated with a better response. However, in an adjusted result analysis of high vs low Ki-67 in patients treated with taxane-containing chemotherapy, Ki-67 was not significantly associated with treatment response (HR 1.26; 95% CI 1.26–8.25; *p*=0.81) with substantial heterogeneity. The significant heterogeneity might be explained by the variation in the taxane-based treatment regimen where Zhang et al. [107] included patients submitted to either single taxane-based regime or taxane-platinum combination, while all patients recruited in Wang et al. [80] and Ding et al. [47] were submitted to taxane-anthracycline and taxane-platinum combination regimens, respectively. Moreover, Zhang et al. and Ding et al. incorporated trastuzumab as part of their neoadjuvant regimen while Wang et al. subjected their patients to trastuzumab in the adjuvant setting.

### Publication bias

Publication bias assessment was done using the Jamovi Software (version 2.3) [133] (Supplementary Figures 8.1- 8.13 in Additional file 8). The occurrence of publication bias was observed in two analyses.

First, in the overall analysis evaluating the association of pCR outcome with HER2+ and HER2− breast cancer patients submitted to TA chemotherapy, the regression test indicated funnel plot asymmetry (*p*=0.03) but not the rank correlation test (*p*=0.34). File drawer analysis indicated that at least 51 studies would be required to nullify the effect (*p*<0.001). Hence, there is less chance of publication bias in the analysis. As indicated in Fig. 5, subgroup analysis was not conducted for the overall analysis because although the effect is estimated to favour HER2+ significantly, in some studies the true effect may in fact favour HER2−.

Second, in the overall analysis evaluating the association of treatment response with PR− and PR+ breast cancer patients in the neoadjuvant setting, the rank correlation test indicated funnel plot asymmetry (*p*=0.03) but not the regression test (*p*=0.08). File drawer analysis suggested the presence of publication bias in the analysis, which could be explained by the heterogeneity observed between the pooled studies, specifically in the treatment regimens assessed in each study. Thus, four subsequent subgroup analyses—pooling two studies at each treatment regimen the patients were subjected to—were conducted for the overall PR− vs PR+ (adjusted results) analysis (Supplementary Figure 7.4 in Additional file 7).

## Discussion

There are many options for breast cancer treatment. Often, this includes surgery, radiotherapy, and systemic therapy comprising chemotherapy with or without targeted therapy. Systemic treatment is commonly decided based on the target biomarkers, ensuring the treatment would be effective. Improvements in systemic therapy and targeted therapies utilising an individual’s diagnosis have improved overall pCR rates in breast cancer patients. Studies reported pCR benefits of 44.4% (*n*=8/18) in TNBC patients treated with NAC TP than 0% (*n*=0/9) in Luminal A [69], 8.9% (*n*=56/632) in ER+ breast cancer patients treated with NAC TA than 19.8% (*n*=75/378) in ER− patients [36], and 24.7% (*n*=55/223) in breast cancer patients with wildtype *PIK3CA* gene treated with NAC TA than 11.7% (*n*=11/94) in mutated *PIK3CA* [106]. Preoperative chemotherapy or neoadjuvant chemotherapy is typically the standard of care in treating both operable and inoperable locally advanced breast cancer owing to its advantage for breast conservation surgery and aids in shrinking an inoperable tumour to improve resectability [134]. The success of neoadjuvant chemotherapy associated with treatment response (pCR) could be used as a prognostic value in managing breast cancer since the efficacy of a treatment often translates into a highly favourable overall survival and disease-free progression. Often, the best pCR outcomes were observed with TNBC, moderate for HER2E, and the worst for Luminal [134]. Notably, different breast cancer subtypes have different sensitivities to NAC and frequently, a combination of them is given to be effective.

We systematically evaluated the effect of breast cancer molecular subtypes, biomarkers, and genetic variations on breast cancer treatment in Asian breast cancer patients, focusing on treatment response (pCR) in the neoadjuvant setting. In our study, the common NAC treatments used in the pooled analyses were TA, TP, anthracycline-based, and taxane-based chemotherapies with reported pCR rates of 2.7–64.7%, 7.7–60%, 4.3–35%, and 11.3–57.1%, respectively owing to the molecular subtypes, biomarkers, and genetic variations present in the breast cancer patients. There were limited studies focused on breast cancer treatment in Asian breast cancer patients utilising the molecular subtypes classification of breast cancer clinically. In particular, most studies reported the association between breast cancer treatment response with biomarkers and/or genetic variations. From our study, the most frequent subtypes in the Asian population are TNBC and HER2E, followed by luminal B and luminal A. This trend was not consistent with previous studies on the Asian population conducted on 560 Malaysian breast cancer tumours [24] and 2791 Chinese women with breast cancer [76]. Notably, the subtype frequencies in our study might not reflect the entirety of the Asian population. Most of the studies included in this paper were mainly from the Chinese population and fewer of the others (Korean, Japanese, Malaysian, and Indian). Moreover, not all the studies included focused on all four subtypes. Some studies have a different definition of a luminal A, luminal B, and luminal-like subtype when using IHC as a surrogate to classify breast cancer since it is readily available and cost-effective than gene panels.

Our findings suggest Asian TNBC patients subjected to TA and TP in the neoadjuvant setting were observed to favour better response. In particular, although TNBC subtypes were reported to be more likely to benefit from NAC treatment compared to non-TNBC, it is significantly associated through statistical analysis with better response when treated with NAC TP than TA in our study. These findings are consistent with meta-analysis conducted in the general population by Pandy et al. [135] pooling 2415 breast cancer patients treated with NAC TA and TP, which revealed that there was an improvement in the pCR rates in TP-treated patients (44.6%) compared to TA-treated patients (27.8%). TNBC has a poor prognosis and is a more aggressive subtype, with a higher recurrence rate and metastasis (138). Due to the lack of receptor expression, it is not responsive to hormonal therapy [136]. While pCR was observed to be higher in TNBC, which often translates to a desirable long-term outcome [75], some studies have observed the opposite effect whereby there was no difference in survival in patients that have achieved pCR [137, 138]. Similarly, our study showed that HER2E patients were more likely to achieve pCR benefit from NAC TP (52.4%) than TA treatment (27.8%). HER2E is an aggressive subtype with a poor prognosis [139]. However, it can be sensitive to cytotoxic chemotherapy and slightly resistant to hormonal therapy and has shown some positive outcomes with targeted therapy [139–141].

Our study revealed that patients with luminal A subtype were less likely to benefit from TA (7.7%) and TP (4.3%) regimens in the neoadjuvant setting. Meanwhile, luminal B has a slightly better pCR rate when treated with TP (28.1%) and TA (12%) and similarly, luminal-like treated with TP (32.5%) and TA (9%). Despite poor responses to NAC, luminal A has a good prognosis [142] with a significantly lower relapse rate than other subtypes, and usually responds well to hormonal therapy [141, 142]. Luminal B has a slightly worse prognosis than luminal A, with an increased chance of recurrence rate, decreased survival rate after relapse, and less sensitivity to hormonal therapy [141, 143]. Notably, the distinct characteristics of each subtype make it challenging to find a treatment that would be effective for all of them. Thus, a combination of chemotherapy, targeted therapy, and endocrine therapy is frequently utilised. Taken together, our study showed that each subtype responds differently to different single or combination of chemotherapeutic agents in the neoadjuvant setting.

Our study’s evaluation of the effect of biomarkers on the pCR outcome suggested the commonly reported biomarkers ER, PR, HER2, and Ki-67 as predictors of the likelihood of better pCR outcomes in breast cancer patients. In particular, patients with negative expression of ER and PR, positive expression of HER2, and higher expression of Ki-67 are significantly associated with better pCR outcomes when treated with either TA or TP chemotherapeutic regimens in the neoadjuvant setting. Patients with negative expression of PR and HER2 are also significantly associated with better pCR outcomes when treated with an anthracycline agent, while patients with HR-negative (ER− and PR−) are significantly associated with better pCR outcomes when treated with taxane. In contrast, although Łukasiewicz et al. supported the expression of ER, PR, HER2, and Ki-67 as predictive and potential prognostic factors, the updated review on breast cancer specified that patients with higher expression of these biomarkers are those who are usually present significantly better clinical outcomes [144]. Unfortunately, Łukasiewicz et al. did not review the predictive or prognostic value of the biomarkers concerning specific chemotherapeutic regimens.

The proper Ki-67 cut-off value in positive hormone receptors (HR+) breast cancer was often discussed due to its importance in evaluating the aggressiveness of the cancer and to distinguish between luminal A and luminal B (HER2−) subtypes when IHC is used as a surrogate classification since both subtypes are of ER+ and HER2− [5, 144]. Furthermore, experts at the St Gallen Consensus Meeting have changed the threshold for Ki-67 over time, from 14% in 2011 to 20% in 2013 [5]. Without the establishment of an optimal Ki-67 cut-off value, it has become challenging to discern what constitutes truly a high or low proliferation of Ki-67 in some studies which led to low reproducibility for the Ki-67 marker. In resolving this issue, the International Ki67 in Breast Cancer Working Group (IKWG) agrees that without improvements in the standardisation of the Ki-67 cut-off value, routine, non-trial settings can reliably categorise very low Ki-67 as ≤5% and very high as ≥30% [145]. Notably, our study congruently suggested that high Ki-67 is significantly associated with better treatment response only at 14 and 20% cut-off values.

Our meta-analysis findings also suggested other biomarkers such as nm23-H1 and CK5/6 as predictors in TA-treated Asian breast cancer patients and Tau in NAC-treated patients. These other biomarkers can be further evaluated and utilised as potential targets in treating breast cancer due to their involvement in the cell signalling pathway and cell division [12, 60]. Tau protein behaves as a microtubule-association protein, which can be found in normal breast epithelial and cancer cells [61]. The expression of Tau protein was found higher in metastatic breast cancer and is often associated with better prognosis and better response to taxanes [61, 146]. Interestingly, our findings suggest the opposite effect where Tau- breast cancer was favoured to have a better response when treated with taxane-containing chemotherapy. However, this could be due to the clinical variance in the study pooled in our analysis. In particular, breast cancer patients in Li et al. [61] were treated with combination TA regimens, while patients in Wang et al. [83] were treated with TP regimens. Furthermore, both studies excluded metastatic breast cancer patients from their study. Meanwhile, CK5/6—often used to define basal-like TNBC—is an intermediate filament protein that provides structure to the cell and is also associated with poor prognosis [90]. CK5/6 and EGFR expression were accepted as biomarkers for classification of Basal-like breast cancer within the TNBC subtype [90].

There were several genes included for analysis in this study. Our meta-analysis results suggested the *PIK3CA* gene as a predictor for pCR in TA-treated breast cancer patients with *PIK3CA* mt (17.1%) and *PIK3CA*wt (24.7%) [106]. Similarly, patients harbouring mutated *PIK3CA* gene in the Caucasian population who received either trastuzumab, lapatinib, or the combination in addition to a taxane-based chemotherapy are associated with a lower pCR rate [147]. *PIK3CA* mutations are commonly found in the luminal subtypes, involving signalling pathways to attain a multi-lineage potential, leading to resistance to endocrine therapy [148]. The current treatment recently approved by the US FDA for breast cancer with *PIK3CA* mutation includes an oral medication Alpelisib acting as PI3K Alpha-Selective Inhibitor. This works as an inhibitor on the common mutation site and induces p110α degradation. This type of PIK3 inhibitor showed more tolerable effects in patients. However, substantial toxicities are still present [149, 150].

Although our study did not reveal a significant association between pCR and the *TP53* gene, *TP53* is one of the frequently mutated genes in breast cancer and has an involvement in gene transcription that curates cell cycle processes, apoptosis, and DNA repair [151]. *TP53* mutations are correlated with HER2+, HR−, and high Ki-67 and contribute to the aggressive characteristics of cancer, leading to treatment resistance [152]. Therefore, it is often associated with a poor prognosis. The molecular profile involving gene expression has been associated with breast cancer recurrence. While the molecular profiling of advanced breast cancer is crucial, it can also be very costly. There is a complex interplay between the genes and the signal pathways which subsequently affect the pCR outcome that needs to be further understood.

In this study, we have explored the role of molecular subtypes, biomarkers, and genetic variations on treatment outcome. It was evident that the treatment given to patients based on their breast cancer characterisation in the neoadjuvant settings has yielded variable pCR outcome. The combination of classifying the patient’s molecular subtypes and identifying the status of their biomarkers can effectively predict the treatment outcome. Apart from use of the routine biomarkers (ER, PR, and HER2), further classification and diagnosis can be done utilising several of the non-conventional biomarkers (Tau, CK5/6, EGFR, Topo-II) and genes (*TP53* and *PIK3CA*) especially in the Asian population in hopes to achieve better individualised treatment through PPM. Thus, there is a need for high-quality validated biomarkers that can predict treatment responses.

### Limitations

Despite the efforts during the development and completion of this study, some limitations need to be addressed. First, our study is inclusive of different analytical study designs such as observational (case-control and cohort study) and experimental (randomised controlled trials and non-randomised controlled trials) which might be prone to bias and heterogeneity when they are pooled for analysis. Second, the number of individual studies pooled for each of the analyses was mostly small, both in the overall and subgroup analyses. Third, for a considerable number of studies, there was a need to (1) indirectly categorised their molecular subtypes based on IHC which may not be entirely correct since we are only utilising the data available in the article, and (2) indirectly calculate the 95% CI value in RevMan which may deviate from the original value. Lastly, our tests for publication bias should be considered carefully as the number and size of studies included were limited. Notwithstanding the first limitation of this study, the inclusion of several different analytical study designs allowed us to increase the number of individual studies gathered for the identification of genetic determinants of treatment outcome in breast cancer patients submitted to neoadjuvant chemotherapy. Moreover, we have accounted for heterogeneity using best practices that are consistent with those employed by The Cochrane Collaboration [153]. Nonetheless, future studies should be focused on each of the breast cancer characterisations to validate our findings. In particular, future studies focusing on investigating the effect of specific variant of a gene—utilising the homozygous and heterozygous nature of the variant—against their wildtype on treatment outcome in Asian breast cancer patients will justify the importance and benefits of PPM.

## Conclusions

To the best of our knowledge, this SLR is the only comprehensive review currently available that analyses the effect of molecular subtype classification, biomarkers, and genetic variations on the pCR outcome of breast cancer patients in the neoadjuvant setting focusing on the Asian population. The SLR search spanned 20 years and identified over 6000 records which was further reduced to 3725 records after the exclusion of non-Asian breast cancer patients, suggesting that although this area is still understudied, there is a growing interest to pursue the research area. Notably, this SLR adhered to best practices and followed PRISMA reporting guidelines. Our findings justified that molecular subtype (HER2E and TNBC), biomarkers (ER, PR, HER2, HR, Ki-67, nm23-H1, CK5/6, and Tau), and gene (*PIK3CA*) could be further explored for their possible role in first-line treatment response in Asian breast cancer clinical studies. Understanding the effect of these determinants might be a crucial step to tailor treatment to each patient, which can avoid overtreatment of the tumour with non-aggressive nature and undertreatment of the tumour with aggressive nature. Thus, with further validation, this information can be utilised to treat breast cancer more efficiently in the Asian population.

### Supplementary Information

**Additional file 1.** PRISMA 2020 Checklist.

**Additional file 2.** Search term strategy. **Supplementary Table 2.1.** The keywords and search terms formulated from the SLR PICO question. **Supplementary Table 2.2.** Search strategy and results from MEDLINE (PubMed) database. **Supplementary Table 2.3.** Search strategy and results from Science Direct database. **Supplementary Table 2.4.** Search strategy and results from Scopus database. **Supplementary Table 2.5.** Search strategy and results from Cochrane Library database.

**Additional file 3.** Data Extraction. **Supplementary Table 3.1.** Extraction of data from the included studies in the systematic literature review and meta-analysis. **Supplementary Table 3.2.** Extraction of pCR data from the included studies in the systematic literature review and meta-analysis.

**Additional file 4.** Data Synthesis. **Supplementary Table 4.1.** Data synthesised for overview of all included studies. **Supplementary Table 4.2.1.** Data synthesised for the association of breast cancer treatment response according to breast cancer characterisation. **Supplementary Table 4.2.2.** Analysis of breast cancer treatment response according to chemotherapeutic agents in different breast cancer characteristics. **Supplementary Table 4.3.** Pooled reported association of pCR in included studies. **Supplementary Table 4.4.** Evaluation of synthesised tpCR data. **Supplementary Table 4.5.** Evaluation of synthesised tpCR data. **Supplementary Table 4.6.** Evaluation of synthesised pooled reported association of pCR data.

**Additional file 5.** Modified Newcastle-Ottawa Quality Assessment Scale.

**Additional file 6.** Quality of the included studies. **Supplementary Table 6.1.** Newcastle-Ottawa Scale of each included cohort study. **Supplementary Figure 6.1.** Risk of bias by domain and question in 87 cohort studies using Newcastle-Ottawa Scale. Numbers on the green bar represent the number of studies with low risk of bias over the number of studies assessed. **Supplementary Table 6.2.** Newcastle-Ottawa Scale of each included case-control study. **Supplementary Figure 6.2.** Risk of bias by domain and question in 14 case-cohort studies using Newcastle-Ottawa Scale. Numbers on the green bar represent the number of studies with low risk of bias over the number of studies assessed.

**Additional file 7.** Meta-analysis results. **Supplementary Figure 7.1.** Pooled pCR outcome of NAC-treated Asian breast cancer patients*.* Forest plots describing the random effect ORs and 95% CIs from studies assessing the association of pCR outcome in: NAC TA-treated breast cancer patients between (A) TNBC and HER2E; (B) Luminal B and Luminal A; NAC TP-treated breast cancer patients between (C) HER2E and TNBC; NAC TA-treated breast cancer patients with (D) EGFR. I^2^ and *p*-value for X^2^ of heterogeneity are reported for each group analysis. **Supplementary Figure 7.2.** Pooled reported association of pCR in NAC-treated Asian breast cancer patients presented in different variables*.* Forest plots describing the random effect ORs and 95% CIs from studies assessing the pooled reported association of pCR in NAC-treated breast cancer patients presented according to (A) Molecular classification; (B) Genetic variations; and (C) Biomarkers. I^2^ and *p*-value for X^2^ of heterogeneity are reported for each group analysis. **Supplementary Figure 7.3.** Pooled reported association of pCR in NAC-treated Asian breast cancer patients with ER. Forest plot describing the random effect ORs and 95% CIs from studies assessing the association between the biomarker ER and pCR in NAC-treated breast cancer patients. I^2^ and *p*-value for X^2^ of heterogeneity are reported for each group analysis. **Supplementary Figure 7.4.** Pooled reported association of pCR in NAC-treated Asian breast cancer patients with PR. Forest plot describing the random effect ORs and 95% CIs from studies assessing the association between the biomarker PR and pCR in NAC-treated breast cancer patients. I^2^ and *p*-value for X^2^ of heterogeneity are reported for each group analysis. **Supplementary Figure 7.5.** Pooled reported association of pCR in NAC-treated Asian breast cancer patients with HR*.* Forest plot describing the random effect ORs and 95% CIs from studies assessing the association between the hormone receptors (HR) – comprising ER and PR – and pCR in NAC-treated breast cancer patients. I^2^ and *p*-value for X^2^ of heterogeneity are reported for each group analysis. Supplementary Figure 7.6. Pooled reported association of pCR in NAC-treated Asian breast cancer patients with HER2*.* Forest plot describing the random effect ORs and 95% CIs from studies assessing the association between the biomarker HER2 and pCR in NAC-treated breast cancer patients. I^2^ and *p*-value for X^2^ of heterogeneity are reported for each group analysis. **Supplementary Figure 7.7.** Pooled reported association of pCR in NAC-treated Asian breast cancer patients with Ki-67*.* Forest plot describing the random effect ORs and 95% CIs from studies assessing the association between Ki-67 and pCR in NAC-treated breast cancer patients. I^2^ and *p*-value for X^2^ of heterogeneity are reported for each group analysis. **Supplementary Figure 7.8.** Pooled reported association of pCR in NAC-treated Asian breast cancer patients presented in different biomarkers. Forest plot describing the random effect HRs and 95% CIs from studies assessing the association between pCR in NAC-treated breast cancer patients and the biomarkers ER, HR, and Ki-67. I^2^ and *p*-value for X^2^ of heterogeneity are reported for each group analysis.

**Additional file 8. **Publication Bias. **Supplementary Figure 8.1.** Funnel plot of pooled pCR outcome of Asian breast cancer patients treated with TA chemotherapy*.* Funnel plot assessing the publication bias in evaluating the effect of HER2E and Luminal, combined in breast cancer pCR outcome of patients treated with TA regimen in the neoadjuvant setting. **Supplementary Figure 8.2.** Funnel plot of pooled pCR outcome of Asian breast cancer patients treated with TA chemotherapy*.* Funnel plot assessing the publication bias in evaluating the effect of TNBC and Luminal, combined in breast cancer pCR outcome of patients treated with TA regimen in the neoadjuvant setting. **Supplementary Figure 8.3.** Funnel plot of pooled pCR outcome of Asian breast cancer patients treated with TA chemotherapy. Funnel plot assessing the publication bias in evaluating the effect of TNBC and HER2E in breast cancer pCR outcome of patients treated with TA regimen in the neoadjuvant setting. **Supplementary Figure 8.4.** Funnel plot of pooled pCR outcome of Asian breast cancer patients treated with TP chemotherapy*.* Funnel plot assessing the publication bias in evaluating the effect of HER2E and Luminal, combined in breast cancer pCR outcome of patients treated with TP regimen in the neoadjuvant setting. **Supplementary Figure 8.5.** Funnel plot of pooled pCR outcome of Asian breast cancer patients treated with TA chemotherapy. Funnel plot assessing the publication bias in evaluating the effect of ER in breast cancer pCR outcome of patients treated with TA regimen in the neoadjuvant setting. **Supplementary Figure 8.6.** Funnel plot of pooled pCR outcome of Asian breast cancer patients treated with TA chemotherapy*.* Funnel plot assessing the publication bias in evaluating the effect of HER2 in breast cancer pCR outcome of patients treated with TA regimen in the neoadjuvant setting. **Supplementary Figure 8.7.** Funnel plot of pooled pCR outcome of Asian breast cancer patients treated with TA chemotherapy. Funnel plot assessing the publication bias in evaluating the effect of Ki-67 in breast cancer pCR outcome of patients treated with TA regimen in the neoadjuvant setting. **Supplementary Figure 8.8.** Funnel plot of pooled pCR association of Asian breast cancer patients in the neoadjuvant setting*.* Funnel plot assessing the publication bias in evaluating the effect of ER+ and ER- (adjusted OR association) in breast cancer pCR outcome of patients treated in the neoadjuvant setting. **Supplementary Figure 8.9.** Funnel plot of pooled pCR association of Asian breast cancer patients in the neoadjuvant setting*.* Funnel plot assessing the publication bias in evaluating the effect of PR- and PR+ (adjusted OR association) in breast cancer pCR outcome of patients treated in the neoadjuvant setting. **Supplementary Figure 8.10.** Funnel plot of pooled pCR association of Asian breast cancer patients in the neoadjuvant setting*.* Funnel plot assessing the publication bias in evaluating the effect of HER2- and HER2+ (crude OR association) in breast cancer pCR outcome of patients treated in the neoadjuvant setting. **Supplementary Figure 8.11.** Funnel plot of pooled pCR association of Asian breast cancer patients in the neoadjuvant setting*.* Funnel plot assessing the publication bias in evaluating the effect of HER2+ and HER2- (adjusted OR association) in breast cancer pCR outcome of patients treated in the neoadjuvant setting. **Supplementary Figure 8.12.** Funnel plot of pooled pCR association of Asian breast cancer patients in the neoadjuvant setting*.* Funnel plot assessing the publication bias in evaluating the effect of high and low Ki-67 (crude OR association) in breast cancer pCR outcome of patients treated in the neoadjuvant setting. **Supplementary Figure 8.13.** Funnel plot of pooled pCR association of Asian breast cancer patients in the neoadjuvant setting*.* Funnel plot assessing the publication bias in evaluating the effect of high and low Ki-67 (adjusted OR association) in breast cancer pCR outcome of patients treated in the neoadjuvant setting.

## Data Availability

All data generated or analysed during this study are included in this published article and its supplementary information files.

## Abbreviations

CI: Confidence interval
EGFR: Epidermal growth factor receptor
ER: Oestrogen receptor
HER2: Human epidermal growth factor receptor-2
HER2E: Human epidermal growth factor receptor-2 enriched
HR: Hazards ratio
IHC: Immunohistochemistry
NAC: Neoadjuvant chemotherapy
OR: Odds ratio
pCR: Pathological complete response
PR: Progesterone receptor
TA: Taxane-anthracycline
TNBC: Triple-negative breast cancer
TP: Taxane-platinum
