# Supplementary material for: Exploring the effectiveness of molecular subtypes, biomarkers, and genetic variations as first-line treatment predictors in Asian breast cancer patients: a systematic review and meta-analysis
Source: Syst Rev. 2024 Apr 4;13:100. doi: 10.1186/s13643-024-02520-5 (PMC10993489; doi:10.1186/s13643-024-02520-5)
Supplement: Supplementary file 2 — Additional file 2. Search term strategy. Supplementary Table 2.1. The keywords and search terms formulated from the SLR PICO question. Supplementary Table 2.2. Search strategy and results from MEDLINE (PubMed) database. Supplementary Table 2.3. Search strategy and results from Science Direct database. Supplementary Table 2.4. Search strategy and results from Scopus database. Supplementary Table 2.5. Search strategy and results from Cochrane Library database. [file 13643_2024_2520_MOESM2_ESM.pdf]

SLR QUESTION

In Asian breast cancer patients **[P]**, how does personalised and precision medicine (in terms of breast cancer molecular subtypes diagnosis, presence or absence of biomarkers and genetic variants **[IC]** affect breast cancer treatment response or outcome **[O]**?

Supplementary Table 2.1: The keywords and search terms formulated from the SLR PICO question.

PICO ELEMENTS		KEYWORDS	SEARCH TERMS
**P**opulation<br><br>*Asian breast cancer patients*	**P**opulation 1	Asian	Asian<br>Asia
	**P**opulation 2	Breast cancer	Breast cancer
**I**ntervention and **C**omparison<br><br>*personalised and precision medicine (in terms of breast cancer molecular subtypes diagnosis, presence or absence of biomarkers and genetic variants*		Personalised medicine	Personalised medicine<br>Personalised therapy<br>Personalised treatment
		Precision medicine	Precision medicine<br>Precision therapy<br>Precision treatment
		Chemotherapy	Chemotherapy
		Adjuvant chemotherapy<br>Neoadjuvant chemotherapy	Adjuvant chemotherapy<br>Neoadjuvant chemotherapy
		Targeted therapy	Targeted therapy<br>Targeted treatment
		Molecular subtype	Molecular subtype<br>Breast cancer subtype<br>Luminal breast cancer<br>HER2 enriched breast cancer<br>Triple negative breast cancer

		Immunohistochemistry marker	Immunohistochemistry marker<br>Breast cancer immunohistochemistry marker<br>Breast cancer marker<br>Estrogen receptor, ER breast cancer<br>Progesterone receptor, PR breast cancer<br>Hormone receptor, HR breast cancer<br>Human epidermal growth factor receptor 2, HER2 breast cancer<br>Ki-67 proliferation index<br>Ki67 proliferation index
		Genetic variant	Genetic variant<br>Genetic mutation<br>Genetic polymorphism
**O**utcome<br><br>*breast cancer treatment response or outcome*	**O**utcome 1	Treatment response	Treatment response<br>Treatment efficacy<br>Pathological response<br>Pathological complete response<br>Residual cancer burden
	**O**utcome 2	Survival	Overall survival<br>Disease free survival<br>Recurrence free survival

Supplementary Table 2.2: Search strategy and results from MEDLINE (PubMed) database.

Domains	Search Code	Terms Search:	Results
Populations (P) – P1	#1	Asian	184,073
	#2	Asia	771,458
	#3	Asian OR Asia<br>**#1 OR #2**	883,846
Populations (P) – P2	#4	"breast cancer"	242,900
	#5	"breast cancer"[MeSH Terms]	219,798
	#6	"breast cancer" OR "breast cancer"[MeSH Terms]<br>**#4 OR #5**	294,715
Intervention and Comparison (IC)	#7	Personalised medicine	67,924
	#8	Personalised therapy	3,397,587
	#9	Personalised treatment	3,687,108
	#10	**#7 OR #8 OR #9**	3,742,766
	#11	Precision medicine	65,058
	#12	Precision therapy	103,227
	#13	Precision treatment	124,900
	#14	**#11 OR #12 OR #13**	149,522
	#15	Chemotherapy	2,399,091
	#16	Adjuvant chemotherapy	65,228
	#17	Neoadjuvant chemotherapy	40,389
	#18	**#16 OR #17**	90,810
	#19	Targeted therapy	646,391
	#20	Targeted treatment	774,859
	#21	**#19 OR #20**	792,005
	#22	Molecular subtype	52,526
	#23	Breast cancer subtype	15,131
	#24	Luminal breast cancer	6,087
	#25	HER2 enriched breast cancer	1,062
	#26	Triple negative breast cancer	16,036
	#27	**#22 OR #23 OR #24 OR #25 OR #26**	73,880
	#28	Immunohistochemistry marker	72,652
	#29	Breast cancer immunohistochemistry marker	5,824
	#30	Breast cancer marker	24,504
	#31	Estrogen receptor, ER breast cancer	26,033
	#32	Progesterone receptor, PR breast cancer	11,773
	#33	Hormone receptor, HR breast cancer	3,045
	#34	Human epidermal growth factor receptor 2, HER2 breast cancer	11,369
	#35	Ki-67 proliferation index	4,778

Domains	Search Code	Terms Search:	Results
	#36	Ki67 proliferation index	5,153
	#37	Ki-67 proliferation index OR Ki67 proliferation index	5,661
	#38	**#28 OR #29 OR #30 OR #31 OR #32 OR #33 OR #34 OR #37**	126,445
	#39	Genetic variant	211,003
	#40	Genetic mutation	682,297
	#41	Genetic polymorphism	265,401
	#42	**#39 OR #40 OR #41**	986,135
	#43	#10 OR #14 OR #15 OR #18 OR #21 OR #27 OR #38 OR #42	6,292,695
Outcome (O) – O1	#44	Treatment response	983,004
	#45	Treatment efficacy	1,535,121
	#46	Pathological response	390,054
	#47	Pathological complete response	45,022
	#48	Residual cancer burden	1,625
	#49	**#44 OR #45 OR #46 OR #47 OR #48**	2,441,702
Outcome (O) – O2	#50	Overall survival	1,746,391
	#51	Disease free survival	140,882
	#52	Recurrence free survival	57,414
	#53	**#50 OR #51 OR #52**	1,746,391
Populations (P), Interventions –Comparator (IC), and Outcome (O)	#54	**#3 AND #6 AND #43 AND #49**	2,122
	P1, P2, IC, O1	**Filter: English**	**2,100**
	#55	**#3 AND #6 AND #43 AND #53**	2,680
	P1, P2, IC, O2	**Filter: English**	**2,657**

Note: All searched articles range from 01/01/2000 - 31/03/2021 and search results #54 and #55 are uploaded to Mendeley to remove any duplicates.

Supplementary Table 2.3: Search strategy and results from Science Direct database.

Domains	Search Code	Terms Search:	Results
Populations (P) – P1	#1	Asia	687,698
	#2	Asian	687,698
	#3	Asian OR Asia	687,698
Populations (P) – P2	#4	"breast cancer"	324,494
Intervention and Comparison (IC)	#5	Personalised medicine	9,843
	#6	Precision therapy	107,536
	#7	Neoadjuvant chemotherapy	53,753
	#8	Targeted therapy	924,176
	#9	Molecular subtypes	193,570
Outcome (O) – O1	#10	Pathological complete response	238,669
	#11	Residual cancer burden	31,307
Outcome (O) – O2	#12	Overall survival	28,338
	#13	Disease free survival	15,688
Populations (P), Interventions – Comparator (IC), and Outcome (O)	#14 P1, P2, IC, O1	**#3 AND #4 OR #5 OR #6 OR #7 OR #8 OR #9 AND #10 OR #11 Filter: English Journals; Annals of oncology, the breast and the Lancet.**	**2,331**
	#15 P1, P2, IC, O2	**#3 AND #4 OR #5 OR #6 OR #7 OR #8 OR #9 AND #12 OR #13 Filter: English. Journals; Annals of oncology, the breast and the Lancet.**	**651**

Note: All searched articles range from 01/01/2000 - 31/03/2021 and search results #14 and #15 are uploaded to Mendeley to remove any duplicates.

Supplementary Table 2.4: Search strategy and results from Scopus database.

Domains	Search Code	Terms Search:	Results
Populations (P), Interventions –Comparator (IC), and Outcome (O)	#1 P1, P2, IC, O1	**Asian OR asia OR "breast cancer" AND "Breast cancer" AND "personalised medicine" OR "personalised treatment" OR "precision therapy" OR "precision medicine" OR "precision treatment" OR chemotherapy OR "neoadjuvant chemotherapy" OR "adjuvant chemotherapy" OR "targeted therapy" OR "targeted treatment" OR "molecular subtypes" OR "breast cancer subtypes" OR "luminal breast cancer" OR "HER2 enriched" OR "Triple negative breast cancer" OR "Ki-67 proliferation index" OR "Ki67 proliferation index" OR "Human epidermal growth factor 2" OR her2 OR "hormone receptor" , AND hr AND receptor OR "Progesterone receptor" , AND pr AND receptor OR "Estrogen Receptor" , AND er AND receptor OR "Breast cancer markers" OR "breast cancer immunohistochemistry marker" OR "immunohistochemistry marker" OR "genetic variant" OR "genetic mutation" OR "genetic polymorphism" AND "treatment response" OR "treatment efficacy" OR "Pathological response" OR "Pathological complete response" OR "Residual cancer burden"**<br>**Filter: English**	**283**
	#2 P1, P2, IC, O2	**Asian OR Asia OR "breast cancer" AND "Breast cancer" AND "personalised medicine" OR "personalised treatment" OR "precision therapy" OR "precision medicine" OR "precision treatment" OR chemotherapy OR "neoadjuvant chemotherapy" OR "adjuvant chemotherapy" OR "targeted therapy" OR "targeted treatment" OR "molecular subtypes" OR "breast cancer subtypes" OR "luminal breast cancer" OR "HER2 enriched" OR "Triple negative breast cancer" OR "Ki-67 proliferation index" OR "Ki67 proliferation index" OR "Human epidermal growth factor 2" OR HER2 OR "hormone receptor", HR receptor OR "Progesterone receptor", PR receptor OR "Estrogen Receptor", ER receptor OR "Breast cancer markers" OR "breast cancer immunohistochemistry marker" OR "immunohistochemistry marker" OR "genetic variant" OR "genetic mutation" OR "genetic polymorphism" AND "recurrence free survival" OR "disease free survival" OR "overall survival"**<br>**Filter: English**	**909**

Note: All searched articles range from 01/01/2000 - 31/03/2021 and search results #1 and #2 are uploaded to Mendeley to remove any duplicates.

Supplementary Table 2.5: Search strategy and results from Cochrane Library database.

Domains	Search Code	Terms Search:	Results
Populations (P) – P1	#1	Asian	9,875
	#2	Asia	4,684
	#3	**#1 OR #2**	13,716
Populations (P)-P2	#4	"breast cancer"	32,751
	#5	Personalised medicine	91,971
Intervention and Comparison (IC)	#6	Personalised therapy	34,144
	#7	Personalised treatment	38,379
	#8	**#5 OR #7 OR #8**	49,584
	#9	Precision medicine	1,446
	#10	Precision therapy	3,900
	#11	Precision treatment	4,763
	#12	**#9 OR #10 OR #11**	6,096
	#13	Chemotherapy	66,893
	#14	Adjuvant chemotherapy	14,435
	#15	Neoadjuvant chemotherapy	6,746
	#16	**#13 OR #14 OR 16**	66,893
	#17	Targeted therapy	48,897
	#18	Targeted treatment	53,514
	#19	**#17 OR #18**	65,427
	#20	Molecular subtype	738
	#21	Breast cancer subtype	927
	#22	Luminal breast cancer	514
	#23	HER2 enriched breast cancer	153
	#24	Triple negative breast cancer	1,732
	#25	**#20 OR #21 OR #22 OR #23 OR #24**	1,543
	#26	Immunohistochemistry marker	1,287
	#27	Breast cancer immunohistochemistry marker	288
	#28	Breast cancer marker	2,211
	#29	Estrogen receptor, ER breast cancer	1,399
	#30	Progesterone receptor, PR breast cancer	300
	#31	Hormone receptor, HR breast cancer	1,583
	#32	Human epidermal growth factor receptor 2, HER2 breast cancer	1,869
	#33	Ki-67 proliferation index	210
	#34	Ki67 proliferation index	173
	#35	Ki-67 proliferation index OR Ki67 proliferation index	327
	#36	**#26 OR #27 OR #28 OR #29 OR #30 OR #31 OR #32 OR #35**	7,318

Domains	Search Code	Terms Search:	Results
	#37	Genetic variant	3,767
	#38	Genetic mutation	5,783
	#39	Genetic polymorphism	1,986
	#40	**#37 OR #38 OR #39**	13,285
	#41	#8 OR #12 OR #16 OR #19 OR #25 OR #36 OR #40	516
Outcome (O) – O1	#42	Treatment response	152,740
	#43	Treatment efficacy	231,378
	#44	Pathological response	16,632
	#45	Pathological complete response	5,684
	#46	Residual cancer burden	297
	#47	**#42 OR #43 OR #44 OR #45 OR #46**	320,954
Outcome (O) – O2	#48	Overall survival	51,275
	#49	Disease free survival	34,446
	#50	Recurrence free survival	14,722
	#51	**#48 OR #49 OR #50**	62,081
Populations (P), Interventions – Comparator (IC), and Outcome (O)	#52 P1, P2, IC, O1	**#3 AND #6 AND #43 AND #49 Filter: English**	**21**
	#53 P1, P2, IC, O2	**#3 AND #6 AND #43 AND #53 Filter: English**	**11**

Note: All searched articles range from 01/01/2000 - 31/03/2021 and search results #52 and #53 are uploaded to Mendeley to remove any duplicates.
