# Supplementary material for: Exploring the effectiveness of molecular subtypes, biomarkers, and genetic variations as first-line treatment predictors in Asian breast cancer patients: a systematic review and meta-analysis
Source: Syst Rev. 2024 Apr 4;13:100. doi: 10.1186/s13643-024-02520-5 (PMC10993489; doi:10.1186/s13643-024-02520-5)
Supplement: Supplementary file 5 — Additional file 5. Modified Newcastle-Ottawa Quality Assessment Scale. [file 13643_2024_2520_MOESM5_ESM.pdf]

MODIFIED NEWCASTLE - OTTAWA QUALITY ASSESSMENT SCALE
CASE-CONTROL STUDIES

Note: A study can be awarded a maximum of one star (*) for each numbered item within the Selection and Exposure categories. A maximum of two stars can be given for Comparability if additional factor is included.

**Study No**		
**Author, year**		
**Criterion**	**Decision Rule**	**Score (*=1, no*=0)**
**SELECTION**		
1. Is the case definition adequate?	a) Yes, with independent validation *<br>*(Histopathologically confirmed Asian breast cancer patients receiving medically prescribed/planned treatment for breast cancer)*<br>b) Yes, based on self-reports.<br>c) No description.	
2. Representativeness of the cases	a) Consecutive or obviously representative series of cases*<br>*(Asian breast cancer patients with molecular subtypes and/or somatic mutations or polymorphisms receiving targeted chemotherapy with pCR and/or RCB outcome)*<br>b) Not satisfying requirements in part (a), or not stated.	
3. Selection of controls	a) Controls were selected from the same source population as the cases *<br>*(Hospital controls: Asian breast cancer patients with molecular subtypes and/or somatic mutations or polymorphisms receiving placebo chemotherapy with pCR and/or RCB outcome)*<br>b) Controls were selected from a different source population *(Community controls i.e., healthy controls)*.<br>c) No description.	
4. Definition of controls	a) If cases are first occurrence of breast cancer (no previous history of disease), then it must explicitly state that controls have no history of this outcome. If cases have new recurrence of breast cancer, then controls with previous occurrences of outcome of interest should not be excluded*<br>b) No description of breast cancer and treatment	
**COMPARABILITY**		
1. Comparability of cases and controls on the basis of the design or analysis	a) Study controls for breast cancer treatment with pCR and/or RCB outcome*<br>b) Study controls for breast cancer patients' characteristics (molecular subtypes / biomarkers / genetic variations / differential expression) with pCR and/or RCB outcome*<br>*Note: Cases and controls must be matched in the design and/or confounders must be adjusted for in the analysis. Alone statements of no differences between groups or that differences were not statistically significant are not sufficient.*	
**EXPOSURE**		
1. Ascertainment of exposure	a) Structured secure record *<br>*(e.g., histopathology laboratory report/results)*<br>b) Structured interview where blinded to case/control status *<br>c) Interview not blinded to case/control status<br>d) Written self-report or medical record (unstructured data) only<br>e) No description	

2. Same method of ascertainment for cases and controls	a) Yes *<br>b) No	
3. Non-response rate	a) Same for both groups *<br>b) Non-respondents described<br>c) Rate different and no designation	
	**SCORE:**	**/ 9**

MODIFIED NEWCASTLE - OTTAWA QUALITY ASSESSMENT SCALE
COHORT STUDIES

Note: A study can be awarded a maximum of one star (*) for each numbered item within the Selection and Outcome categories. A maximum of two stars can be given for Comparability if additional factor is included.

**Study No**		
**Author, year**		
**Criterion**	**Decision Rule**	**Score (*=1, no*=0)**
**SELECTION**		
1. Representativeness of the exposed cohort	a) Consecutive eligible participants were selected, participants were randomly selected, or all participants were invited to participate from the source population *<br>*(Histopathologically confirmed Asian breast cancer patients receiving medically prescribed/planned treatment for breast cancer)*<br>b) Not satisfying requirements in part (a), or not stated	
2. Selection of the non-exposed cohort	a) Selected from the same source population*<br>*(Hospital controls: Asian breast cancer patients with molecular subtypes and/or somatic mutations or polymorphisms receiving placebo chemotherapy with pCR and/or RCB outcome)*<br>b) Selected from a different source population (community controls i.e., healthy controls)<br>c) No description	
3. Ascertainment of exposure	a) Structured secure record *<br>*(e.g., histopathology laboratory report/results)*<br>b) Structured interview *<br>c) Written self-report<br>d) No description	
4. Demonstration that outcome of interest was not present at the start of the study	a) Yes *<br>b) No or not explicitly stated	
**COMPARABILITY**		
1. Comparability of cases and controls on the basis of the design or analysis	a) Study controls for breast cancer treatment with pCR and/or RCB outcome*<br>b) Study controls for breast cancer patients' characteristics (molecular subtypes / biomarkers / genetic variations / differential expression) with pCR and/or RCB outcome*<br>*Note: Exposed and non-exposed individuals must be matched in the design and/or confounders must be adjusted for in the analysis. Alone statements of no differences between groups or that differences were not statistically significant are not sufficient.*	

OUTCOME		
1. Assessment of outcome	a) Independent or blind assessment stated, or confirmation of the outcome by reference to secure records * *(e.g., histopathology laboratory report/results)*<br>b) Record linkage (e.g., identified through ICD codes on database records) *<br>c) Self-report with no reference to original structured histopathology data or imaging<br>d) No description	
2. Was follow-up long enough for outcomes to occur?	a) Yes (≥2 months) *<br>b) No (<2 months)	
3. Adequacy of follow up of cohorts	a) Complete follow up – all participants accounted for *<br>b) Subjects lost to follow up unlikely to introduce bias (<15% lost to follow up, or description provided of those lost*)<br>c) Follow up rate <85% and no description of those lost provided<br>d) No statement	
	**SCORE:**	**/ 9**
