# Supplementary material for: Exploring the effectiveness of molecular subtypes, biomarkers, and genetic variations as first-line treatment predictors in Asian breast cancer patients: a systematic review and meta-analysis
Source: Syst Rev. 2024 Apr 4;13:100. doi: 10.1186/s13643-024-02520-5 (PMC10993489; doi:10.1186/s13643-024-02520-5)
Supplement: Supplementary file 6 — Additional file 6. Quality of the included studies. Supplementary Table 6.1. Newcastle-Ottawa Scale of each included cohort study. Supplementary Figure 6.1. Risk of bias by domain and question in 87 cohort studies using Newcastle-Ottawa Scale. Numbers on the green bar represent the number of studies with low risk of bias over the number of studies assessed. Supplementary Table 6.2. Newcastle-Ottawa Scale of each included case-control study. Supplementary Figure 6.2. Risk of bias by domain and question in 14 case-cohort studies using Newcastle-Ottawa Scale. Numbers on the green bar represent the number of studies with low risk of bias over the number of studies assessed. [file 13643_2024_2520_MOESM6_ESM.pdf]

Supplementary Table 6.1. Newcastle-Ottawa Scale of each included cohort study.

Study ID	Author, year	PubMed ID	Selection				Comparability		Outcome			Total quality score (out of 9)
			Representative-ness of the exposed cohort	Selection of non-exposed cohort	Ascertainment of exposure	Outcome of interest not present at start	pCR and breast cancer treatment	pCR and breast cancer characterisa tion	Assessment of outcome	Follow-up long enough	Adequacy of follow-up	
1	Chen XS et al., 2010b	20372832	*		*	*		*	*	*	*	7
2	Li K et al., 2020	32638235	*	*	*	*	*	*	*	*	*	9
4	Mohammed AA et al., 2020	32102539	*		*	*		*	*	*	*	7
5	Tewari M et al., 2010	21377101	*	*	*	*	*	*	*	*	*	9
6	Prajoko YW et al., 2014	24568463	*		*	*			*	*	*	6
8	Lim LY et al., 2017	28000426	*		*	*		*	*	*	*	7
10	Fei F et al., 2013	22868245	*		*	*		*	*	*	*	7
11	Salim DK et al., 2014	25422228	*		*	*		*	*	*	*	7
12	Park JH et al., 2016	27491481	*		*	*		*	*	*		6
13	Elnemr GM et al., 2016	26925684	*		*	*		*	*	*	*	7
14	Wang YJ et al., 2015	26107211	*		*	*		*	*	*	*	7
15	Sugitani I et al., 2017	28547525	*		*	*		*	*	*	*	7
16	Lv M et al., 2011	22296393	*		*	*		*	*		*	6
17	Elsamany S et al., 2015	26625828	*		*	*		*	*	*	*	7
18	Duman BB et al., 2012	23098527	*	*	*	*	*	*	*	*	*	9
19	Elsamany S et al., 2016	27644666	*		*	*		*	*	*	*	7

Supplementary Table 6.1*(cont.)*

Study ID	Author, year	PubMed ID	Selection				Comparability		Outcome			Total quality score (out of 9)
			Representative-ness of the exposed cohort	Selection of non-exposed cohort	Ascertainment of exposure	Outcome of interest not present at start	pCR and breast cancer treatment	pCR and breast cancer characterisation	Assessment of outcome	Follow-up long enough	Adequacy of follow-up	
20	Ding J et al., 2017	28915617	*		*	*		*	*	*	*	7
21	Hong J et al., 2020	33123282	*		*	*		*	*	*	*	7
22	Lv Y et al., 2020	32513358	*		*	*		*	*	*	*	7
24	Kong Y et al., 2019	31015393	*		*	*		*	*	*	*	7
25	Wang LC et al., 2020	32508292	*		*	*		*	*	*	*	7
27	Bi Z et al., 2020	32776291	*	*	*	*	*	*	*	*	*	9
28	Shan BJ et al., 2020	32953611	*	*	*	*	*	*	*	*	*	9
30	Xu Y et al., 2020	32782487	*		*	*		*	*	*	*	7
31	Zhang GC et al., 2012	22547076	*	*	*	*	*	*	*	*	*	9
32	Lei C et al., 2020	32140269	*		*	*		*	*	*	*	7
33	Liu Y et al., 2019	30655846	*		*	*		*	*	*	*	7
34	Li S et al., 2018	30275685	*		*	*		*	*	*	*	7
35	Xuhong J et al., 2020	33000490	*		*	*		*	*	*	*	7
36	Yan H et al., 2020	33328766	*		*	*		*	*	*	*	7
37	Yao L et al., 2011	21196440	*	*	*	*	*	*	*	*	*	9
38	Yang L et al., 2019	30776175	*		*	*		*	*	*	*	7

Supplementary Table 6.1*(cont.)*

Study ID	Author, year	PubMed ID	Selection				Comparability		Outcome			Total quality score (out of 9)
			Representative-ness of the exposed cohort	Selection of non-exposed cohort	Ascertainment of exposure	Outcome of interest not present at start	pCR and breast cancer treatment	pCR and breast cancer characterisation	Assessment of outcome	Follow-up long enough	Adequacy of follow-up	
39	Man VC et al., 2017	28484080	*		*	*		*	*	*	*	7
41	Wu J et al., 2011	21830158	*	*	*	*	*	*	*	*	*	9
43	Nie C et al., 2018	30572455	*		*	*		*	*	*	*	7
44	Wu X et al., 2018	29483583	*	*	*	*	*	*	*	*	*	9
45	Chen W et al., 2015	25789069	*	*	*	*	*	*	*	*	*	9
46	Chen X et al., 2016	28174484	*	*	*		*		*	*	*	7
47	Mou P et al., 2018	30552707	*			*	*	*		*	*	6
48	Xie F et al., 2019	30977429	*	*	*	*	*	*	*	*	*	9
49	Wang Y et al., 2016	26238069	*	*	*	*	*	*	*	*	*	9
50	Liu S et al., 2016	26526577	*		*	*	*	*	*	*	*	8
51	Zhou L et al., 2017	29108309	*		*	*		*	*	*	*	7
52	Kong DD et al., 2020	32724406	*		*	*		*	*	*	*	7
53	Yu KD et al., 2019	30892700	*	*	*	*	*	*	*	*	*	9
54	Wang J et al., 2016	27149453	*	*	*	*	*	*	*	*	*	9
55	Chen R et al., 2018	29480449	*		*	*		*	*	*	*	7
56	Lv M et al., 2020	33447559	*	*	*	*	*	*	*	*	*	9

Supplementary Table 6.1*(cont.)*

Study ID	Author, year	PubMed ID	Selection				Comparability		Outcome			Total quality score (out of 9)
			Representative-ness of the exposed cohort	Selection of non-exposed cohort	Ascertainment of exposure	Outcome of interest not present at start	pCR and breast cancer treatment	pCR and breast cancer characterisation	Assessment of outcome	Follow-up long enough	Adequacy of follow-up	
57	Bi Z et al., 2019	30666563	*	*		*	*	*		*	*	7
58	Wang X et al., 2019	31490377	*	*	*	*	*	*	*	*	*	9
59	Zhao YC et al., 2014	24317109	*		*	*		*	*	*	*	7
61	Yin Y et al., 2013	23592141	*	*	*	*	*	*	*	*	*	9
62	Li ZH et al., 2013	23681778	*	*	*	*	*	*	*	*	*	9
63	Wang Y et al., 2018	29963107	*		*	*		*	*	*	*	7
64	Yuan H et al., 2015	25979484	*	*	*	*	*	*	*	*	*	9
65	Chiu JW et al., 2019	31004045	*	*	*	*	*	*	*	*	*	9
66	Zhang Z et al., 2014	24977004	*		*	*		*	*	*	*	7
67	Tan QX et al., 2014	25400769	*		*	*		*	*	*	*	7
68	Li M et al., 2016	26956035	*	*	*	*	*	*	*	*	*	9
69	Wang YY et al., 2016	27608899	*	*	*	*	*	*	*	*	*	9
70	Yao L et al., 2015	26752929	*		*	*		*	*	*	*	7
71	Chen S et al., 2013	23473851	*		*	*			*	*	*	6
72	Luo J et al., 2016	26937935	*	*	*	*	*		*	*	*	8
73	Wang K et al., 2013	22976542	*		*	*		*	*	*	*	7

Supplementary Table 6.1*(cont.)*

Study ID	Author, year	PubMed ID	Selection				Comparability		Outcome			Total quality score (out of 9)
			Representative-ness of the exposed cohort	Selection of non-exposed cohort	Ascertainment of exposure	Outcome of interest not present at start	pCR and breast cancer treatment	pCR and breast cancer characterisation	Assessment of outcome	Follow-up long enough	Adequacy of follow-up	
74	Zhang W et al., 2017	29057208	*	*	*	*	*	*	*	*	*	9
75	Chen S et al., 2015	26053183	*		*	*		*	*	*	*	7
76	Li XR et al., 2011a	20872186	*		*	*		*	*	*	*	7
77	Kong D et al., 2017	28099918	*		*	*		*	*	*	*	7
78	Li XR et al., 2011b	21080107	*		*	*		*	*	*	*	7
79	Liu YX et al., 2018	29844843	*	*		*		*	*	*	*	7
80	Xu Y et al., 2018	29047188	*		*	*		*		*	*	6
81	Gu X et al., 2015	25480315	*		*	*			*	*	*	6
82	Wan F et al., 2013	23696593	*		*	*		*	*	*	*	7
83	Chen YZ et al., 2012	22903535	*		*	*		*	*	*	*	7
86	Wu Z et al., 2018	29497312	*			*		*	*	*	*	6
88	Meng XL et al., 2012	22480216	*		*	*		*	*	*	*	7
89	Chen XS et al., 2010a	20211870	*		*	*		*	*	*	*	7
90	Wang Z et al., 2021	33552956	*		*	*		*	*	*	*	7
91	Huang O et al., 2009	19845944	*		*	*		*	*	*	*	7
92	Zhu T et al., 2015	26936755	*		*	*		*	*	*	*	7

Supplementary Table 6.1*(cont.)*

Study ID	Author, year	PubMed ID	Selection				Comparability		Outcome			Total quality score (out of 9)
			Representative-ness of the exposed cohort	Selection of non-exposed cohort	Ascertainment of exposure	Outcome of interest not present at start	pCR and breast cancer treatment	pCR and breast cancer characterisation	Assessment of outcome	Follow-up long enough	Adequacy of follow-up	
94	Zhang S et al., 2012	21380781	*		*	*		*	*	*	*	7
95	Zheng Y et al., 2015	26078798	*		*	*		*	*	*	*	7
97	Liu F et al., 2012	22842982	*		*	*		*	*	*	*	7
98	Yu KD et al., 2012	22561335	*		*	*		*	*	*	*	7
99	Liu J et al., 2021	33603855	*		*	*		*	*	*	*	7
100	Ishikawa T et al., 2016	27041672	*	*	*	*	*	*	*	*	*	9
101	Teraoka S et al., 2020	32044511	*		*	*		*	*	*	*	7

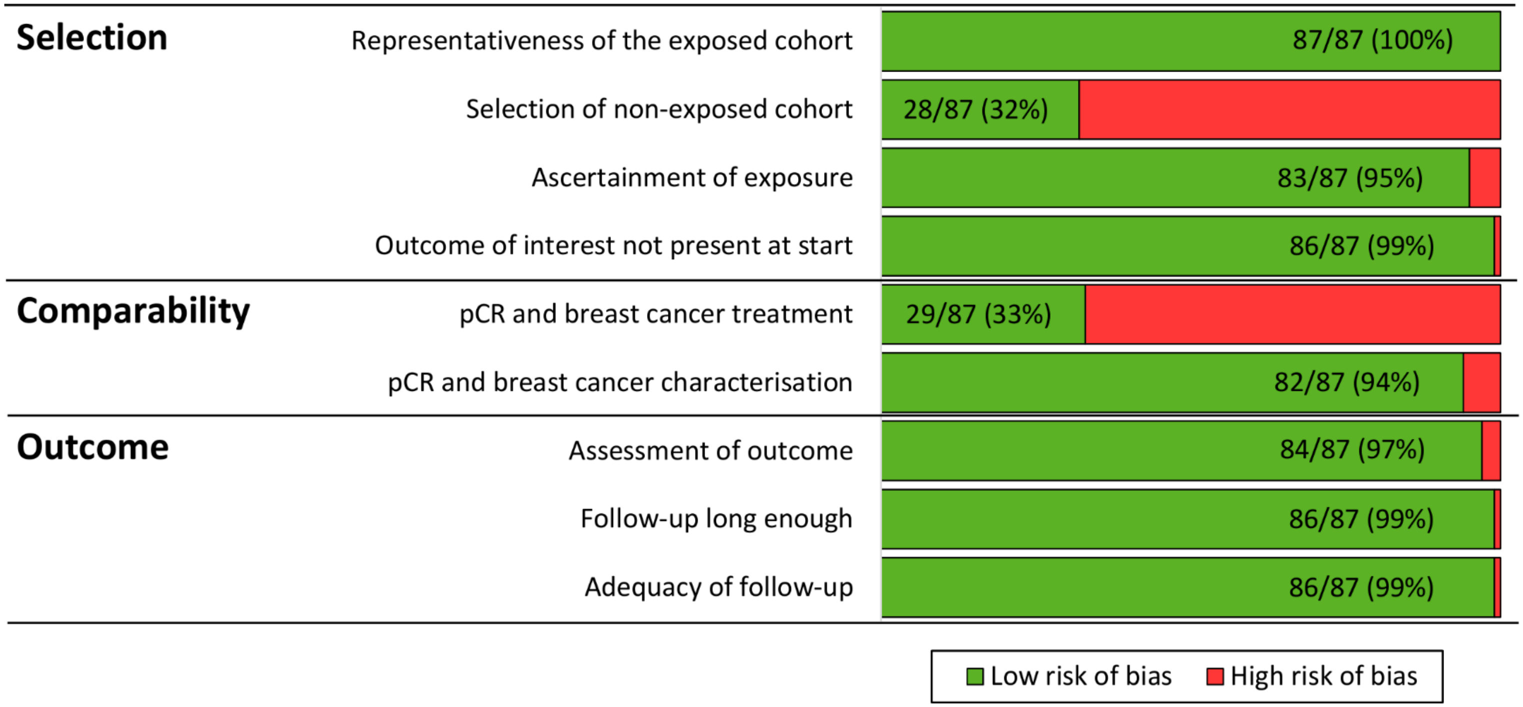

Supplementary Figure 6.1. Risk of bias by domain and question in 87 cohort studies using the Newcastle-Ottawa Scale.

Numbers on the green bar represent the number of studies with low risk of bias over the number of studies assessed.

Supplementary Table6.2. Newcastle-Ottawa Scale of each included case-control study.

**Study ID**	**Author, year**	**PubMed ID**	**Selection**				**Comparability**		**Exposure**			**Total quality score (out of 9)**
			Case definition adequate	Representativeness of the cases	Selection of controls	Definition of controls	pCR and breast cancer treatment	pCR and breast cancer characterisation	Ascertainment of exposure	Cases and controls: same ascertainment method	Cases and controls: same non-response rate	
7	Shao Z et al., 2020	31647503	*	*	*	*	*	*	*	*	*	9
9	Hasegawa Y et al., 2015	26633806	*	*	*	*	*	*	*	*	*	9
23	Chow LWC et al., 2018	29158285	*	*		*		*	*			5
26	Yang M et al., 2019	31939438	*	*	*	*	*	*	*	*	*	9
29	Zhang P et al., 2016	27447966	*	*	*	*	*	*	*	*	*	9
40	Wu Z et al., 2019a	30976202	*	*		*		*	*	*	*	7
42	Wu Z et al., 2019b	30976202	*	*		*		*	*	*	*	7
60	Huang L et al., 2015a	25792830	*	*	*	*	*		*	*	*	8
84	Tang Y et al., 2012	23046633	*	*	*	*	*	*	*	*	*	9
85	Huang L et al., 2015b	26084292	*	*	*	*	*	*	*	*	*	9
87	Wang RX et al., 2017	27885439	*	*	*	*	*	*	*	*	*	9
93	Wang J et al., 2012	22864769	*	*	*	*	*	*	*	*	*	9
96	Wang J et al., 2014	25041784	*	*	*	*	*	*	*	*	*	9
102	Mohammadianpanah M et al., 2012	22002564	*	*	*	*	*	*	*	*	*	9

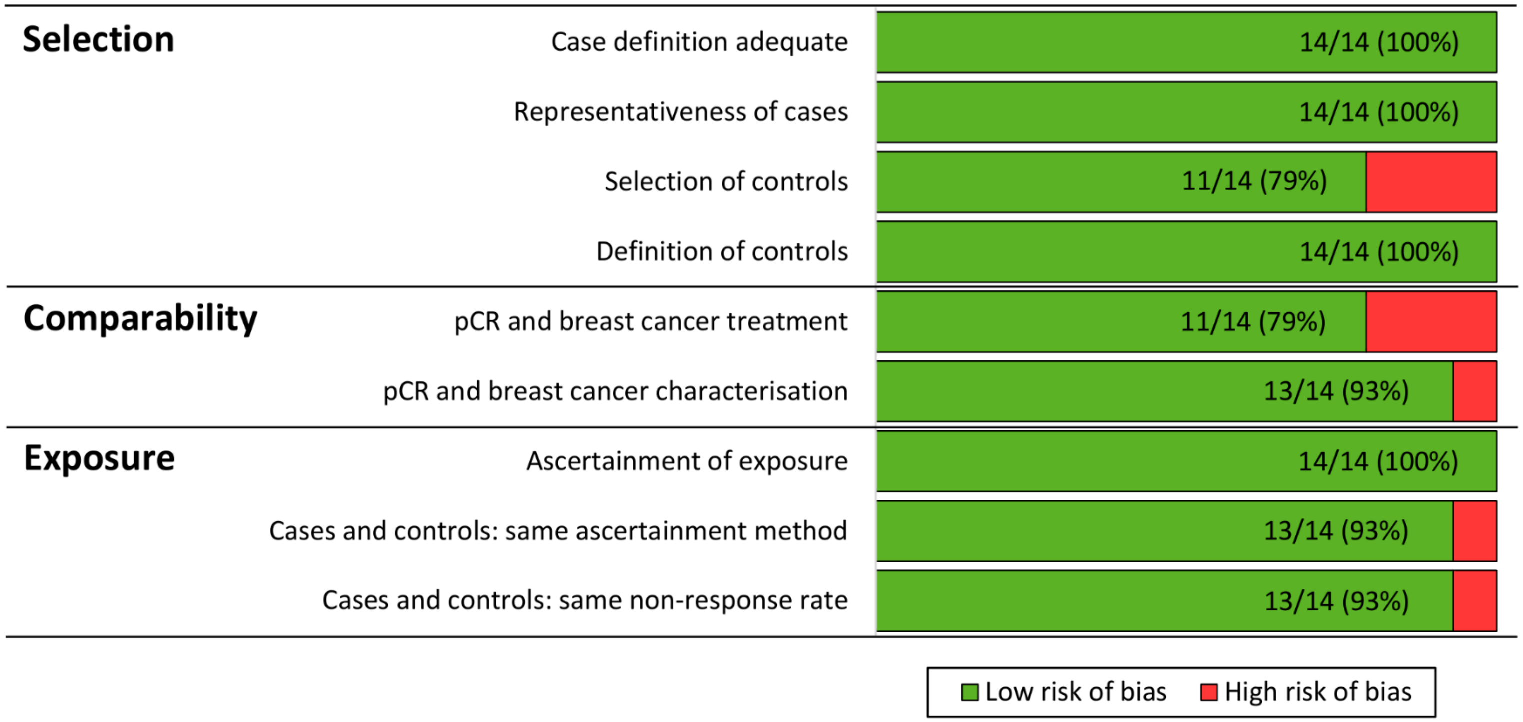

Supplementary Figure 6.2. Risk of bias by domain and question in 14 case-control studies using the Newcastle-Ottawa Scale.

Numbers on the green bar represent the number of studies with low risk of bias over the number of studies assessed.
