# Supplementary material for: Exploring the effectiveness of molecular subtypes, biomarkers, and genetic variations as first-line treatment predictors in Asian breast cancer patients: a systematic review and meta-analysis
Source: Syst Rev. 2024 Apr 4;13:100. doi: 10.1186/s13643-024-02520-5 (PMC10993489; doi:10.1186/s13643-024-02520-5)
Supplement: Supplementary file 7 — Additional file 7. Meta-analysis results. Supplementary Figure 7.1. Pooled pCR outcome of NAC-treated Asian breast cancer patients. Forest plots describing the random effect ORs and 95% CIs from studies assessing the association of pCR outcome in: NAC TA-treated breast cancer patients between (A) TNBC and HER2E; (B) Luminal B and Luminal A; NAC TP-treated breast cancer patients between (C) HER2E and TNBC; NAC TA-treated breast cancer patients with (D) EGFR. I2 and p-value for X2 of heterogeneity are reported for each group analysis. Supplementary Figure 7.2. Pooled reported association of pCR in NAC-treated Asian breast cancer patients presented in different variables. Forest plots describing the random effect ORs and 95% CIs from studies assessing the pooled reported association of pCR in NAC-treated breast cancer patients presented according to (A) Molecular classification; (B) Genetic variations; and (C) Biomarkers. I2 and p-value for X2 of heterogeneity are reported for each group analysis. Supplementary Figure 7.3. Pooled reported association of pCR in NAC-treated Asian breast cancer patients with ER. Forest plot describing the random effect ORs and 95% CIs from studies assessing the association between the biomarker ER and pCR in NAC-treated breast cancer patients. I2 and p-value for X2 of heterogeneity are reported for each group analysis. Supplementary Figure 7.4. Pooled reported association of pCR in NAC-treated Asian breast cancer patients with PR. Forest plot describing the random effect ORs and 95% CIs from studies assessing the association between the biomarker PR and pCR in NAC-treated breast cancer patients. I2 and p-value for X2 of heterogeneity are reported for each group analysis. Supplementary Figure 7.5. Pooled reported association of pCR in NAC-treated Asian breast cancer patients with HR. Forest plot describing the random effect ORs and 95% CIs from studies assessing the association between the hormone receptors (HR) – comprising ER and PR – an [file 13643_2024_2520_MOESM7_ESM.pdf]

META-ANALYSIS RESULTS

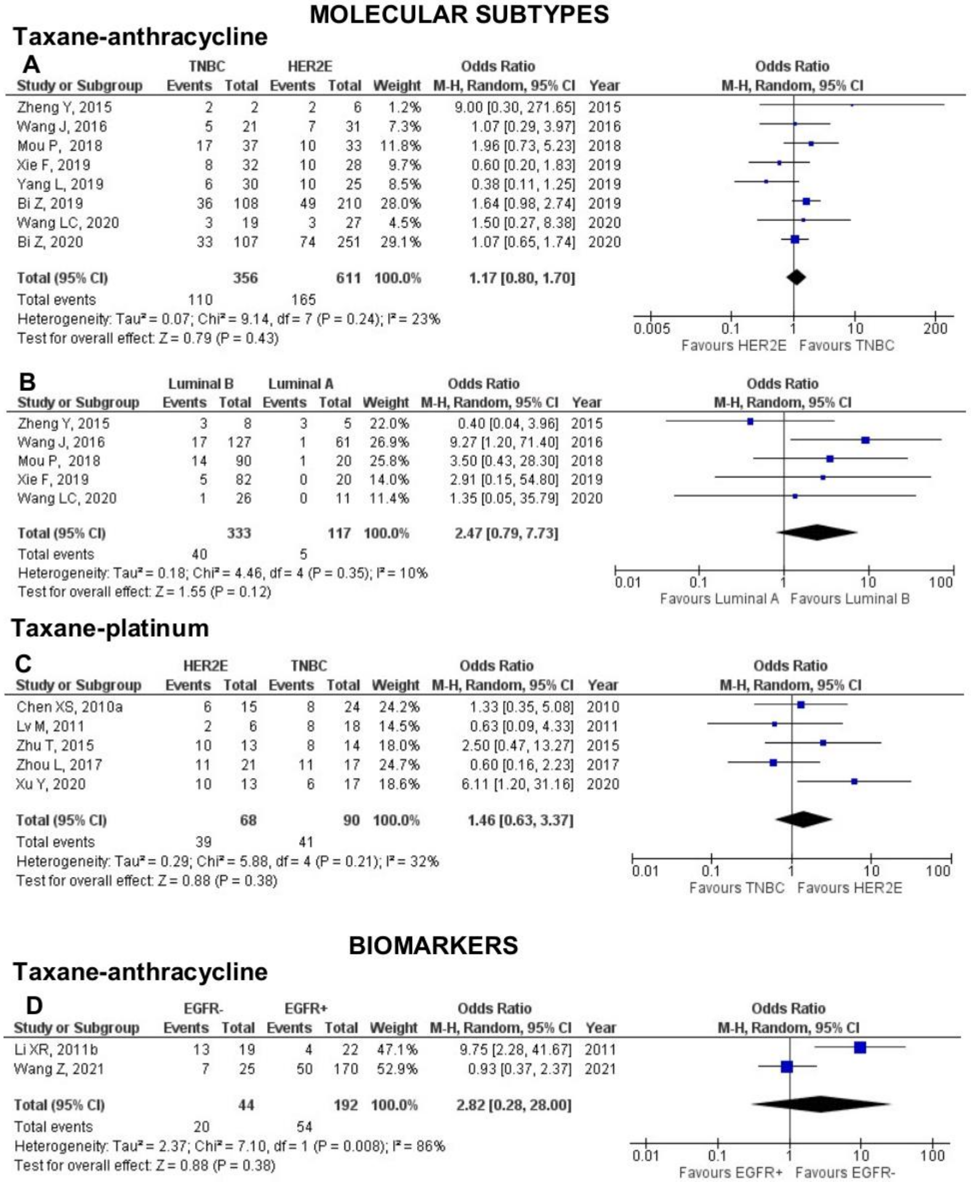

Supplementary Figure 7.1. Pooled pCR outcome of NAC-treated Asian breast cancer patients. Forest plots describing the random effect ORs and 95% CIs from studies assessing the association of pCR outcome in: NAC TA-treated breast cancer patients between (A)TNBC and HER2E; (B) Luminal B and Luminal A; NAC TP-treated breast cancer patients between (C) HER2E and TNBC; NAC TA-treated breast cancer patients with (D) EGFR. I^2 and *p*-value for X^2 of heterogeneity are reported for each group analysis.

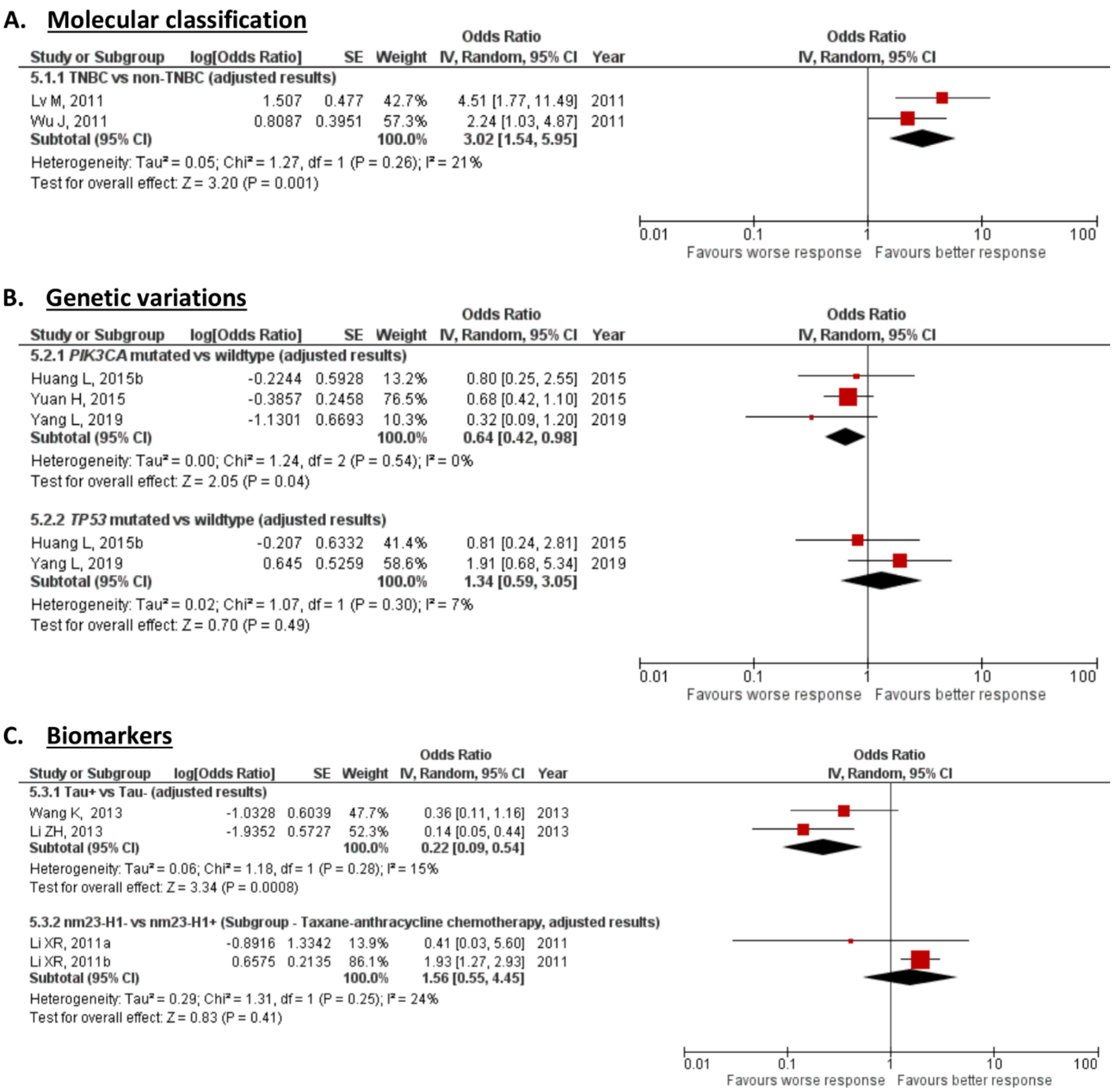

Supplementary Figure 7.2. Pooled reported association of pCR in NAC-treated Asian breast cancer patients presented in different variables. Forest plots describing the random effect ORs and 95% CIs from studies assessing the pooled reported association of pCR in NAC-treated breast cancer patients presented according to (A) Molecular classification; (B) Genetic variations; and (C) Biomarkers. I^2 and *p*-value for X^2 of heterogeneity are reported for each group analysis.

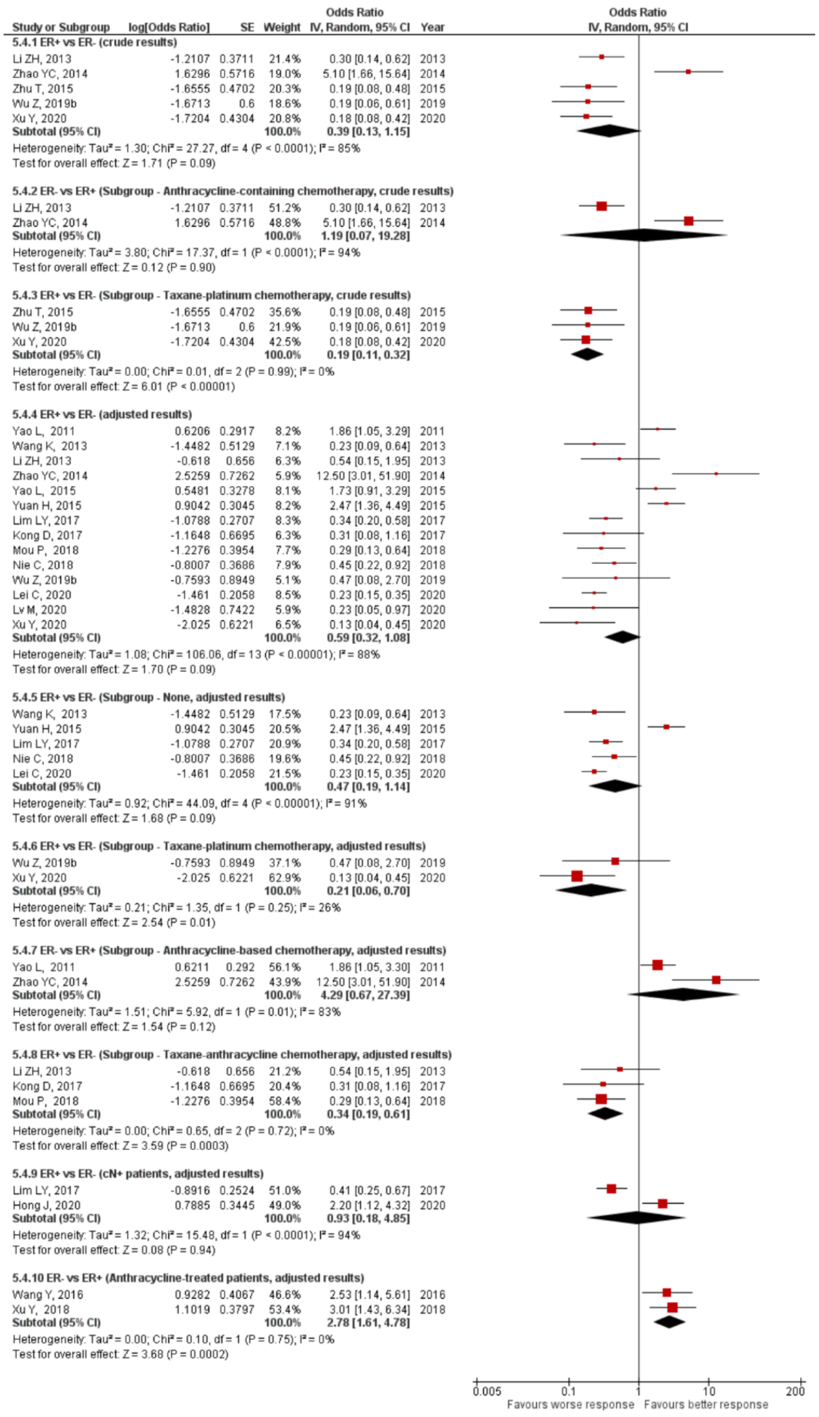

Supplementary Figure 7.3. Pooled reported association of pCR in NAC-treated Asian breast cancer patients with ER. Forest plot describing the random effect ORs and 95% CIs from studies assessing the association between the biomarker ER and pCR in NAC-treated breast cancer patients. I^2 and *p*-value for X^2 of heterogeneity are reported for each group analysis.

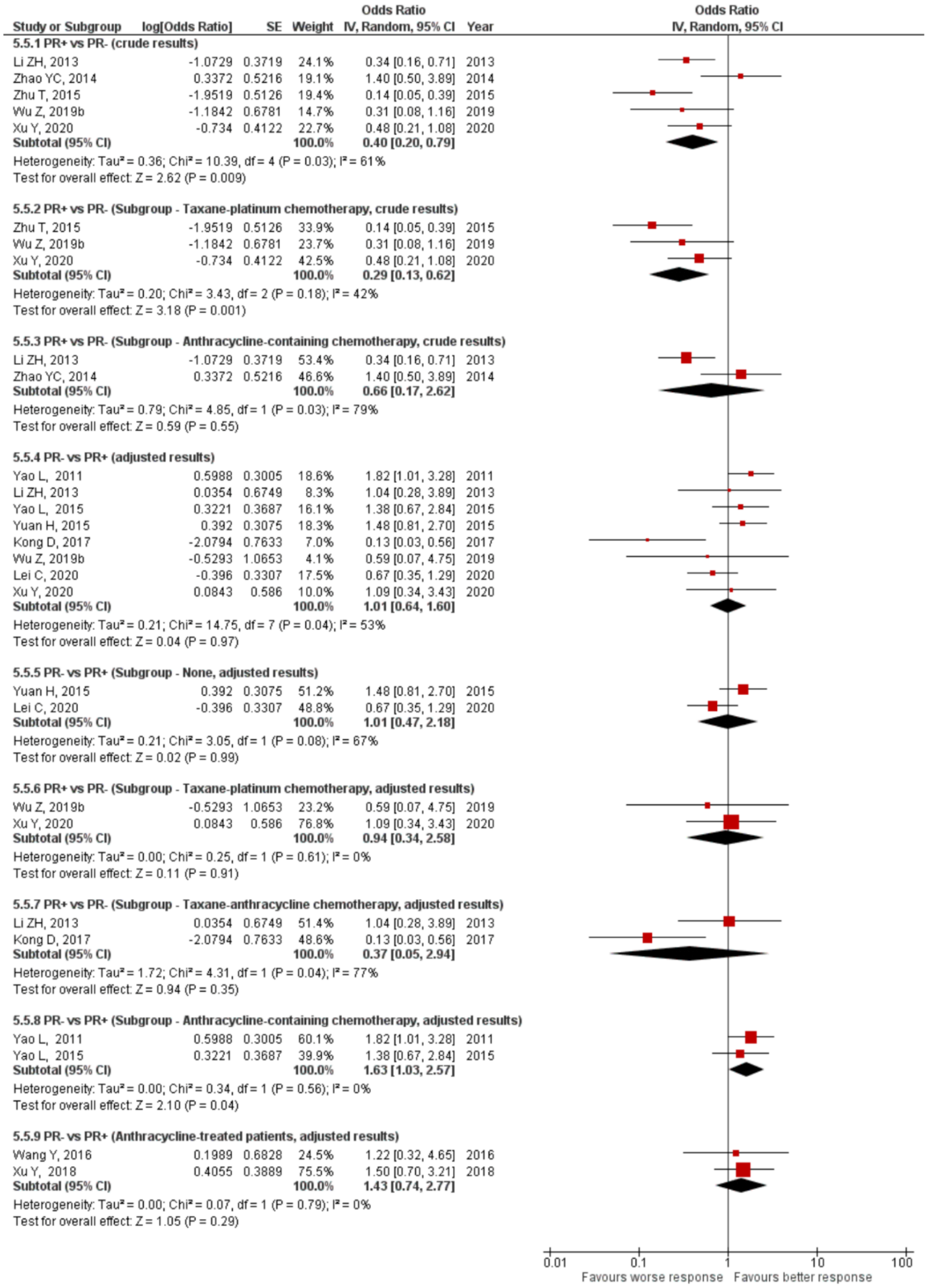

Supplementary Figure 7.4. Pooled reported association of pCR in NAC-treated Asian breast cancer patients with PR. Forest plot describing the random effect ORs and 95% CIs from studies assessing the association between the biomarker PR and pCR in NAC-treated breast cancer patients. I^2 and *p*-value for X^2 of heterogeneity are reported for each group analysis.

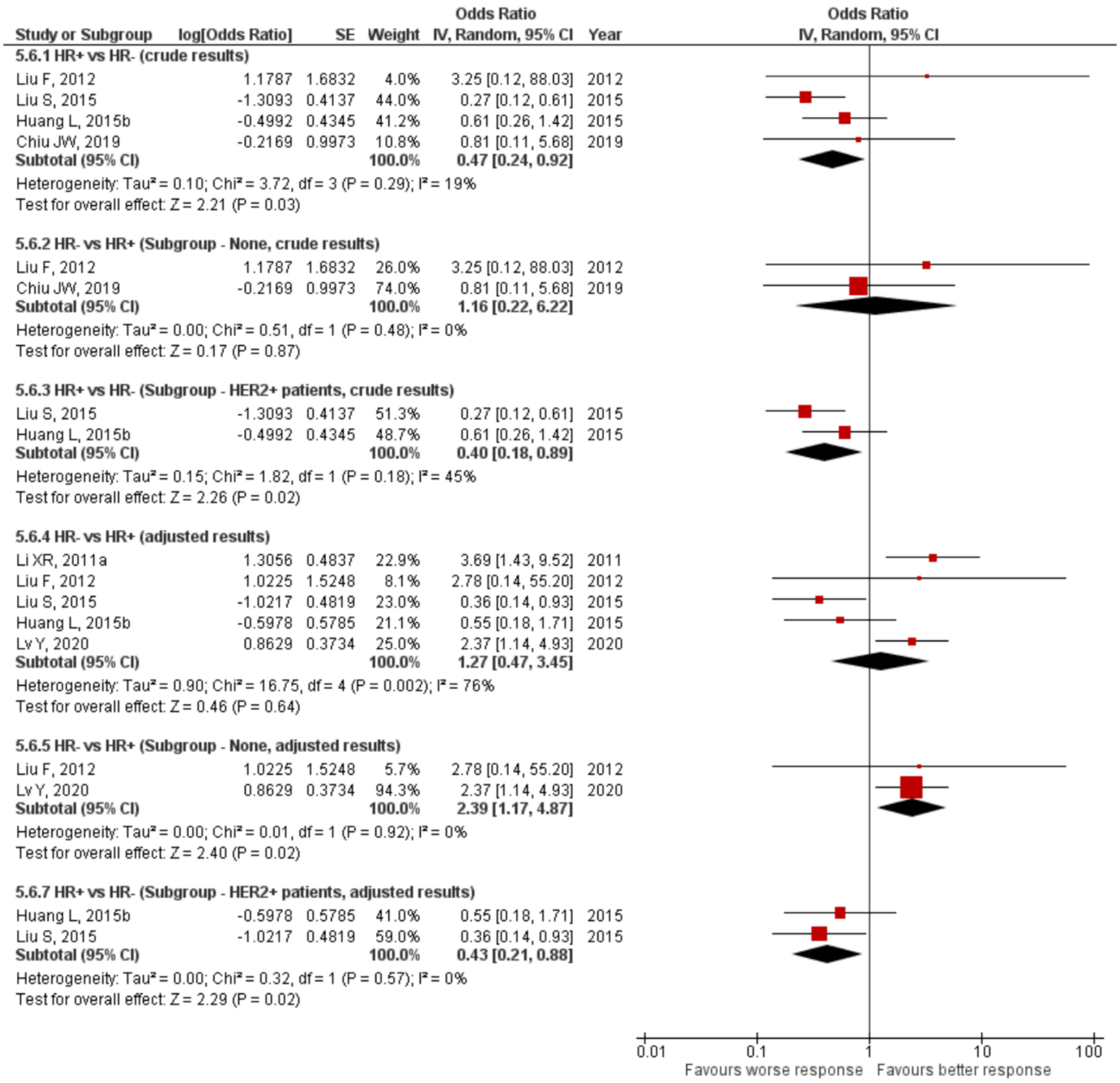

Supplementary Figure 7.5. Pooled reported association of pCR in NAC-treated Asian breast cancer patients with HR. Forest plot describing the random effect ORs and 95% CIs from studies assessing the association between the hormone receptors (HR) – comprising ER and PR – and pCR in NAC-treated breast cancer patients. I^2 and *p*-value for X^2 of heterogeneity are reported for each group analysis.

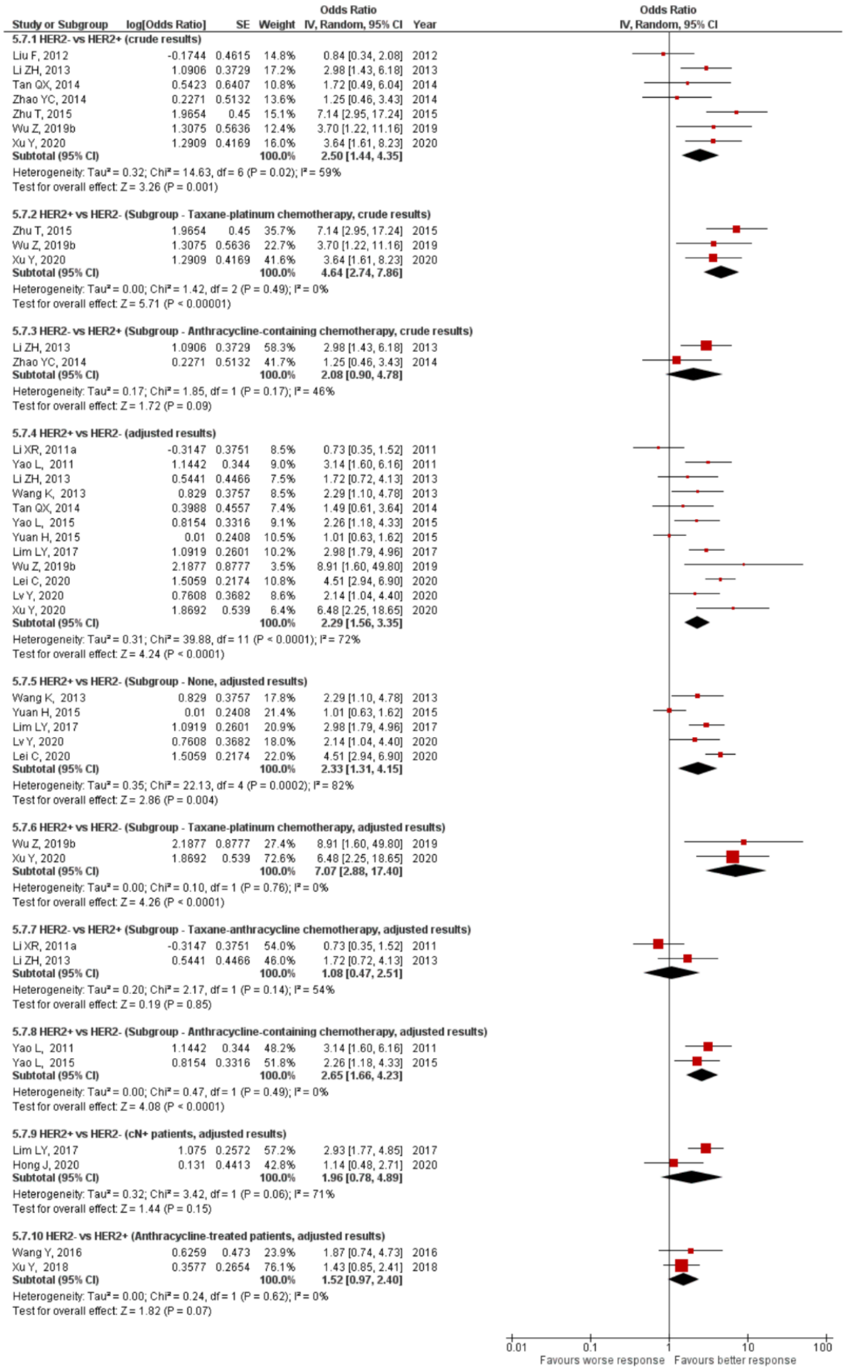

Supplementary Figure 7.6. Pooled reported association of pCR in NAC-treated Asian breast cancer patients with HER2. Forest plot describing the random effect ORs and 95% CIs from studies assessing the association between the biomarker HER2 and pCR in NAC-treated breast cancer patients. I^2 and *p*-value for X^2 of heterogeneity are reported for each group analysis.

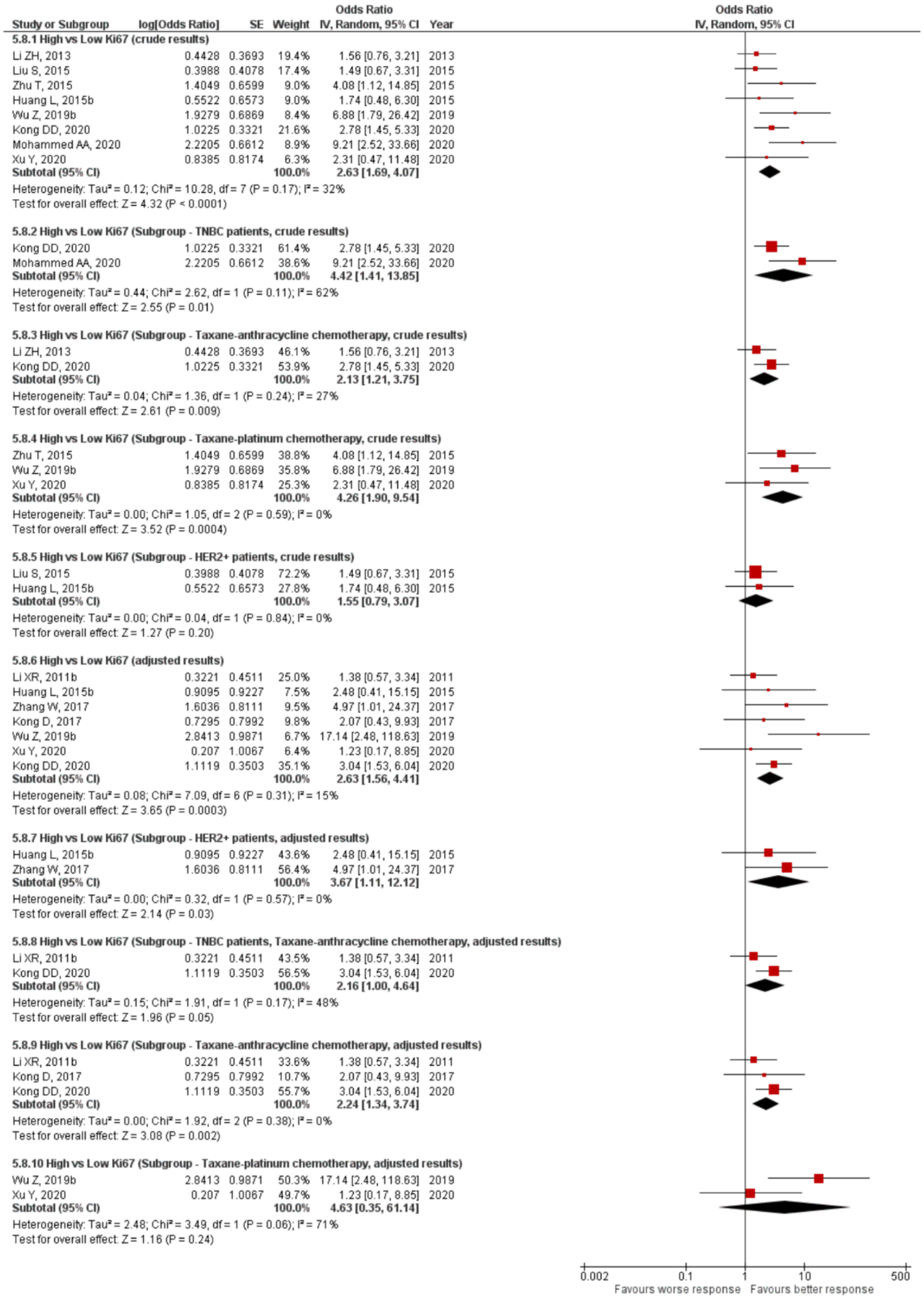

Supplementary Figure 7.7. Pooled reported association of pCR in NAC-treated Asian breast cancer patients with Ki-67. Forest plot describing the random effect ORs and 95% CIs from studies assessing the association between Ki-67 and pCR in NAC-treated breast cancer patients. I^2 and *p*-value for X^2 of heterogeneity are reported for each group analysis.

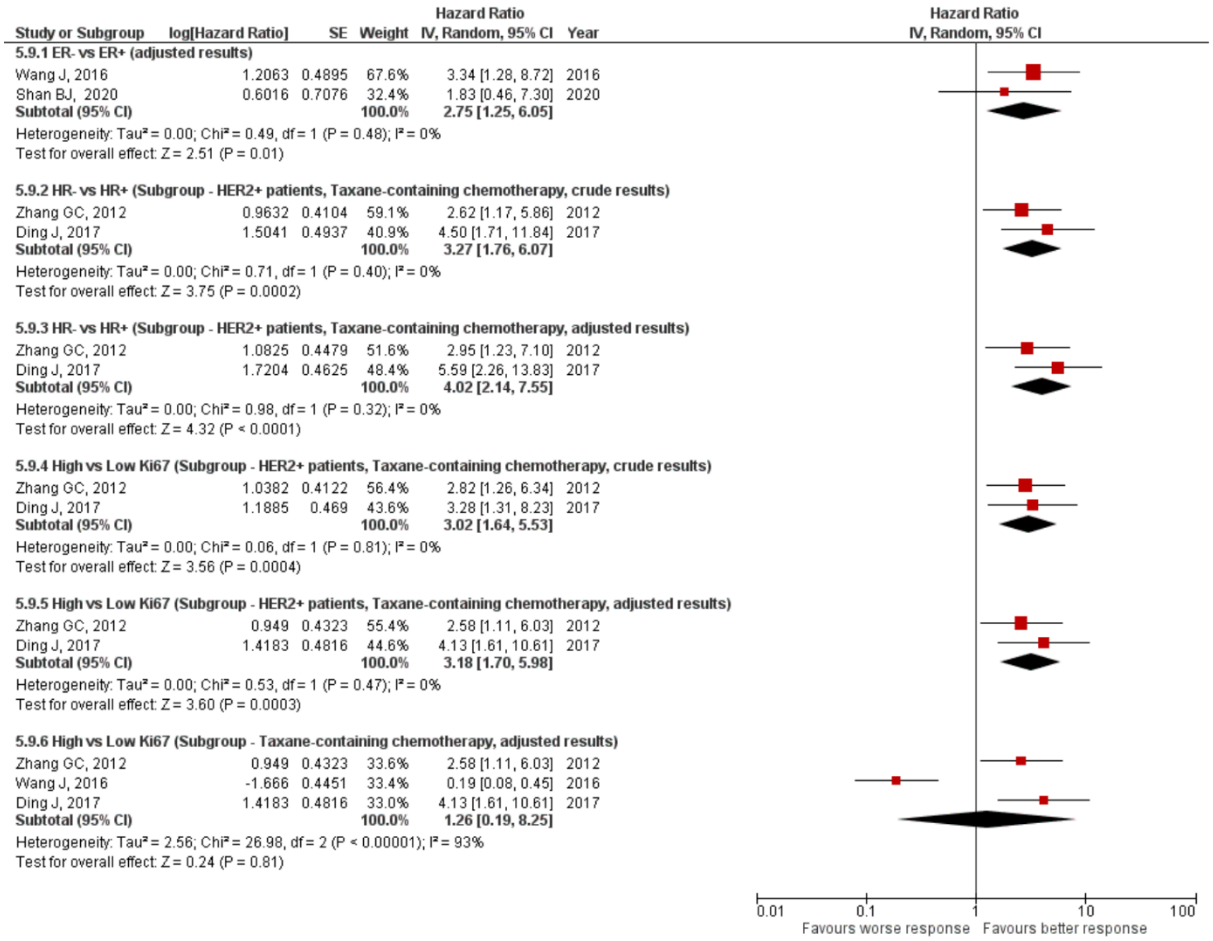

Supplementary Figure 7.8. Pooled reported association of pCR in NAC-treated Asian breast cancer patients presented in different biomarkers. Forest plot describing the random effect HRs and 95% CIs from studies assessing the association between pCR in NAC-treated breast cancer patients and the biomarkers ER, HR, and Ki-67. I^2 and *p*-value for X^2 of heterogeneity are reported for each group analysis.
