# Supplementary material for: Exploring the effectiveness of molecular subtypes, biomarkers, and genetic variations as first-line treatment predictors in Asian breast cancer patients: a systematic review and meta-analysis
Source: Syst Rev. 2024 Apr 4;13:100. doi: 10.1186/s13643-024-02520-5 (PMC10993489; doi:10.1186/s13643-024-02520-5)
Supplement: Supplementary file 8 — Additional file 8. Publication Bias. Supplementary Figure 8.1. Funnel plot of pooled pCR outcome of Asian breast cancer patients treated with TA chemotherapy. Funnel plot assessing the publication bias in evaluating the effect of HER2E and Luminal, combined in breast cancer pCR outcome of patients treated with TA regimen in the neoadjuvant setting. Supplementary Figure 8.2. Funnel plot of pooled pCR outcome of Asian breast cancer patients treated with TA chemotherapy. Funnel plot assessing the publication bias in evaluating the effect of TNBC and Luminal, combined in breast cancer pCR outcome of patients treated with TA regimen in the neoadjuvant setting. Supplementary Figure 8.3. Funnel plot of pooled pCR outcome of Asian breast cancer patients treated with TA chemotherapy. Funnel plot assessing the publication bias in evaluating the effect of TNBC and HER2E in breast cancer pCR outcome of patients treated with TA regimen in the neoadjuvant setting. Supplementary Figure 8.4. Funnel plot of pooled pCR outcome of Asian breast cancer patients treated with TP chemotherapy. Funnel plot assessing the publication bias in evaluating the effect of HER2E and Luminal, combined in breast cancer pCR outcome of patients treated with TP regimen in the neoadjuvant setting. Supplementary Figure 8.5. Funnel plot of pooled pCR outcome of Asian breast cancer patients treated with TA chemotherapy. Funnel plot assessing the publication bias in evaluating the effect of ER in breast cancer pCR outcome of patients treated with TA regimen in the neoadjuvant setting. Supplementary Figure 8.6. Funnel plot of pooled pCR outcome of Asian breast cancer patients treated with TA chemotherapy. Funnel plot assessing the publication bias in evaluating the effect of HER2 in breast cancer pCR outcome of patients treated with TA regimen in the neoadjuvant setting. Supplementary Figure 8.7. Funnel plot of pooled pCR outcome of Asian breast cancer patients treated with TA chemotherapy. Funnel plot assessi [file 13643_2024_2520_MOESM8_ESM.pdf]

PUBLICATION BIAS ASSESSMENT

1. HER2E vs Luminal, combined treated with taxane-anthracycline (TA)
   a. Publication bias assessment

Test Name	Value	*p*
Fail-Safe N	211.000	<0.001
Kendall's Tau	0.111	0.761
Egger's Regression	-0.230	0.818

Note. Fail-safe N calculation using the Rosenthal approach

   b. Funnel plot

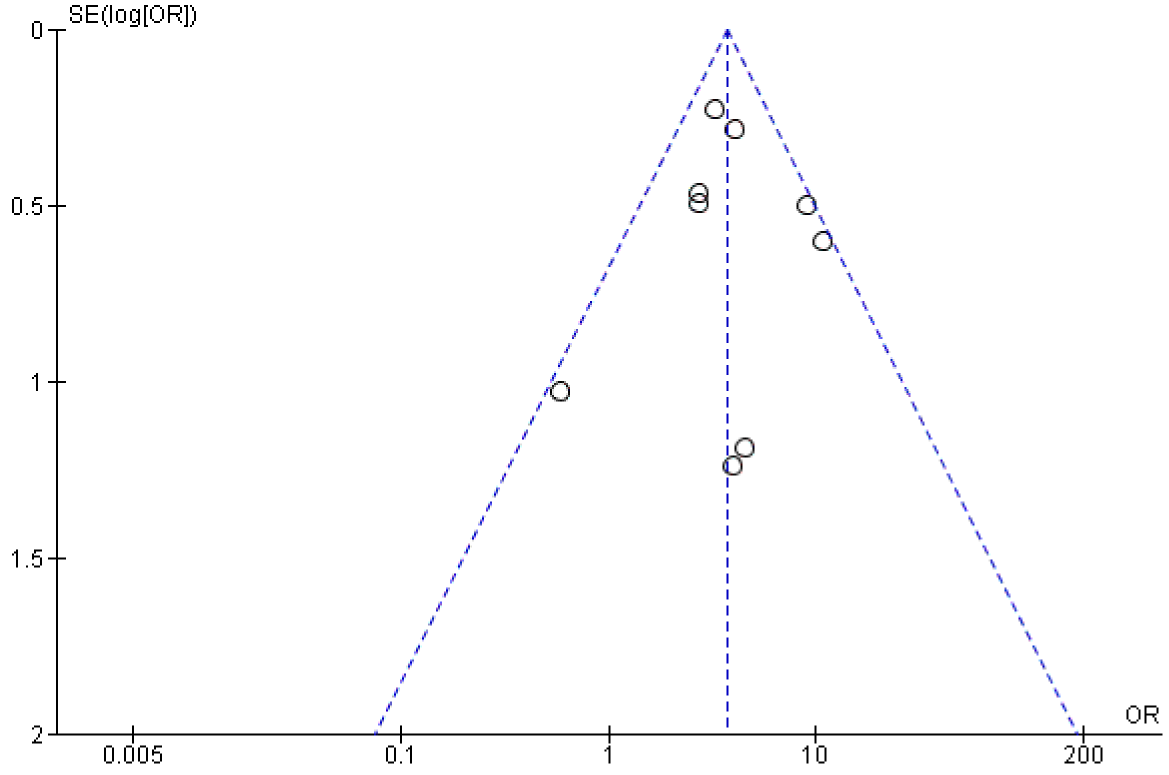

Supplementary Figure 8.1. Funnel plot of pooled pCR outcome of Asian breast cancer patients treated with TA chemotherapy. Funnel plot assessing the publication bias in evaluating the effect of HER2E and Luminal, combined in breast cancer pCR outcome of patients treated with TA regimen in the neoadjuvant setting.

2. TNBC vs Luminal, combined treated with taxane-anthracycline (TA)

a. Publication bias assessment

Test Name	Value	*p*
Fail-Safe N	143.000	<0.001
Kendall's Tau	-0.143	0.720
Egger's Regression	-0.537	0.591

Note. Fail-safe N calculation using the Rosenthal approach

b. Funnel plot

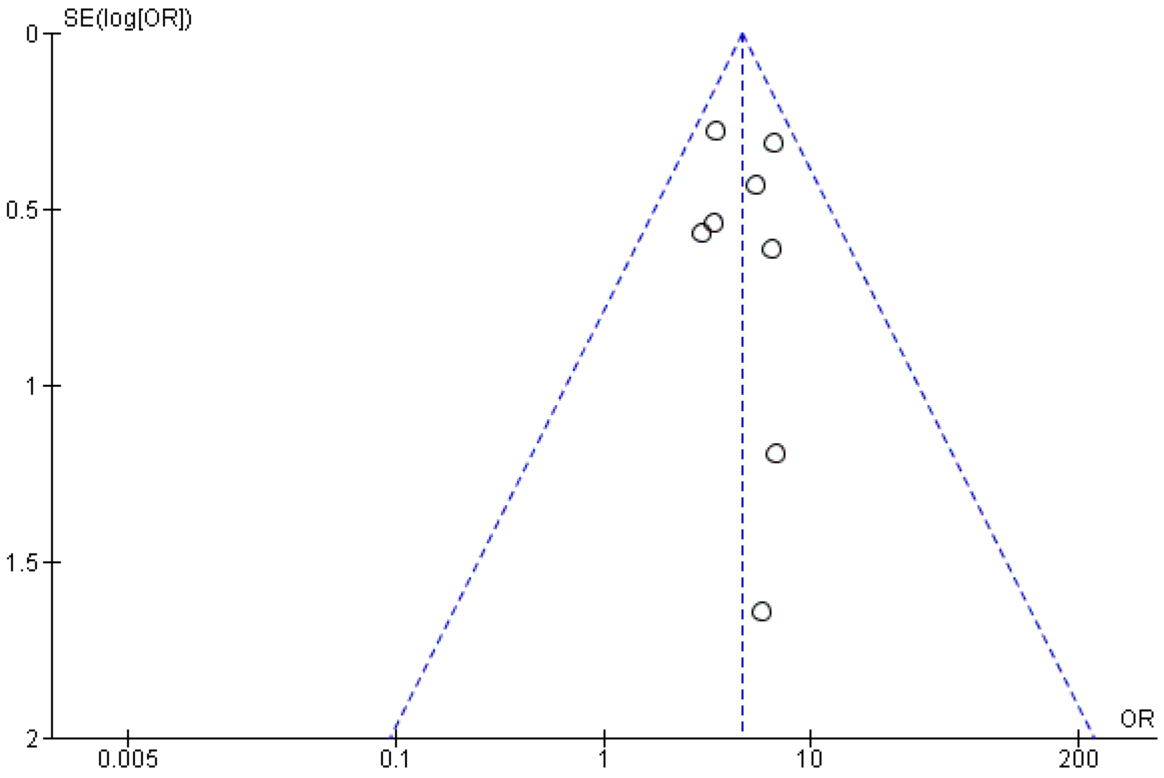

Supplementary Figure 8.2. Funnel plot of pooled pCR outcome of Asian breast cancer patients treated with TA chemotherapy. Funnel plot assessing the publication bias in evaluating the effect of TNBC and Luminal, combined in breast cancer pCR outcome of patients treated with TA regimen in the neoadjuvant setting.

3. TNBC vs HER2E treated with taxane-anthracycline (TA)

a. Publication bias assessment

Test Name	Value	*p*
Fail-Safe N	0.000	0.159
Kendall's Tau	0.071	0.905
Egger's Regression	0.158	0.875

Note. Fail-safe N calculation using the Rosenthal approach

b. Funnel plot

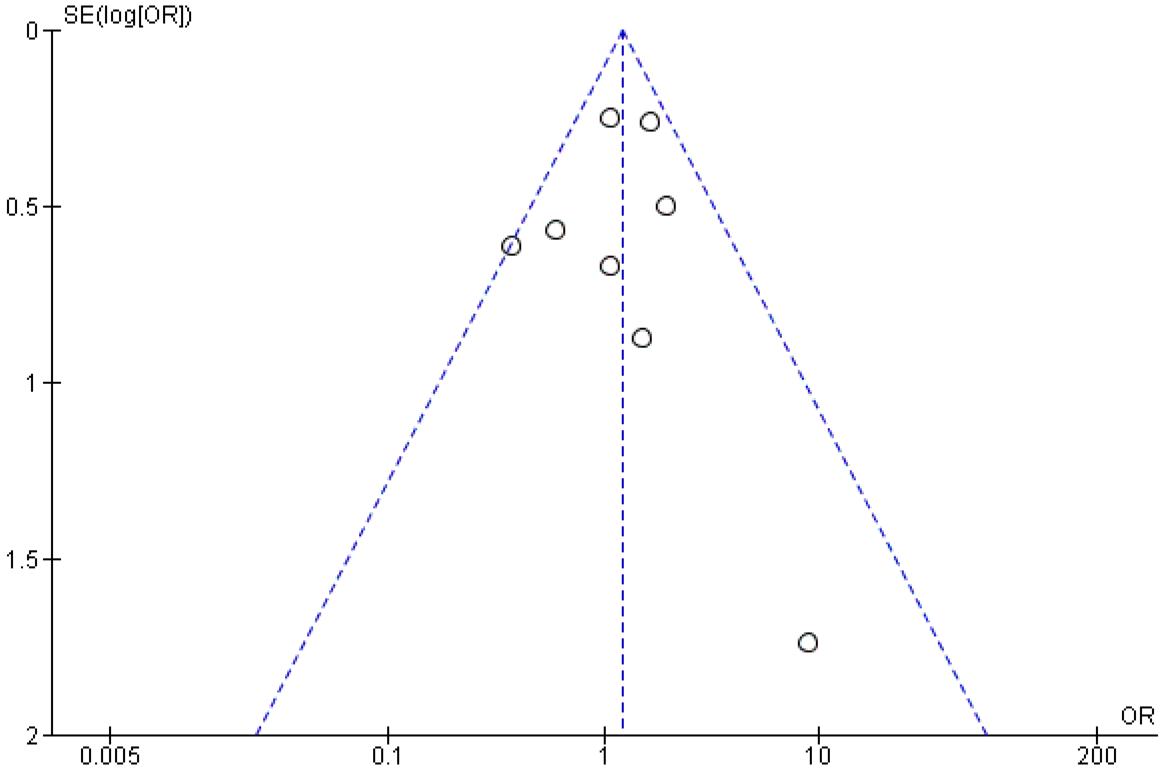

Supplementary Figure 8.3. Funnel plot of pooled pCR outcome of Asian breast cancer patients treated with TA chemotherapy. Funnel plot assessing the publication bias in evaluating the effect of TNBC and HER2E in breast cancer pCR outcome of patients treated with TA regimen in the neoadjuvant setting.

4. HER2E vs Luminal, combined treated with taxane-platinum (TP)

a. Publication bias assessment

Test Name	Value	*p*
Fail-Safe N	91.000	<0.001
Kendall's Tau	0.524	0.136
Egger's Regression	0.962	0.336

Note. Fail-safe N calculation using the Rosenthal approach

b. Funnel plot

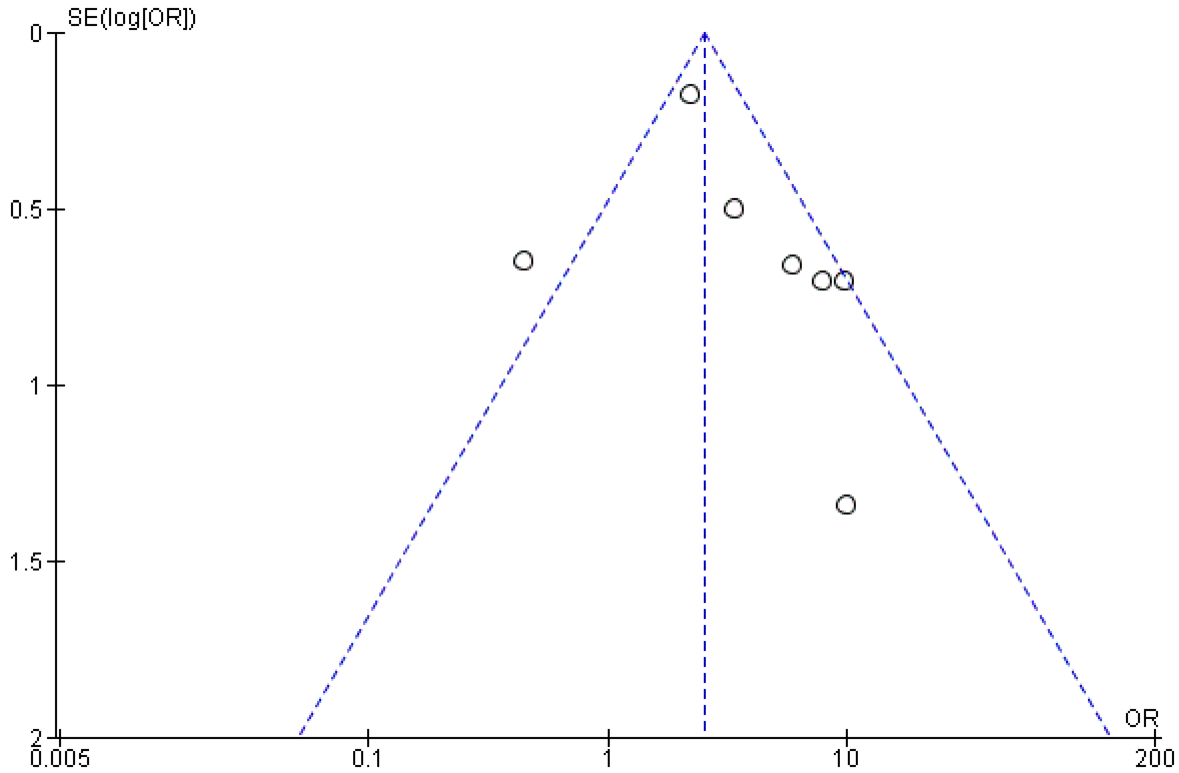

Supplementary Figure 8.4. Funnel plot of pooled pCR outcome of Asian breast cancer patients treated with TP chemotherapy. Funnel plot assessing the publication bias in evaluating the effect of HER2E and Luminal, combined in breast cancer pCR outcome of patients treated with TP regimen in the neoadjuvant setting.

5. ER- vs ER+ treated with taxane-anthracycline (TA)

a. Publication bias assessment

Test Name	Value	*p*
Fail-Safe N	117.000	<0.001
Kendall's Tau	0.200	0.719
Egger's Regression	-0.728	0.466

Note. Fail-safe N calculation using the Rosenthal approach

b. Funnel plot

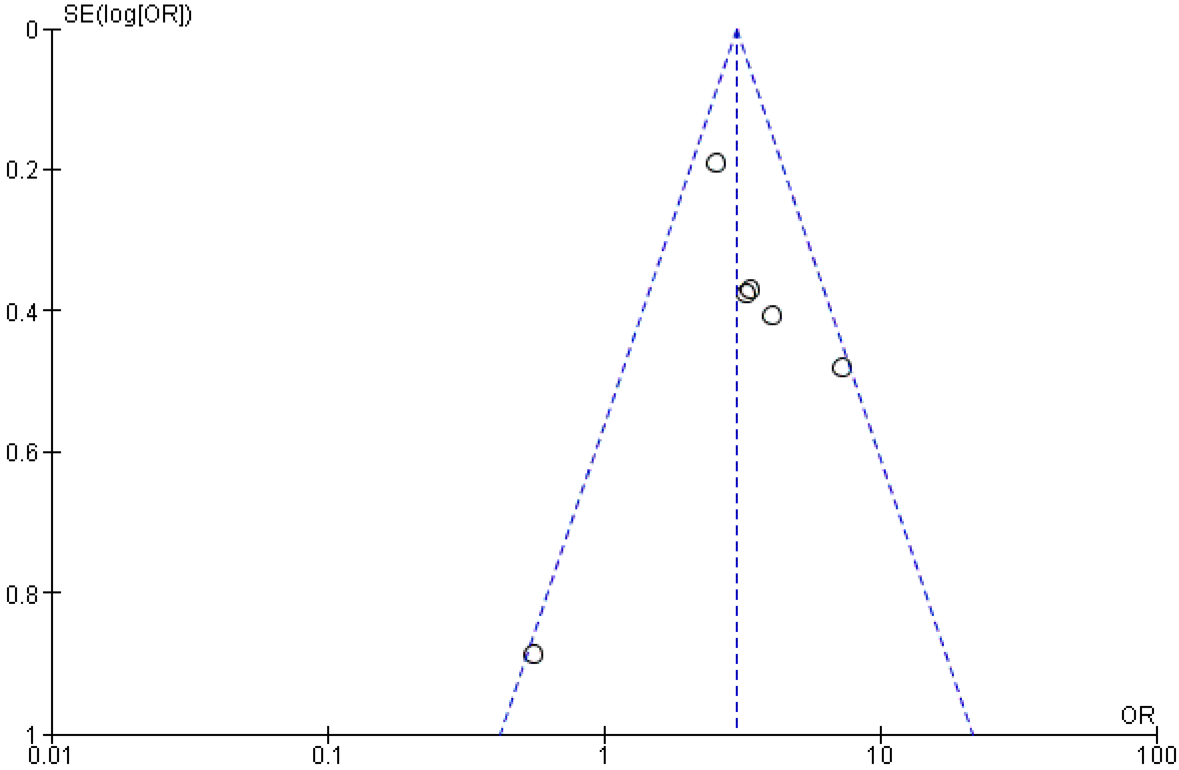

Supplementary Figure 8.5. Funnel plot of pooled pCR outcome of Asian breast cancer patients treated with TA chemotherapy. Funnel plot assessing the publication bias in evaluating the effect of ER in breast cancer pCR outcome of patients treated with TA regimen in the neoadjuvant setting.

6. HER2+ vs HER2- treated with taxane-anthracycline (TA)

a. Publication bias assessment

Test Name	Value	*p*
Fail-Safe N	51.000	<0.001
Kendall’s Tau	-0.257	0.343
Egger’s Regression	-2.224	0.026

Note. Fail-safe N calculation using the Rosenthal approach

b. Funnel plot

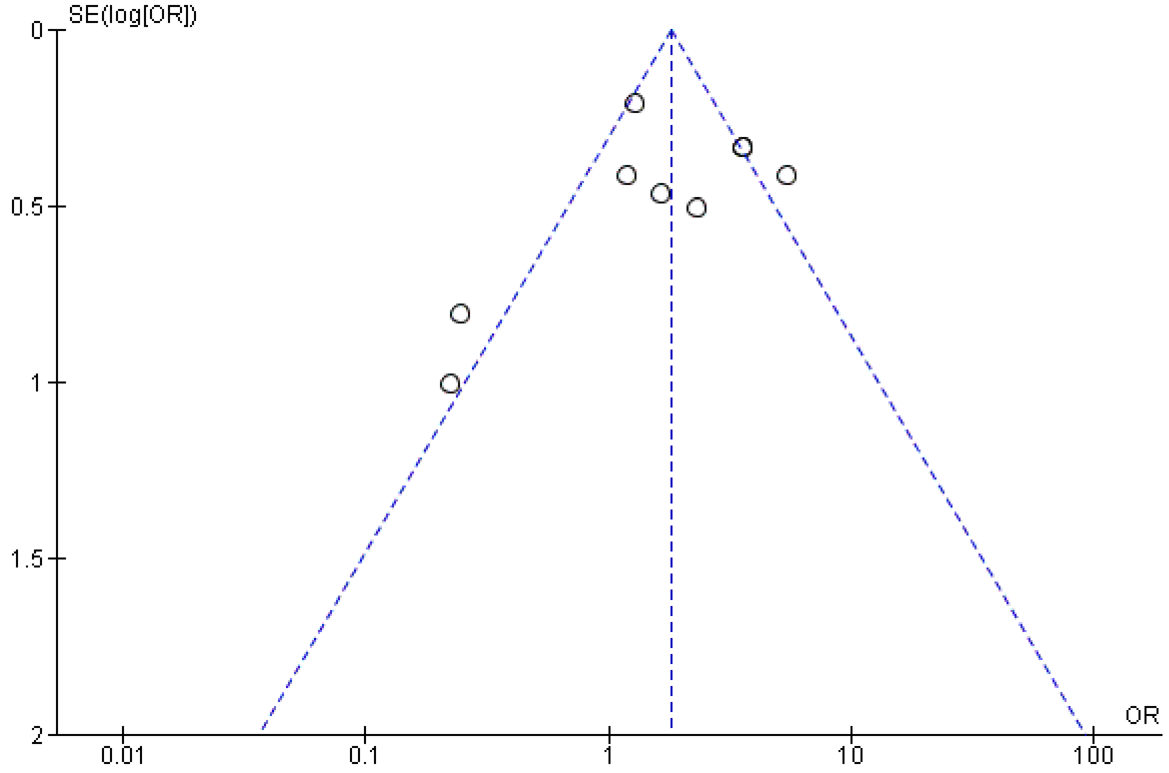

Supplementary Figure 8.6. Funnel plot of pooled pCR outcome of Asian breast cancer patients treated with TA chemotherapy. Funnel plot assessing the publication bias in evaluating the effect of HER2 in breast cancer pCR outcome of patients treated with TA regimen in the neoadjuvant setting.

7. High vs low Ki-67 treated with taxane-anthracycline (TA)

a. Publication bias assessment

Test Name	Value	*p*
Fail-Safe N	149.000	<0.001
Kendall's Tau	0.273	0.250
Egger's Regression	1.309	0.191

Note. Fail-safe N calculation using the Rosenthal approach

b. Funnel plot

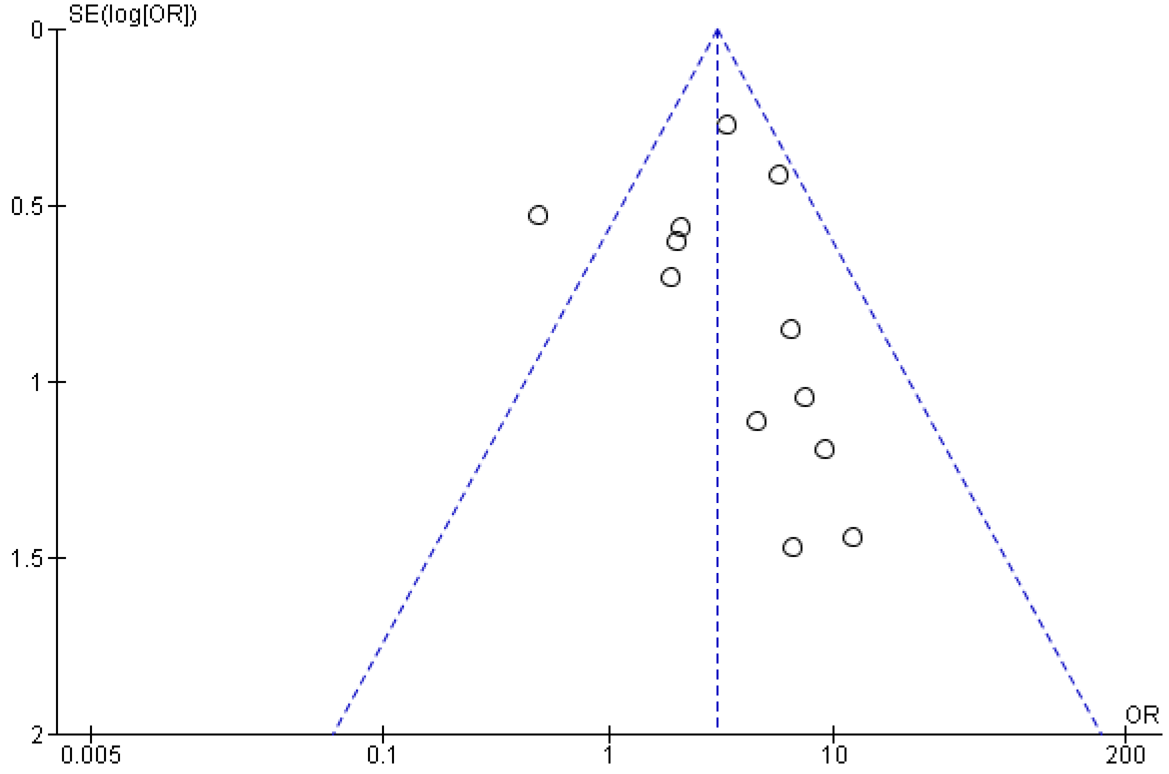

Supplementary Figure 8.7. Funnel plot of pooled pCR outcome of Asian breast cancer patients treated with TA chemotherapy. Funnel plot assessing the publication bias in evaluating the effect of Ki-67 in breast cancer pCR outcome of patients treated with TA regimen in the neoadjuvant setting.

8. ER+ vs ER- (adjusted results)

a. Publication bias assessment

Test Name	Value	*p*
Fail-Safe N	33.000	0.001
Kendall's Tau	0.033	0.914
Egger's Regression	-0.167	0.867

Note. Fail-safe N calculation using the Rosenthal approach

b. Funnel plot

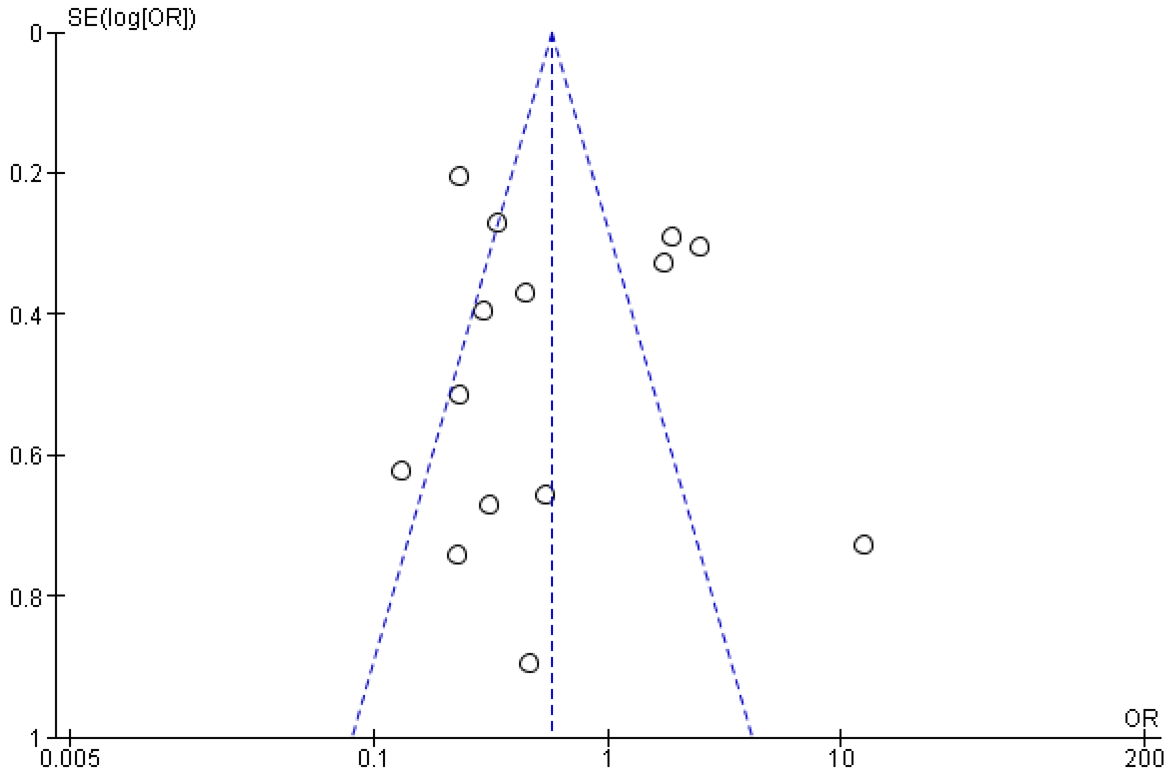

Supplementary Figure 8.8. Funnel plot of pooled pCR association of Asian breast cancer patients in the neoadjuvant setting. Funnel plot assessing the publication bias in evaluating the effect of ER+ and ER- (adjusted OR association) in breast cancer pCR outcome of patients treated in the neoadjuvant setting.

9. PR- vs PR+ (adjusted results)

a. Publication bias assessment

Test Name	Value	*p*
Fail-Safe N	0.000	0.349
Kendall's Tau	-0.643	0.031
Egger's Regression	-1.756	0.079

Note. Fail-safe N calculation using the Rosenthal approach

b. Funnel plot

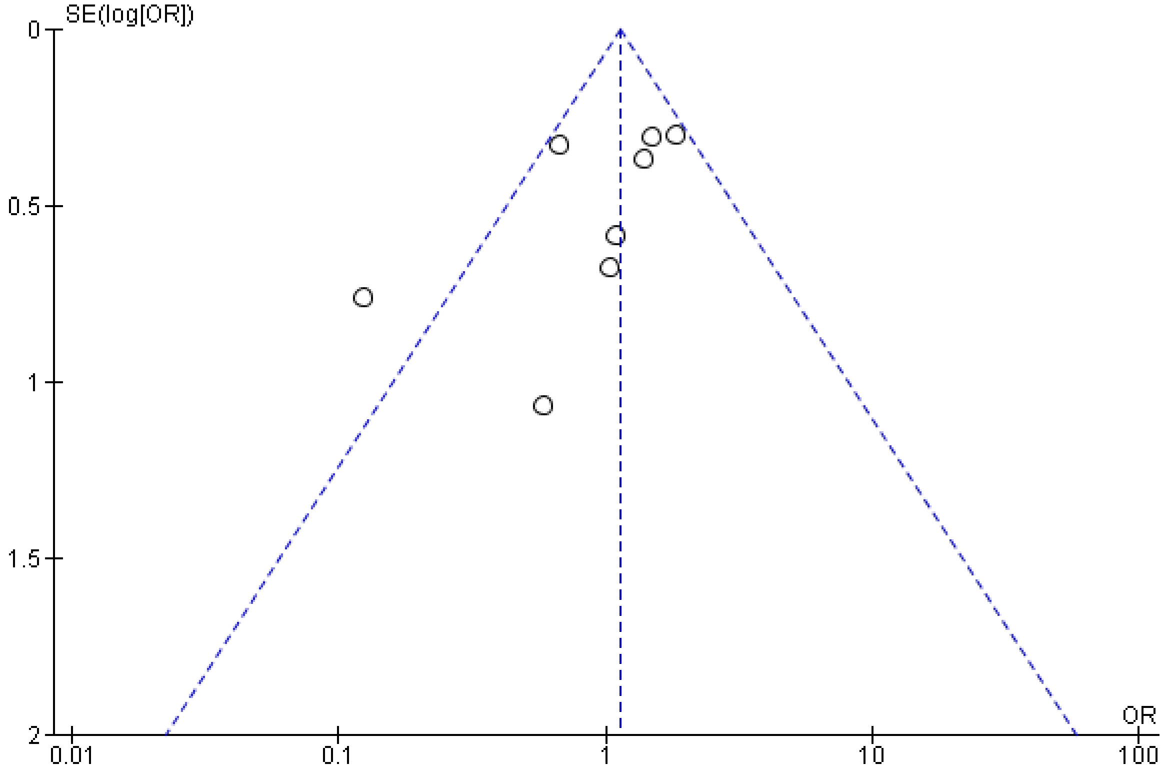

Supplementary Figure 8.9. Funnel plot of pooled pCR association of Asian breast cancer patients in the neoadjuvant setting. Funnel plot assessing the publication bias in evaluating the effect of PR- and PR+ (adjusted OR association) in breast cancer pCR outcome of patients treated in the neoadjuvant setting.

10. HER2- vs HER2+ (crude results)

a. Publication bias assessment

Test Name	Value	*p*
Fail-Safe N	25.000	<0.001
Kendall's Tau	-0.143	0.773
Egger's Regression	-0.609	0.543

Note. Fail-safe N calculation using the Rosenthal approach

b. Funnel plot

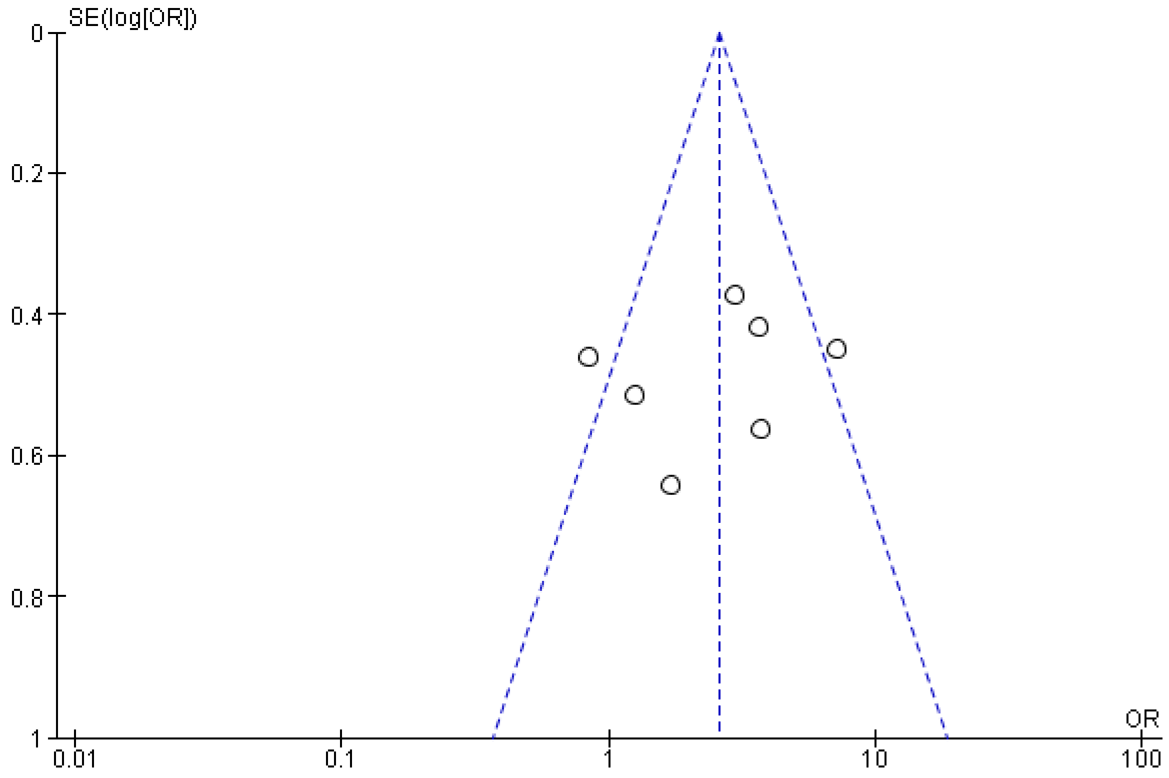

Supplementary Figure 8.10. Funnel plot of pooled pCR association of Asian breast cancer patients in the neoadjuvant setting. Funnel plot assessing the publication bias in evaluating the effect of HER2- and HER2+ (crude OR association) in breast cancer pCR outcome of patients treated in the neoadjuvant setting.

11. HER2+ vs HER2- (adjusted results)

a. Publication bias assessment

Test Name	Value	*p*
Fail-Safe N	97.000	<0.001
Kendall's Tau	0.000	1.000
Egger's Regression	0.888	0.375

Note. Fail-safe N calculation using the Rosenthal approach

b. Funnel plot

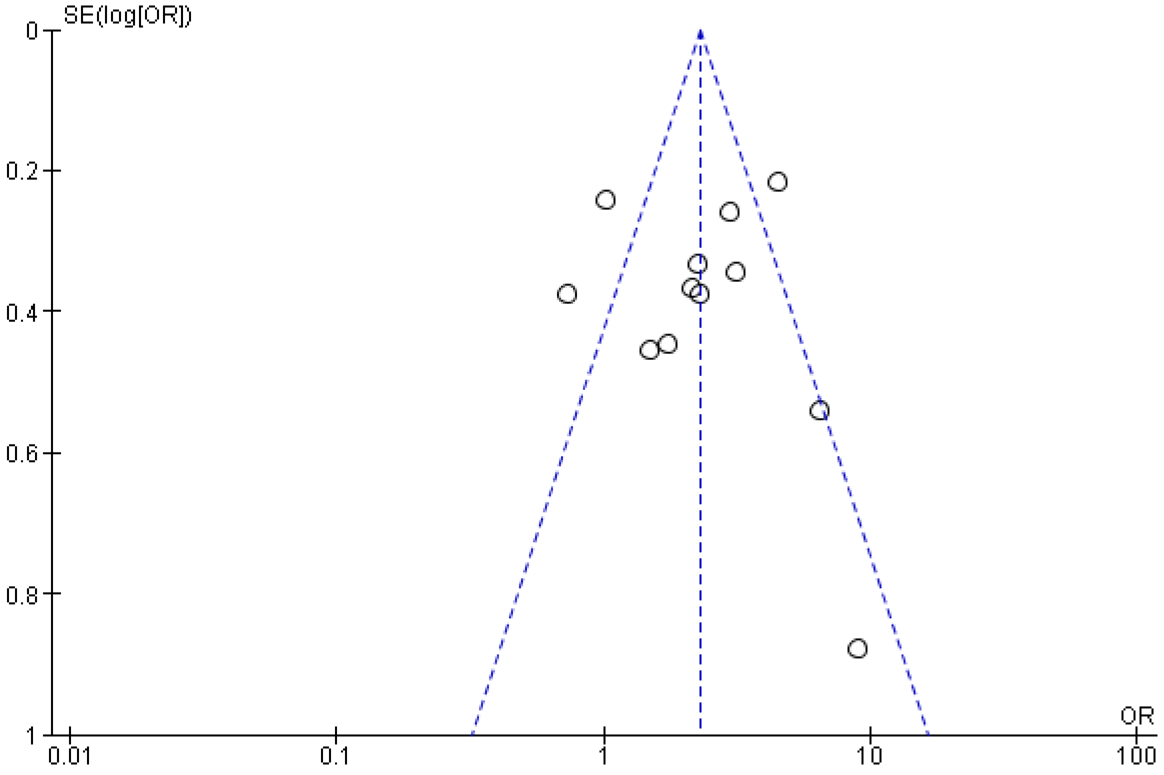

Supplementary Figure 8.11. Funnel plot of pooled pCR association of Asian breast cancer patients in the neoadjuvant setting. Funnel plot assessing the publication bias in evaluating the effect of HER2+ and HER2- (adjusted OR association) in breast cancer pCR outcome of patients treated in the neoadjuvant setting.

12. High vs low Ki-67 (crude results)

a. Publication bias assessment

Test Name	Value	*p*
Fail-Safe N	42.000	<0.001
Kendall's Tau	0.357	0.275
Egger's Regression	1.220	0.223

Note. Fail-safe N calculation using the Rosenthal approach

b. Funnel plot

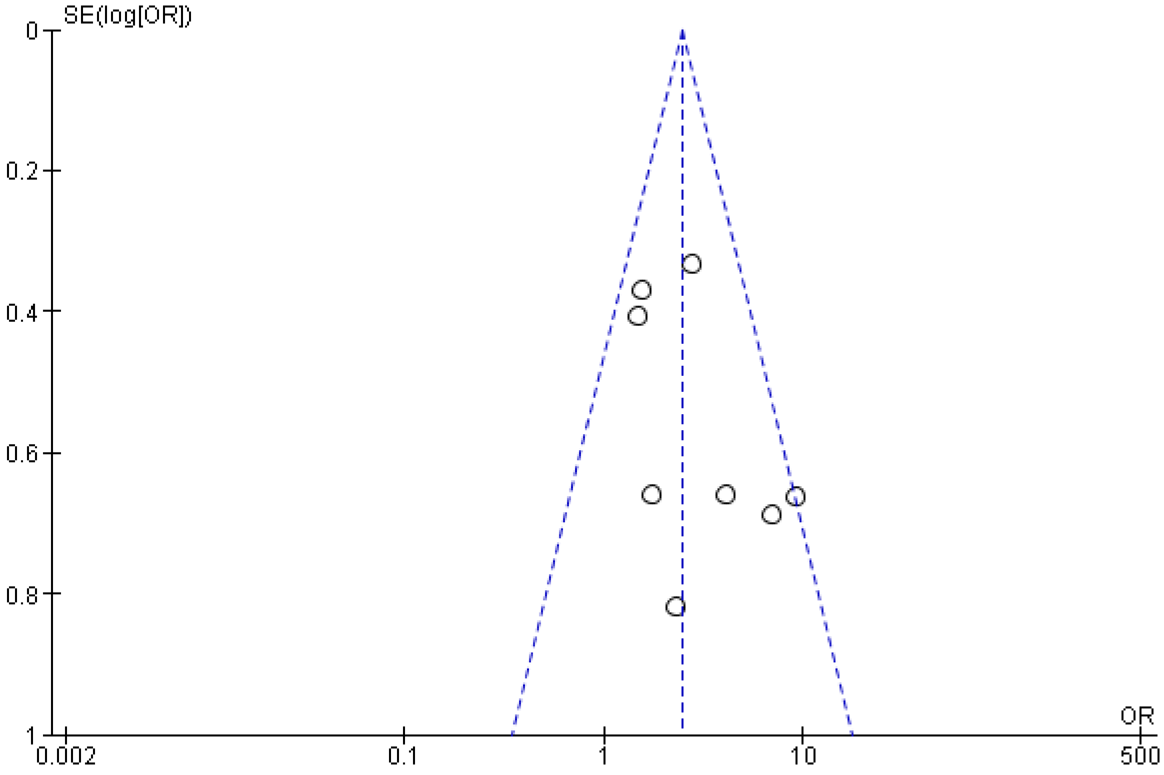

Supplementary Figure 8.12. Funnel plot of pooled pCR association of Asian breast cancer patients in the neoadjuvant setting. Funnel plot assessing the publication bias in evaluating the effect of high and low Ki-67 (crude OR association) in breast cancer pCR outcome of patients treated in the neoadjuvant setting.

13. High vs low Ki-67 (adjusted results)

a. Publication bias assessment

Test Name	Value	*p*
Fail-Safe N	23.000	<0.001
Kendall's Tau	0.143	0.773
Egger's Regression	0.647	0.518

Note. Fail-safe N calculation using the Rosenthal approach

b. Funnel plot

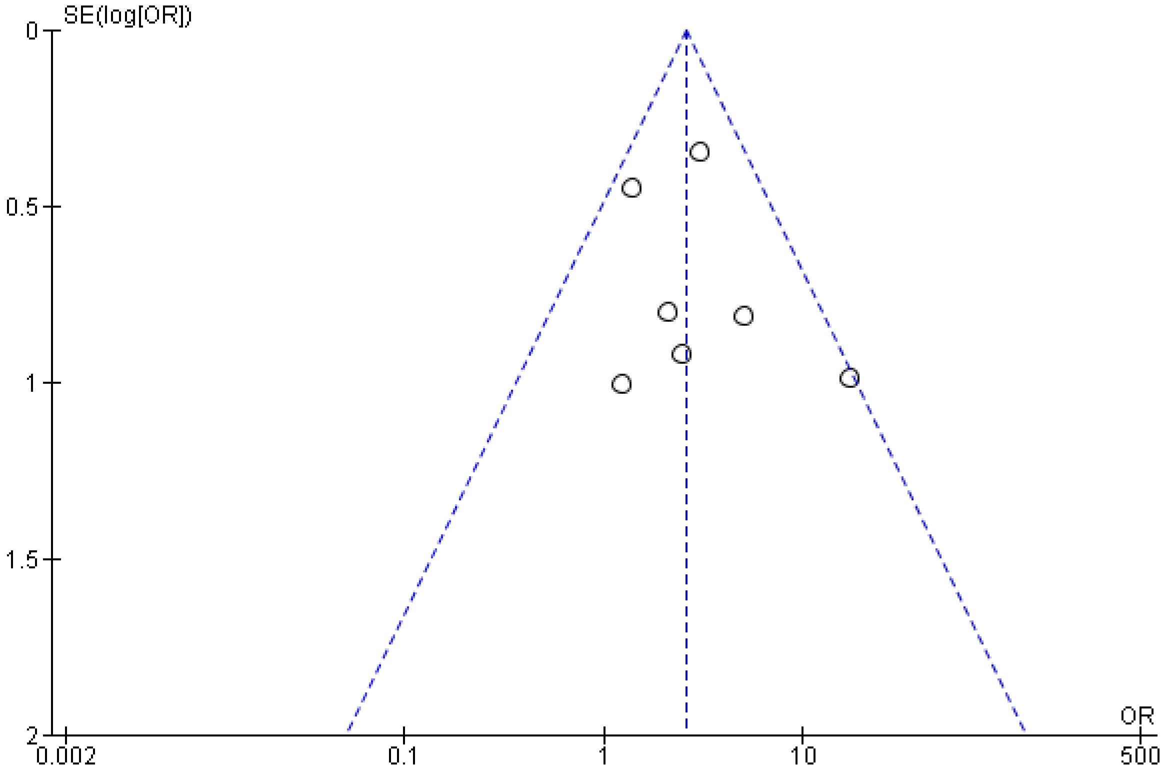

Supplementary Figure 8.13. Funnel plot of pooled pCR association of Asian breast cancer patients in the neoadjuvant setting. Funnel plot assessing the publication bias in evaluating the effect of high and low Ki-67 (adjusted OR association) in breast cancer pCR outcome of patients treated in the neoadjuvant setting.
